# Second Generation Sequencing of the Mesothelioma Tumor Genome

**DOI:** 10.1371/journal.pone.0010612

**Published:** 2010-05-13

**Authors:** Raphael Bueno, Assunta De Rienzo, Lingsheng Dong, Gavin J. Gordon, Colin F. Hercus, William G. Richards, Roderick V. Jensen, Arif Anwar, Gautam Maulik, Lucian R. Chirieac, Kim-Fong Ho, Bruce E. Taillon, Cynthia L. Turcotte, Robert G. Hercus, Steven R. Gullans, David J. Sugarbaker

**Affiliations:** 1 The International Mesothelioma Program, Brigham and Women's Hospital, Boston, Massachusetts, United States of America; 2 Division of Thoracic Surgery, Brigham and Women's Hospital, Boston, Massachusetts, United States of America; 3 Department of Pathology, Brigham and Women's Hospital, Boston, Massachusetts, United States of America; 4 Harvard Medical School, Boston, Massachusetts, United States of America; 5 454 Life Sciences, Inc., Branford, Connecticut, United States of America; 6 Synamatix, Kuala Lumpur, Malaysia; 7 Department of Biological Sciences, Virginia Tech, Blacksburg, Virginia, United States of America; 8 Excel Medical Ventures, Boston, Massachusetts, United States of America; Innsbruck Medical University, Austria

## Abstract

The current paradigm for elucidating the molecular etiology of cancers relies on the interrogation of small numbers of genes, which limits the scope of investigation. Emerging second-generation massively parallel DNA sequencing technologies have enabled more precise definition of the cancer genome on a global scale. We examined the genome of a human primary malignant pleural mesothelioma (MPM) tumor and matched normal tissue by using a combination of sequencing-by-synthesis and pyrosequencing methodologies to a 9.6X depth of coverage. Read density analysis uncovered significant aneuploidy and numerous rearrangements. Method-dependent informatics rules, which combined the results of different sequencing platforms, were developed to identify and validate candidate mutations of multiple types. Many more tumor-specific rearrangements than point mutations were uncovered at this depth of sequencing, resulting in novel, large-scale, inter- and intra-chromosomal deletions, inversions, and translocations. Nearly all candidate point mutations appeared to be previously unknown SNPs. Thirty tumor-specific fusions/translocations were independently validated with PCR and Sanger sequencing. Of these, 15 represented disrupted gene-encoding regions, including kinases, transcription factors, and growth factors. One large deletion in *DPP10* resulted in altered transcription and expression of *DPP10* transcripts in a set of 53 additional MPM tumors correlated with survival. Additionally, three point mutations were observed in the coding regions of *NKX6-2*, a transcription regulator, and *NFRKB*, a DNA-binding protein involved in modulating *NFKB1*. Several regions containing genes such as *PCBD2* and *DHFR*, which are involved in growth factor signaling and nucleotide synthesis, respectively, were selectively amplified in the tumor. Second-generation sequencing uncovered all types of mutations in this MPM tumor, with DNA rearrangements representing the dominant type.

## Introduction

Malignant pleural mesothelioma (MPM) is a highly aggressive pleural tumor associated with asbestos exposure. Therapeutic options are limited and median patient survival is about 7 months. The standard chemotherapy regimen consisting of pemetrexed and platinum extends median survival by approximately 2 months [1]; however, select patients who undergo complete surgical resection followed by chemotherapy and radiation derive a longer survival benefit with some surviving over 5 years [2], [3]. To date, most clinical trials have focused on cytotoxic agents rather than targeted therapies [4]–[6].

While it has been shown that chromosomal abnormalities are abundant within MPM [7]–[11], whole genome analysis of this tumor has not yet been described. Massively parallel second-generation sequencing methodologies using pyrosequencing or derivations of sequencing by synthesis have paved the way for large-scale analyses of tumor biology by targeted gene re-sequencing, mutation detection, copy number variation (CNV), single nucleotide polymorphism (SNP), changes in chromatin architecture, and epigenetic changes such as methylation pattern alteration [12]–[24]. The ability to analyze entire genomes opens the door to global mapping of normal variation and mutations of all types for correlation with disease propensity, diagnosis, treatment, prognosis, as well as identification of new targets for interventional therapy discovery and development [15], [25], [26].

We report a definition at the genomic level of a primary human MPM tumor using a combination of approaches, namely, Illumina sequencing by synthesis and Roche/454 pyrosequencing [18], [19], [27]. Building upon previous work in which we sequenced the transcriptomes of four MPM patient samples with Roche/454 technology [26], we selected the genomic DNA (gDNA) from a tumor and normal control from one of those patients for more in-depth analysis. In this single tumor, we found hundreds of previously unreported single nucleotide variants (SNVs), single nucleotide insertions/deletions (indels), inter- and intra-chromosomal large-scale DNA rearrangements (including many that occur within genes), and translocations. Many of the aberrations are tumor-specific mutations occurring at loci that code for genes involved in distinct pathways known to play key roles in cancer initiation and progression. We also found substantial variability in this individual's normal genome, confirming previous reports [21], [28]–[31]. The data herein suggest that the numbers and types of variations both in the normal and the MPM tumor genomes are considerable, and further, that efforts to use these data (and similar data from other solid tumors) to elucidate tumor biology and identify novel candidate therapeutic targets may be more challenging than previously thought.

## Results

### Sequencing and mapping

**Figure 1:**
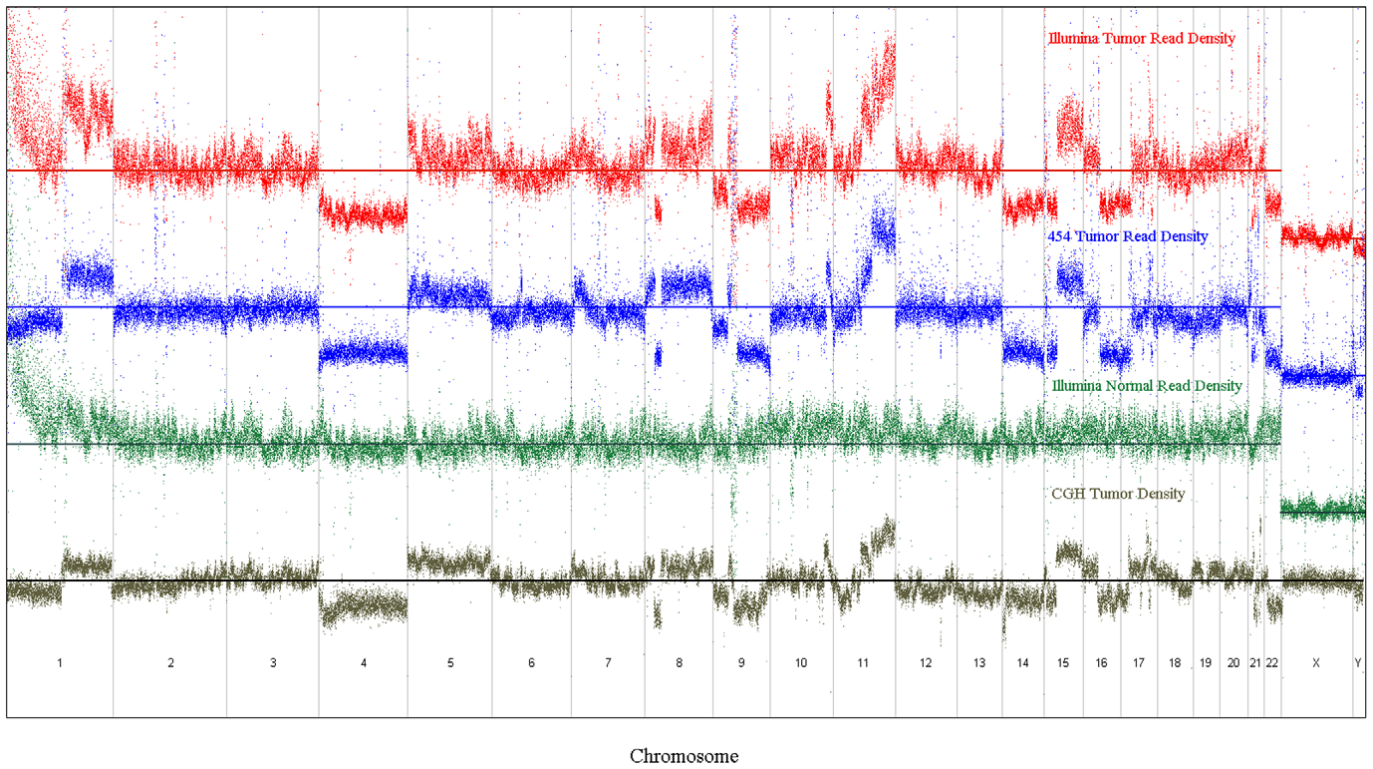
Sequence read mapping to the reference genome to display the Karyotype of the patient tumor and normal DNA by Illumina and Roche/454 sequencing and by CGH.

**Figure 2:**
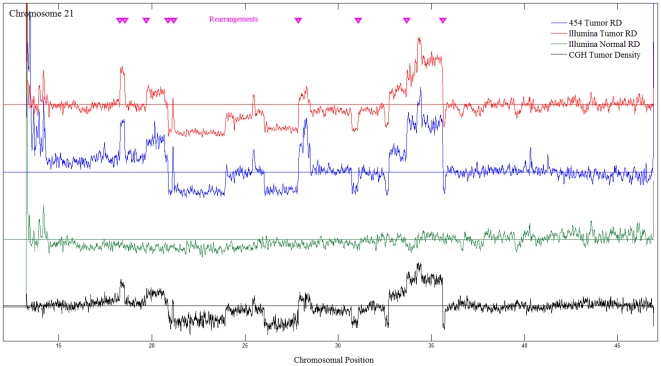
The read density of the long arm of chromosome 21 in tumor and normal with the validated rearrangements.

We sequenced MPM tumor gDNA and matched normal lung gDNA using Illumina paired-ends (PE) technology to generate 17.8 and 15.67 Gigabase pairs (Gbp) or 5.6X and 5.2X coverage of the respective genomes (Table 1). Greater than 97% of the individual sequenced reads aligned to NCBI's RefSeq database. The tumor's read density when compared to the RefSeq database revealed numerous chromosomal CNVs (Fig. 1), a known hallmark of many tumor types including MPM [9], [21], [28], [29], [32]–[35]. Several chromosomes (2, 3, 6, 7, 10, 12, 13 and 20) appeared to be mostly euploid, whereas others, including Y, 4, 14, 18, 19 and 10, were less abundant than expected, or appeared to have extra copies (e.g., chromosome 5). Furthermore, a number of chromosomes (1, 8, 9, 11, 15, 16, 17, 21, and 22) appeared to have complex structures that included both haploid and polyploid segments. The read density/CNV were independently verified using deep exploration of the same samples with comparative genomic hybridization (CGH) arrays (Fig. 2) and were shown to match in a highly statistically significant manner (Pearson correlation coefficient (ρ) = 0.7918; 99% confidence interval  = 0.788≤ρ≤0.796). Thus, whole genome sequence analysis revealed as expected that this MPM tumor displayed large-scale aneuploidy.

**Table 1:** Mapping of Illumina Paired-End Sequence Data.

	Tumor		Normal	
**Total reads (PE)**	219,359,022		175,965,242	
**Total base pair**	17,808,799,058		15,673,204,592	
**Avg. length/PE read**	81		89	
**Mapping attributes of Paired-end reads (Read 1: Read 2)**	**# of mapped reads**	**%**	**# of mapped reads**	**%**
**1∶1**	153,601,517	70.02%	131,604,101	74.79%
**0∶1**	1,972,379	0.90%	1,435,061	0.82%
**1∶0**	12,270,045	5.59%	4,109,024	2.34%
**N:1**	12,108,970	5.52%	9,250,544	5.26%
**1:N**	11,930,579	5.44%	8,867,264	5.04%
**N:0**	2,201,869	1.00%	705,230	0.40%
**0:N**	508,749	0.23%	357,149	0.20%
**N:N**	18,242,362	8.32%	14,769,141	8.39%
***Total mapped reads***	***212,836,470***	***97.03%***	***171,097,514***	***97.23%***

1, reads maps to unique region; 0, read does not map to any region; N, read maps to multiple regions.

### Global validation using Roche/454 sequencing

Given the degree of aneuploidy and the large number of variants observed with Illumina sequencing in both the tumor and normal gDNAs, it became clear that independent validation or at least prioritization of candidate tumor mutations would be required to identify true positive mutations. Furthermore, second-generation sequencing methodologies possess inherent limitations related to artifacts and biases representing false-positive mutations [18], [19], [27], [36]. Thus, a pyrosequencing approach (Roche/454) was undertaken to confirm the read densities observed and the presence of any mutations detected. Roche/454 sequencing of the tumor gDNA generated 10.8 Gbp or ∼4X coverage, extending the total coverage of the tumor genome to nearly 10X. Ninety-six percent of the Roche/454 reads mapped to known human DNA and the read density analysis was highly statistically correlated with that obtained by Illumina sequencing and the CGH arrays (Figs. 1 and 2 and File S1).

### Genomic rearrangement

The dramatic changes in read density observed in the MPM tumor (Figs. 1 and 2) led us to focus on the identification of candidate sites of inter- and intra-chromosomal rearrangements which could represent inversions, deletions, insertions, and translocations (File S2). For this analysis, the only variants that were initially considered were those that were at least 1,000 bases apart from expected using the normal genome sequence as a reference. With Illumina data only, an analysis of all PE reads for those not located in the expected proximity to each other revealed 423,310 candidate chromosomal rearrangements in the tumor and 443,087 in the normal sequences. However, only 2,432 rearrangements were common to the tumor and normal sequences, a number more in-line with previous reports on variation in the normal genome [21], [28], [37]. Furthermore, the number of rearrangements supported by increasing numbers of reads was much lower, with only 485 in the tumor and 349 in the normal sequence observed at a depth of ≥4 reads, for example. PCR validation of a subset of rearrangements supported by fewer than 4 reads failed to identify any true positives, suggesting that the majority of these candidate rearrangements may be false positives possibly due to artifacts of the technology [27], [36], [38] and/or mutations present in a small number of tumor cells.

The Roche/454 method provided longer reads (∼220 bp), where a single read would be more likely to contain a translocation-junction [18], [19]. Similar to the findings above, the number of candidate rearrangements was reduced to 1,524 at a depth of 2 or more reads consistent with the hypothesis that pyrosequencing may also produce artifactual rearrangements [18], [19], [31].

We used two different approaches to prioritize for validation the best candidates for true tumor-specific rearrangements. The first approach enriched for chromosomal regions exhibiting read density shifts. To find rearrangements, a combination of Roche/454 and CGH data were used to identify junctions away from areas of repeat sequences that were unique to the tumor, particularly where the junctions were grossly apparent (e.g., Chr 21, Fig. 2). Using this approach, candidate Roche/454 rearrangements were ranked for subsequent independent validation using PCR and traditional Sanger sequencing. The top 33 candidate rearrangements were validated by Sanger sequencing. Of these, 21 were found to be true mutations, i.e., present in tumor but not in the matched normal gDNA, and 12 were present in both the tumor and normal genomes consistent with polymorphisms. In the second approach, the Illumina and Roche/454 data were combined, and 105 tumor-specific rearrangements were observed that were common to the two methods at a depth of at least 1 read in each sequencing modality. These included 12 of the 21 tumor-specific mutations validated as described above. The remaining 93 candidate rearrangements were examined further by Sanger sequencing. Of these, 9 were tumor-specific mutations, 81 were previously unknown variants present in both tumor and normal, and 3 could not be confirmed. In total, 30 different tumor-specific rearrangements were ultimately validated. Of these, 15 rearrangements disrupted 17 genes (Table 2, Table S1, and File S2). Six of the tumor-specific validated rearrangements were inter-chromosomal and 24 were intra-chromosomal. A set of 53 MPM tumors was examined at the genomic level for all 30 tumor-specific rearrangements using PCR amplification. None of these specific rearrangements was found to be present in any additional tumor genomes in the exact breakpoints discovered in our patient. One of the rearrangements resulted in a large deletion within the dipeptidyl-peptidase 10 (*DPP10*) gene (exons 4–25) and produced the expected truncated fusion transcript uniquely in the transcriptome of the tumor, but not of the normal lung.

**Table 2:** Rearrangement Mutations.

Chromosomal Break Point (bp)	Chromosomal Break Point (bp)	Gap Length (bp)	No. of Reads	Disrupted Gene(s)	Break Point Location in Gene(s)	Gene Name/Function
**Intrachromosomal**						
Chr2:115918613	Chr2:116314797	396183	4	DPP10	Exons 4–24 deleted	K^+^ Channel Modulator
Chr3:98917287	Chr3:99007383	90095	3	EPHA6	Intron 18	EPH receptor A6
Chr6:64615701	Chr6:57319605	7296095	1	EYS/PRIM2	Intron 36/Intron 5	Eyes shut homolog (Drosophila)/Primase, DNA, polypeptide 2 (58kDa)
Chr8:6749979	Chr8:34877265	28127285	1	None	NA	NA
Chr8:6786552	Chr8:34863640	28077087	3	None	NA	NA
Chr8:21254202	Chr8:72593210	51339007	1	None	NA	NA
Chr8:34816557	Chr8:72573255	37756697	1	None	NA	NA
Chr8:34816593	Chr8:72577581	37760987	3	None	NA	NA
Chr8:36550070	Chr8:72552661	36002590	3	None	NA	NA
Chr9:21957500	Chr9:22075035	117534	1	None	NA	NA
Chr10:84083200	Chr10:84157179	73978	1	NRG3	Intron 1/Inton 2	Neuregulin Growth Factor
Chr11:55762996	Chr11:55765854	2857	1	None	NA	NA
Chr11:59018943	Chr11:59023522	4578	1	None	NA	NA
Chr12:127300384	Chr12:126530113	770270	1	None	NA	NA
Chr17:23113949	Chr17:65721910	42607960	4	NOS2A	Intron 22	Nitric Oxide Synthase
Chr17:26853914	Chr17:47815374	20961459	2	RAB11FIP4	Intron 3	Endosomal Trafficking
Chr17:47110011	Chr17:68044222	20934210	2	CA10	Intron 5	Carbonic Anhydrase
Chr17:65036914	Chr17:47505787	17531126	2	MAP2K6/CA10	Intron 10/Intron 2	Kinase Signaling to p38/Carbonic Anhydrase
Chr17:47696961	Chr17:63830958	16133996	2	ARSG	Intron 2	Arylsulfatase G
Chr19:38085640	Chr19:38091701	6060	1	CCDC123	Part of intron 14 deleted	Coiled-coil domain Containing 123
Chr21:18540069	Chr21:33670572	15130502	2	CHODL	Intron 1	Chondrolectin
Chr21:20864799	Chr21:18281613	2583185	4	None	NA	NA
Chr21:19693189	Chr21:31062421	11369231	2	None	NA	NA
Chr21:21167077	Chr21:27861346	6694268	1	None	NA	NA
**Interchromosomal**						
Chr9:31759669	Chr11:84923849		1	DLG2	Intron 3	Disc Large Homologue 2
Chr10:117768069	Chr17:48272943		1	None	NA	NA
Chr10:121001050	Chr17:21238007		2	GRK5/KCNJ12	Intron 1/Intron 1	G-Protein Coupled Receptor/Potassium Channel
Chr15:23876380	Chr6:67079455		1	None	NA	NA
Chr17:61459185	Chr21:21169765		1	CCDC46	Intron 12	Coiled-coil domain containing 46
Chr21:35609289	Chr17:58726449		5	TANC2	Intron 7	Tetratricopeptide repeat, ankyrin repeat and coiled-coil containing 2

The *DPP10* gene was selected for further examination at the transcriptome level in the additional 53 samples by using reverse transcriptase-PCR (RT-PCR) assays. The presence/absence of the *DPP10* transcript was determined by targeting exon 2 and the exon 2–3 junction. *DPP10* transcript was detected in 31 of the 53 samples (55%). All of the 26 constituent exons were further analyzed by RT-PCR in the 31 samples expressing *DPP10*. In 7 of these samples, truncated *DPP10* transcripts were identified, from which we were either unable to amplify the region extending from exon 26 to the 3′ UTR (3 MPM samples), or found even shorter transcripts (4 MPM samples). Additional analyses of these transcripts are in progress.

Next, we investigated the correlation of *DPP10* expression with patient survival in the 53 samples tested. Surprisingly, we found that patients with tumors expressing any *DPP10* transcripts had statistically significant better overall survival compared with patients whose tumors lacked any *DPP10* expression (22 months versus 8 months median survival; *P* = 0.004).

The Illumina data were also analyzed for rearrangements, translocations, deletions, and inversions that were smaller than 1,000 bases and larger than 1 base. At a depth of at least 4 reads, 845 such rearrangements were identified of which 775 were common to the tumor and normal gDNA, 19 were unique to the normal, and 51 were unique to the tumor DNA representing additional mutations.

### Amplified rearrangements

Read densities from normal and tumor samples were compared and 14 regions exhibiting rearrangements stood out as being amplified in the tumor relative to the normal DNA by greater than 5X based on copy numbers. Of interest, over half of these regions (9/14, 64%) included genes that are of known significance in cancer such as the *DHFR* gene, whose product may confer methotrexate resistance, and the *PCBD2* gene, which is a co-factor for *TCF1* [39], [40].

### Single nucleotide variant (SNV) and indel variant identification

To initiate the search for disease-associated variants, including candidate somatic point mutations, SNVs were identified in tumor and normal samples and characterized as known SNPs (i.e., present in dbSNP) or novel SNVs which would represent either previously uncharacterized SNPs or mutations. With increasing coverage, the percentage of known SNPs among the SNVs rose to ∼95% suggesting that 4 to 5 reads may be sufficient for accurate SNV determination with this technology and depth of coverage (Table 3). Of interest, all four point mutations due to loss of heterozygosity (LOH) discovered previously in the tumor transcriptome of this patient [26] were also found to be present in the tumor gDNA in the current study providing additional validation of the approach. These mutations occurred in the coding regions of *AVEN*, *C9orf86*, *CTGLF6*, and *PSMD88P1/NOB1*. These observations, coupled with the fact that more than 90% of the SNVs discovered here are present in dbSNP, support the use of these sequencing data to identify heretofore unknown single base alterations.

**Table 3:** SNV Analysis.

	Illumina Tumor*			Illumina Normal*			454 Tumor*		
Supporting Read(s)	No of SNVs	Known SNPs		No of SNVs	Known SNPs		No of SNVs	Known SNPs	
		Count	%		Count	%		Count	%
= 2 reads	599,783	508,936	84.85	624,928	548,185	87.72	929,217	629,043	67.70
= 3 reads	347,027	317,641	91.53	330,330	305,757	92.56	463,075	355,305	76.73
= 4 reads	198,605	185,383	93.34	164,700	154,985	94.10	243,543	189,916	77.98
= 5 reads	109,543	103,455	94.44	76,707	72,825	94.94	126,668	96,046	75.82
= 6 reads	57,509	54,671	95.07	34,417	32,772	95.22	66,129	47,581	71.95
= 7 reads	29,960	28,588	95.42	14,758	13,947	94.50	36,329	23,620	65.02

* The depth is slightly different for each sequencing column accounting for differences in total SNVs.

### Candidate point mutation and indel identification by combining sequencing technologies

The Roche/454 and Illumina SNV datasets were merged to leverage the combined depth for prioritization of candidate point mutations in coding sequences. There were 200,226 SNVs present in both and absent from the Illumina normal sequence. Of these, 32,177 were new to dbSNP, 11,061 (33.4%) were present in known genes, and 90 (0.81%) were within known exons of which 44 were non-synonymous. These 44 candidate mutations, as well as 45 others present only in the Illumina tumor sequence at ≥2 coverage, new to dbSNP and within known genes, were independently sequenced (Sanger) in the tumor and normal samples. Of these, 83 were heterozygous novel SNPS, 3 were homozygous in the tumor only, presumably due to LOH, and 3 in *NKX6-2*, *CDH8*, and *NFRKB* were heterozygous point mutations. The *NKX6-2* and *NFRKB* mutations were non-synonymous and within coding exons. The *CDH8* mutation was within an intron.

Similar patterns of variant prevalence were observed in the analysis of single base indels. Illumina sequencing discovered 37,227 and 32,660 deletions in the tumor and normal specimens, respectively, at a depth of 2 or more reads. Of these, only ∼45% were previously registered as known indels in dbSNP. Likewise, 36,326 and 31,116 single base insertions were observed in the tumor and normal specimens, respectively, at a depth of 2 or more reads, of which approximately 47% were previously known. When combined at a coverage of ≥4 reads, there were 9,203 insertions and 8,444 deletions common to both the tumor and normal sequences of which ∼53% were previously known. Of the 9,203 insertions, 3,795 occurred within genes and 5 occurred within exons and could have functional consequences. Likewise, of the 8,444 deletions, 5,676 occurred within genes and 6 occurred within exons. Similar results were observed in the Roche/454 data. Taken together, the results from indel analyses indicate that the number of alterations of this type in the genome is substantially smaller than that of single nucleotide alterations consistent with previous reports [29].

### Expression patterns of disrupted genes in additional MPM specimens

In a follow-on analysis, we examined disrupted genes (n = 15, Table 2) and genes containing coding sequence point mutations (*NKX6-2*, *NFRKB*) in previously published gene expression microarray data from a total of 40 MPM patients [41]. Eleven of these 17 genes were represented on the microarray: *CA10*, *CCDC46*, *CHODL*, *DLG2*, *GRK5*, *KCNJ12*, *MAP2K6*, *MTAP*, *NFRKB*, *PRIM2*, *TANC2*. Five of these 11 genes (*CA10*, *CHODL*, *DLG2*, *KCNJ12*, *TANC2*) were not reliably detected (i.e., called “Absent” in all tumors). Five of the remaining 6 genes were detected in a variable number of MPM tumors ranging from 45–78% (gene, probe set, percent of samples called “Present”): *CCDC46*, 213644_at, 60%; *GRK5*, 204396_s_at, 55%; *MAP2K6*, 205698_s_at, 45%; *NFRKB*, 206968_s_at, 78%; *PRIM2*, 205628_at, 50%. The final gene (*MTAP*) was of particular interest since it was represented by multiple probe sets which were associated with discordant observations. *MTAP* was detected in 25% of MPM tumors using a probe set (204956_at) targeting the 3′-UTR (GenBank Accession #NM_002451.3). Interestingly, *MTAP* was also detected in 95% of MPM tumors using a probe set (211364_at) specific to an alternatively spliced transcript (GenBank Accession #AF109294.1) containing the first 4 exons of *MTAP* (of 8 total coding region exons) joined to an additional 2 exons located ∼25 Kb centromeric of *CDKN2B*. To be determined is whether the disruptions observed in these three genes in a single patient in the current study can be linked to differential expression patterns in additional MPM tumors.

## Discussion

We leveraged a combination of two high-throughput next-generation sequencing technologies to define the alterations that occur at the nucleotide level in a single MPM tumor. The copy number variations, the SNV profiles, as well as the many rearrangements observed indicate that the number of alterations in this single tumor is quite substantial both in terms of polymorphism of the normal genome and in tumor-specific mutations. At the depth sequenced here, many more rearrangement type mutations than point mutations were identified and validated. This finding, in combination with a previously known propensity for aneuploidy in MPM, is consistent with the hypothesis that MPM is more likely to be associated with larger-scale chromosomal rearrangements than point mutations. This might explain why specific oncogenes or tumor suppressors have not yet been found for MPM. Obvious caveats are that this pilot work focused on tumor from a single patient and that the depth of sequencing may possibly be biased towards discovery of rearrangement-type mutations. However, data from other re-sequencing projects [42] as well as from our transcriptome sequencing project [26] support the notion that some solid tumors have fewer point mutations than others. Also consistent with previous work is the observation that all four point mutations previously discovered in the transcriptome of this tumor [26] were due to a novel SNP present in one allele and a deletion in the complementary allele.

Rearrangements, translocations, deletions, insertions, and inversions have been associated with other cancers, particularly leukemias and sarcomas. Specific rearrangements have been clinically used for diagnosis and even to direct therapy as in the case of the BCR-ABL fusion protein, the target of the drug Gleevec [43], [44]. Given that most of the rearrangements we discovered involve chromosomes 8, 17, and 21, it is intriguing to speculate that a similar phenomenon may occur in MPM. At the very least, it highlights these areas of the genome as important for additional study.

Rearrangements have been implicated in many other solid cancers as well [35], [43]–[46]. Thus, the potential roles of rearrangements as oncogenic driver mutations and therapeutic targets in the solid tumor genome may approach that of somatic point mutations, at least for some tumors. However, unlike point mutations, rearrangements have been difficult to define precisely at the nucleotide level or to compare across many samples prior to the advent of massively parallel sequencing approaches. Therefore, the observations and the discovery methods developed herein support a role and provide a strategy for direct unbiased genomic sequencing for the precise identification, prioritization, and validation of rearrangements in solid tumors.

Limitations associated with individual massively parallel sequencing technologies include sensitivity versus specificity, the creation of artifacts, as well as the biases exhibited in certain stretches. It has been previously reported that each of the technologies used here has a large number of false positive artifacts suggesting that stand alone methods may not be sufficiently accurate to use independently for this purpose [18], [19], [27], [38]. Some biases that have been observed include the propensity for errors in homopolymer stretches, under representation bias against A/T rich sequence regions, and creation of rearrangement artifacts due to false primary priming during PCR expansion [18], [19], [27]. While greater depth of sequencing and innovative filtering techniques may improve upon some of the limitations encountered in this analysis, artifacts inherent in the method of sequencing will not be ameliorated with deeper sequencing. Therefore, the most powerful rationale for ruling in alterations comes from a comparison and agreement of at least two orthologous technologies. The need for the combination of two different next-generation sequencing technologies is further supported by the recent findings of Pleasance et al. who reported deep sequencing of a small cell lung cancer cell line genome [47]. These investigators identified 134 coding exon somatic mutations from among 22,910 somatic substitutions as well as 58 genomic rearrangements. At this number of putative mutations, rapid validation of all true mutations using traditional Sanger sequencing is not practical.

Although, clearly, more patient samples are needed to identify the most frequent genomic alterations that play a functional role in MPM, one may examine the data, postulate mechanisms of action, and make inferences relative to putative pathways involved in disease etiology. For example, many chemotherapeutic drugs are largely ineffective in the treatment of MPM, including methotrexate which targets the *DHFR* gene product [48]. The fact that there is a substantial amplification of the *DHFR* locus in the tumor of this chemotherapy naïve patient may explain why 80% of MPM patients are resistant to methotrexate and similar drugs [4], [6]. The 30 validated tumor specific rearrangements were directly within 17 genes and were just upstream from the promoters of 35 other genes (see File S2), most of which have not been previously described in cancer. However, several could plausibly be involved in oncogenesis as they are known to affect receptor signaling, signal transduction, cell proliferation, and apoptosis. Candidate genes for further study include a growth factor (*NRG3*), two membrane receptors (*EPHA6* and *GRK5*), two signaling molecules (*MAP2K6* and *NOS2*), a putative transcription factor (*TANC2*), as well as a potassium channel protein and modulator (*KCNJ12* and *DPP10*).

Particularly intriguing is the discovery that the absence of *DPP10* expression in MPM is associated with poor clinical outcome, i.e., survival. *DPP10* is located on the long arm of chromosome 2 (2q12.3–2q14.2), close to *DPP4* and FAP, and it extends over 1 Mb of genomic DNA. In *DPP10*, the serine residue critical to the active site of other DPP (dipeptidyl peptidase) family members is replaced by a glycine residue, such that *DPP10* lacks dipeptidyl-peptidase activity [49], [50]. *DPP10* binds specific voltage-gated potassium (K+) channels and alters their expression and biophysical properties [51]. Alternate transcriptional *DPP10* splice variants, encoding 2 different isoforms (i.e., the “long” and “short” isoforms), have been partially characterized within NCBI's RefSeq database. In all the MPM samples expressing *DPP10*, only the short isoform has been detected (data not shown). Northern Blot analysis revealed strong *DPP10* expression in brain, pancreas, spinal cord, and adrenal glands. Less expression was found in placenta, liver, and airways (trachea). Although Northern Blot analysis did not show *DPP10* expression, RT-PCR detected low *DPP10* expression in lung [49], [50].

It is unknown what functional role *DPP10* might play in MPM tumor cells, but if it modulates K+ channel function it could conceivably influence tumor cell survival or growth, as K+ channels are known in general to play important roles in regulating cell proliferation, cell cycle progression, and apoptosis [49], [50]. *DPP10* has also been linked to asthma by several association studies of linkage and fine mapping [52]. In addition, *DPP10* is part of the “DPP-IV activity and/or structure homologues” (DASH) molecules, which have deregulated expression in multiple human cancers determining their pathobiological relevance in carcinogenesis.

Given the variable expression patterns, known and inferred physiological functions, and likely role of *MAP2K6*, *CCD46*, and *MTAP* in tumor cells, it is a priority to determine whether the differential microarray gene expression patterns that were observed in a larger number (n = 40) of additional MPM samples can be replicated in additional samples and linked to any of the genomic disruptions observed in the current study. Specifically, we are testing the hypothesis that MPM samples lacking expression of these genes harbor similar genomic disruptions. *MAP2K6* is a kinase that phosphorylates p38 leading to apoptosis. Hence, loss of expression (i.e., function) would inhibit a cell death signaling mechanism. Given the level of aneuploidy in the MPM tumor, a mutation resulting in a Chr17:21 translocation that interrupted *CCDC46* was notable. This gene product is likely involved in DNA replication/repair based on conserved protein domain analysis [53], [54], and a lack of expression would likely promote tumorigenesis. In addition, mutations in this family of genes are known to be associated with gross chromosomal aberrations such as those evident in many MPM tumors, including the sample analyzed in this study. This mutation could potentially represent a driver mutation for the tumor given the incidence of rearrangement mutations [44]. Finally, the function and physiological role (if any) of *MTAP* alternatively spliced transcript is unknown, but it is frequently deleted in many types of cancer [55] making its presence in 95% of MPMs (i.e., 38/40 tumors) an intriguing avenue for additional exploration.

In addition to rearrangements, we discovered three novel point mutations in three different genes (*NKX6-2*, *CDH8*, *NFRKB*). *NKX6-2* is a transcription factor with known positive and negative regulatory activities in development and differentiation [56] and has been postulated to be a tumor suppressor for some types of brain tumors (e.g., oligodendrogliomas) [57]. *CDH8* codes for cadherin, part of an integral membrane protein family affecting calcium-dependent cell adhesion. Inappropriate *CDH8* expression has been linked to a subset of renal cell carcinomas [58]. *NFRKB* codes for a DNA-binding protein that interacts with *NFKB*1 and is a candidate oncogene currently being evaluated in hematologic and solid tumors [59], [60].

This analysis demonstrates that there are many major, tumor-specific chromosomal rearrangements in this single MPM tumor and that clues discovered in the sequence of one tumor can lead to the discovery of genes that might be inactivated in other ways in related tumors. Ultimately, functional analysis, deeper sequencing, and genotyping in additional specimens will be required to identify true driver mutations in MPM and better define specific targets for therapy. We find that comparison of tumor to normal sequences for each case and using a second sequencing strategy, at least at the validation phase, are essential given the large number of polymorphisms and sequencing artifacts observed in MPM. Even now, the cost of sequencing and bioinformatics analysis such as described herein is too costly for widespread clinical application. However, third generation sequencing technologies are becoming available at more affordable prices. We envision that within the next few years the high-throughput analysis of multiple cancer genomes will become a reality and ultimately part of the clinical diagnostic pipeline.

## Methods

(Also see Materials and Methods S1, Materials and Methods S2, and Table S2.)

### Tumor Specimen and Sequencing

High quality, tumor enriched (>83%) gDNA was prepared from frozen tissue tumor sections of a mixed histology MPM tumor resected from a male patient using microaliquoting methodology [61]. Non-tumor normal gDNA was prepared from lung of the same individual. (Additional tumor specimens used for validation purposes were obtained from the tumor bank at our institution.) All human tissues were acquired under informed written consent from a patient undergoing definitive surgery for mesothelioma at Brigham and Women's Hospital in Boston, MA. All work was performed with protocol permission and under the guidelines of the Institutional Review Boards at Brigham and Women's Hospital and the Dana-Farber Cancer Institute. Sequencing data were deposited at GeneBank: GenomeProject ID #41515.

For Illumina sequencing, gDNA was sequenced using the Illumina Genome Analyzer 2 (GA2). PE reads of base pair (bp) lengths 50 bp (4.6 Gbp), 45 bp (4.4 Gbp), and 36 bp (8.8 Gbp) were generated from 5 runs on GA2 which produced 17.8 Gbp of tumor genomic sequences. A further 4 runs of normal gDNA of the same patient were used to generate reads of 50 bp (10.6 Gbp) and 36 bp (5 Gbp), totaling 15.67 Gbp of genomic sequence data [19].

For Roche/454 sequencing, tumor gDNA was also sequenced using the Roche/454 GS20 Sequencing™ System (Roche Applied Sciences, Indianapolis, IN) to generate 47,359,608 sequence reads with an average length of 228 bp, comprising a total of 10.8 Gbp [19].

### Sequence filtering criteria and mapping

All PE reads from the Illumina and reads from the Roche/454 were filtered using the criteria of average read quality score (QS) ≥20. Duplicate identical reads were discarded. PE reads that passed filtering criteria were mapped individually to RefSeq Human Genome V36.3 using Synamatix's SynaAlign program with a mapping threshold of −30 and mapping margin 0, which allowed up to 3 mismatches and up to 2 opening gaps. A maximum of 10 alignments were reported for each hit and the Percentage Identity (PID) filter was set at ≥94%. The sample-specific mapping results were paired using Read ID tags. Reads belonging to the same PE shared the same unique read ID. From these pairings, mapping results were segregated into four distinct groups. These groups were labeled as: 1∶1 where both reads mapped uniquely against the human genome; 1:N where the first read mapped uniquely while the second read either hit nil or multiple sequences (hence inclusive of 1∶0); N:1 where the second read mapped uniquely while the first read either hit nil or multiple sequences (hence inclusive of 0∶1); and N:N where both reads mapped to multiple sequences. The unique reads were defined as all pairs of reads belonging to the groups of 1;1, 0∶1, 1∶0. The main aim of this round of mapping was to further filter out potential repeats from the unique reads by using a more stringent set of parameters: Mapping threshold −27, Margin 0, Gap open 12, Gap extent 5 with mismatch penalty 10, Max number of N:12, and Maximum alignments per read 10. The pairing process was repeated for a second round and the mapping results were segregated into the same four groups as described above. Reads that passed the second stage mapping were compiled into ‘RowFormat’ files and used for the detection of rearrangement and SNVs, and for read density analysis. Alignment of Roche/454 DNA read sequences to an unmasked Human reference genome (Build 36.3) used SynaSearch (Synamatix), a BLAST-like alignment tool, with a seed size of 15 base pairs. The seeding process excludes seeds over-represented in the reference genome by a factor of 6.0 or more and also requires multiple nearby seeds to align before triggering detailed alignment with a dynamic programming algorithm.

### Comparative genomic hybridization (CGH)

CGH was performed comparing the tumor and normal control specimens using the Nimblegen (Roche/Nimblegen Systems, Madison, WI) Homo Sapien 8 array Whole Genome Tiling set. This was HG18 build, NCBI 36, with probe length of 50–75 and median probe spacing of 713 bp. The informatic analysis was performed by Nimblegen using the segment algorithm to determine changes in copy number [62].

The Pearson product-moment correlation coefficient was used to analyze the strength and direction of the linear relationship between CGH and PE tumor densities. A strong correlation coefficient (between 0.7–1.0) denotes a strong linear relationship between both CGH and sequence read density whereby duplications and deletions are common in both. A positive coefficient indicates that the increasing and decreasing trends corresponded in both CGH and sequence read density, and vice versa. The analysis was computed for all chromosomes as a whole. The 99% confidence interval denotes the probability that a Pearson correlation coefficient would fall out of the interval is 1%.

### Copy number and read density analysis

Read densities for each of the Illumina tumor and normal sequences were calculated from the number of accumulated reads in a bin. Mapping hits were treated both as single ends: 1:N, N:1, N:N and as paired-ends: 1∶1, 1:N, N:1, N:N. Similarly for 454 sequence, read density was calculated from the accumulated reads in a bin. Since 454 reads were not paired-end, mapping hits were used in the calculation. A bin size of 1000 bp was used in the calculation, and display of read density. The hit probability for a repeat hit was calculated before it was added to the respective read density bin. Read density charts based on the above criteria were compiled for both normal (Illumina only) and tumor (both Illumina and 454) reads, respectively. They were plotted as read density with respect to chromosomal location and according to individual chromosomes. From the read density charts, abrupt changes (also termed ‘edges’) of read density can easily be visualized and thus detected. The main aim was to detect copy number variations (CNVs) in the read density profiles and to compare and contrast these findings between the samples and sequencing platforms. To identify rearrangements and translocations, CNVs along the length of each chromosome were analyzed, by using the read density charts for the tumor sample, and compared to the CGH plots, to the normal gDNA sequences, and to the reference genomes for each chromosome.

### SNV detection

Only unique filtered high quality PE reads where both ends fulfilled the following conditions were retained for SNV analysis: average QS of ≥20; and a PID ≥94. Three types of SNVs were detected: single base substitution and insertion or deletion of a single base, hereafter referred to as SNPs or indels. Any SNVs detected at query positions 1 or ≥31 of the read were discarded because of the high probability of base-calling errors at these positions. A subsequent filtering was performed using the best QS and supporting reads of a SNV. The rule used was SNVs with a BQS <23 and fewer than two supporting reads were discarded. Unique hits of all 454 reads and top hits of 454 reads with multiple alignments were channeled for SNV detection. These alignments were further filtered and only alignments with ≥80% mapped length, ≥20 average read QS, and ≥90% identity were processed by the SNV pipeline. Again, three types of SNVs were detected from these 454 tumor reads, i.e., SNVs and indels. All identified SNVs were cross-checked against NCBI dbSNP (version number B129).

### Detection of chromosomal rearrangements

Illumina: 1∶1, 1:N and N:1 PE reads that hit a repeat at one side were used for rearrangement detection only if the next best hit had a difference of ≥2 bases with best hit. In cases of multiple hits, only the first and second best hits were compared. Rearrangement hits obtained were further filtered to have an average QS of ≥20 and a hit PID of ≥94%. Putative rearrangements were ranked according to the number of supporting reads and whether they were located near a read density change.

Roche/454: Read sequences in which a single read contained two distinct and contiguous sequences that mapped to different locations in the human genome were selected. If the best alignment to the read failed to align to 35 bp or more from the 3′ end of the read or 45 bp or more from the 5′ end of the read, then the unaligned portion of the read was subject to a second, more sensitive round of alignment against the Human reference genome using SynaSearch with a seed length of 14 bp. If there was a unique alignment to this portion of the read then the original alignment and the new alignment formed a rearrangement pair. The rearrangement pairs were then combined to find rearrangement locations supported by more than one read. Many reads were found near centromere and telomere repeats, especially when the centromere sequence was not in the reference genome. These were assumed to be the result of a centromeric sequence not in the reference genome being aligned to a similar sequence that is in the reference genome. To filter these we removed pairs which had excessive reads at one or both sides (>40 reads) and where one side of the rearrangement pair had 3 or more alternate paths or graph edges leading from it.

### Validation studies

Selected candidate mutations were further characterized using PCR and conventional Sanger sequencing of PCR products from tumor and blood gDNA. The PCR analysis for validation was performed as described previously [26]. Analysis of *DPP10* expression was conducted using standard RT-PCR and exon-specific primers. To determine the presence or absence of *DPP10* transcript, RT-PCR was performed targeting exon 2. The presence of the transcript was confirmed using primers spanning the exon 2–3 junction. The 31 MPM samples showing *DPP10* expression were further analyzed using primers that amplify exons 3–8, 4–9, 5–10, 9–15, 13–19, 17–22, 20–26, and 20- 5′UTR. In 7 samples, the amplicons were not detected in some of the PCR reaction targeting different regions of the 5′-DPP10 transcript. These truncated transcripts were further analyzed using additional primers. The primers are listed in Table S2. Survival analysis of patients with *DPP10*-expressing tumors was conducted using Kaplan-Meier survival curves and the log-rank test for determination of statistically significant differences (*P*<0.05). Affymetrix microarray gene expression data (U133A array) from a total of 40 MPM tumors [41] was obtained from NCBI (GEO Accession #GSE2549).

## Supporting Information

Materials and Methods S1 Expanded materials and methods.
(0.05 MB DOC)

Materials and Methods S2 File describing methods to analyze rearrangements.
(0.19 MB DOC)

Table S1 Chimera read sequences for tumor chromosomal mutations.
(0.07 MB DOC)

Table S2 PCR Primers used for rearrangement mutations validation.
(0.05 MB XLS)

File S1 (1.11 MB PPT)

File S2 Direction and chromosomal locations of the rearrangement.
(1.79 MB PPT)
