# Supplementary material for: Second Generation Sequencing of the Mesothelioma Tumor Genome
Source: PLoS One. 2010 May 13;5(5):e10612. doi: 10.1371/journal.pone.0010612 (PMC2869344; doi:10.1371/journal.pone.0010612)
Supplement: Materials and Methods S1 — Expanded materials and methods. (0.05 MB DOC) [file pone.0010612.s001.doc]

**Materials and Methods S1**

**Section 1 Sequencing of Patient Samples**

Sequencing of Normal and Tumor Genomic DNA:

High quality gDNA isolated from tumor and normal lung [s1] tissue was sequenced using the Illumina Genome Analyzer. Paired end (PE) reads of lengths 50bp (4.6Gbp), 45bp (4.4Gbp), and 36 bp (8.8Gbp) were generated from five runs on an Illumina GA 2 [s2]DNA sequencer which produced 17.8 Gbp of Tumor Genomic Sequences corresponding to 5.6 times the depth of coverage of the Human Tumor genome. A further four runs of Normal Genomic DNA from normal lung tissue of the same patient were used to generate reads of 50bp (10.6 Gbp) and 36bp (5Gb), totaling 15.67 Gbp of genomic sequence data which corresponded to 5.2 times the depth of coverage of the Normal Human Genome.

**Section 2 Filtering based on Read Analysis and Quality Score**

Illumina Base Filtering

All PE reads from the Illumina machine were filtered using the following criteria to improve the quality of data for the SNP pipeline:

* Average Read Quality Score

All reads were analyzed to determine the average read Quality Score (QS), which was calculated by computing the mean QS for all the bases of each read. Only reads with an average QS of >20 were used for downstream processing.

* Distribution of Base Quality Score

85% of all reads were found to have an average QS of >20. The remainder of the reads (15%) that were found to have an average QS of <20 were discarded from any further analysis for both the Tumor and Normal data sets.

* Duplicated Reads

Duplicate reads, defined as ‘reads with the same start and end position in the same sequencing lane’, were found and removed prior to further processing. An average of 3-5% of all reads was found to be duplicates in each lane.

* Gap Analysis for Paired End Reads

We found that in both the normal and tumor genomic DNA, the majority of PE reads had a mean gap of 55bp.

* Read Length

From our analysis only bases from positions 2 to 31 were of sufficient quality to be used in the SNP detection analysis pipeline by setting a Base Quality Score (BQS) threshold of ≥23.

454 Base Filtering

All reads from the 454 machine were filtered using the following criteria to improve the quality of data for the SNP pipeline:

* Average 454 Read QS

All reads were analyzed to determine the average QS which was calculated by computing the average QS for all bases of each read. Only reads with an average QS of ≥20 were used for the SNP pipeline and BQS threshold of ≥23.

* Duplicate 454 Reads

Reads that were exact duplicates were removed prior to further processing to avoid the calling of false positives.

**Section 3 Mapping of Illumina and 454 Reads**

Illumina PE reads were mapped to a reference genome in order to obtain high quality alignments. Mapping was performed using a two-stage process so as to eliminate any alignments in repeat regions:

Stage 1: First Round of Mapping of Illumina PE Reads

Illumina PE reads that passed filtering criteria were passed forward for stage 1 mapping. They were mapped individually against RefSeq Human Genome V36.3 using Synamatix’s SynaAlign program with the following parameters: Mapping threshold -30, Margin 0, Gap open 12, Gap extent 5 with mismatch penalty 10, Max number of N:6, and Maximum alignment per read 10. The aforementioned parameters constituted a Percentage Identity (PID) of 94%.

The sample-specific mapping results (for individual files) were paired using Read ID tags. Reads belonging to the same PE (reaction) shared the same unique read ID. From these pairings, mapping results were segregated into four distinct groups. These groups were labelled as: 1:1 where both reads mapped uniquely against the human genome; 1:N where the first read mapped uniquely while the second read either hit nil or multiple sequences (hence inclusive of 1:0); N:1 where the second read mapped uniquely while the first read either hit nil or multiple sequences (hence inclusive of 0:1); and N:N where both reads mapped to multiple sequences.

Stage 2: Second Round of Mapping of Illumina PE Reads

The second stage of mapping was performed on all unique reads. The unique reads were defined as ‘all pairs of reads belonging to the groups of 1;1, 0:1, 1:0 as described in Stage 1’. The main aim of this round of mapping was to further filter out potential repeats from the unique reads by using a more stringent set of parameters: Mapping threshold -27, Margin 0, Gap open 12, Gap extent 5 with mismatch penalty 10, Max number of N:12, and Maximum alignment per read 10.

After Stage 2 mapping, the pairing process was performed again and the mapping results were segregated into same four groups as described in Stage 1: 1:1, 1:N, N:1, and N:N.

Reads that passed Stage 2 mapping were compiled into ‘RowFormat’ files and used for the detection of chimeras and Single Nucleotide Variations (SNVs), and for read density analysis which will be discussed in detail in later sections.

Mapping Percentage and Duplicate Percentage of Illumina Reads from Stage 1 and 2

From our analysis, 94% of all Reads from Stage 1 mapped uniquely to the Human reference Genome while 5% were found to be duplicates and discarded. Of the reads found in Stage 2, 99% mapped uniquely to the human genome while less than 1% were discarded.

**Section 4 Read Density Analysis**

Data Used to Calculate Read Density

Read density for each of the tumor and normal samples was calculated from the accumulated reads and is defined as ‘the number of accumulated reads in a bin’. As in Stage 1 mapping hits were treated as single ends from Stage 1 mapping: 1:N, N:1, N:N; and from Stage 2 mapping: 1:1, 1:N, N:1, N:N.

Bin Size for Read Density Calculations

A bin size of 1000bp was used in the calculation, and display of read density. The hit probability for a repeat hit was calculated before it was added to the respective read density bin. Read density charts based on the above criteria were compiled for both Normal and Tumor reads respectively, and they were plotted as read density with respect to chromosomal location, and according to individual chromosomes.

Using Read Density Analysis to Detect Copy Number Variations

From the read density charts, abrupt changes (also termed ‘edges’) of read density can easily be visualized and thus detected. The main aim was to detect copy number variations (CNVs) in the Illumina Tumor, Illumina Normal and 454 Tumor read density profiles in order to compare and contrast these findings between the samples and sequencing platforms.

Comparing Read Density Charts of Different Platforms

Each genome (Illumina Tumor, Illumina Normal and 454 Tumor) was partitioned into 1kbp windows, and the number of reads that mapped onto each window was quantified to reflect chromosome copy number. A number of chromosomes in tumor genomes (Illumina and 454) appeared to have both haploid and polyploidy segments, such as 1, 8, 9, 11, 15, 16, 17, 21, and 22. Furthermore, chromosomes Y, 4, 14, 18, 19 and 10 appeared to have fewer copies while chromosome 5 appeared to have extra copies. Chromosomes 8, 10, 17, 19, and 21 were selected as key chromosomes for further analysis.

To examine structural mutations, copy number variations along the length of each chromosome were analyzed using the read density charts for tumor samples, and compared to the comparative genomic hybridization (CGH) plots for several chromosomes. The CGH results of the tumor samples largely confirmed the sequence analysis across all chromosomes; shows the results for chromosome 21 (the read density charts for all key chromosomes are provided in supplementary materials) where numerous transitions in read density exist in the tumor and correspond with changes in CGH intensity, thus highlighting the capability of these methods to detect complex intrachromosomal abnormalities. As displayed in the same figure, the parallel analysis of tumor (Illumina and 454) and Illumina normal samples shows that the vast majority of chromosome copy number variations observed via sequence analysis were present in tumor tissue, but not in normal tissue, from the same individual, hence indicating mutations.

**Section 5 Detecting the Edges of Regions of Copy Number Variation**

Algorithm for Detection of Edges

Synamatix has developed an efficient algorithm to detect edges, which are classified as ‘locations with abrupt changes in read density, which mark the ends of a region with significant copy number variations. For each chromosome, read densities (using a bin size of 1000bp) were initially filtered to remove outliers located far away from the mean of read densities. Subsequently, using a sliding window approach, a T-score for each chromosomal position was computed based on a pre-defined window size. For each chromosomal position, its corresponding T-score measures the discrepancy between the distribution of read densities in the left window to its counterpart in the right window. In the next stage of the detection algorithm, a threshold based on the T-score was defined such that if the T-score of regions of single chromosomal positions surpassed this threshold, they were carried onto the next stage.

Overall, the edge detection algorithm generated three types of edges. The first type being single chromosomal locations where T-scores exceeded the threshold. The second type being chromosomal locations where T-scores exceeded the threshold but neighboring chromosomal locations of the peak within the same region all have T-scores which were <70% of the peak T-score. The third type being a where T-scores exceeded the threshold and neighboring chromosomal locations of the peak within the same region all have a T-score >=70% of the peak T-score. For the latter case, an edge is associated with a width which denotes the neighboring locations of peak, where the T-score >=70% of the peak T-score.

For the first type, of all the edges where the T-scores exceeded the threshold, the corresponding chromosomal locations (an edge is a chromosomal location) were directly categorised as significant edges. For the second type, the peak locations were categorised as significant edges. For the final type, the aforementioned neighboring locations were taken to delineate the width of edges, and were generated together with corresponding peak positions as significant edges.

Filtering of Edges

Any edges that were within 500Mbp of the centromere were not further assessed. Using different window sizes and T-score thresholds would have led to different numbers of significant edges. Thus, the detection algorithm was extended by identifying edges with high statistical significance. First, the window size and T-score threshold selected were 15kb and 1.5 respectively. The initial number of edges found was high (using a 15kb window and 1.5 T-test[s3] threshold). Subsequently for each chromosome, the read density data was divided according to the positions of these edges whereby a segment was composed of a left window and a right window for each edge. An additional T-test [s4]calculated the observed ratio of the left side mean to the right side mean of all edges. Finally, the expected mean ratio was computed using random read densities in the same segment, and the same process was re-run for 10,000 iterations. For each edge, if the total number of iterations having an expected mean ratio < an observed mean ratio was greater than 9900, then this edge was considered a 99% statistically-significant edge.

Refinement of Edges to Detect Sample Specific Structural Variations

As changes in CNVs could be associated with structural variations, the edges that were identified were added to the Chimera detection pipeline. To better define candidate mutations, a series of empirically derived rules were developed and applied to select for true tumor specific chimeras. These rules required that a tumor chimera satisfies the following criteria; 1) is absent from normal lung sample and, 2) is located near an edge where a read density change occurred on both sides of the chimera. Chimeras that had one or both sides in a repeating element such as a centromere were also excluded and, 3) where the corresponding edge in tumor read density is absent from the normal lung sample and, 4) have a read support of at least 2. We used edges that are 99% statistically significant with the aim of reducing edges that are false positives or artefacts.

In addition, further analysis was carried out on common chimeras between samples. To define common candidate mutations, common edges were defined as edges which appeared in two samples based on the condition that either their corresponded chromosomal locations were equal. Using a less stringent condition, edges which chromosomal locations that are in close proximity (such as +/-5kb) to each other were considered as well.

Detection of 454 Tumor Edges

We also performed similar detection, filtering, and refinement steps for edges that were detected when using 454 Tumor sample reads.

**Section 6 Detection of Chimeras**

Mapping and Clustering of PE reads to detect Putative Chimeras

From the second round of mapping (Stage 2, section 2), only the following groups of mapped PE reads were compiled and analyzed for the detection of chimeras: 1:1 (from Stage 2), 1:N and N:1 (from Stage 2). PE reads that hit a repeat at one side were used for chimera detection only if the next best hit had a difference of ≥2 bases with best hit. In cases of multiple hits, only the first and second best hits were compared.

From this primary analysis, only putative chimeric hits were used for further investigation. Putative chimeric hits were defined as ‘PE reads that crossed chromosomes (i.e. the left and right reads mapped to different chromosomes), or PE reads that mapped to a single chromosome, where both ends of the reads, and the intervening mapped gap between the left and right reads, were >1000 bp in length.

Chimeric hits obtained using the above parameters were further filtered. Only high quality hits where both ends of a PE read fulfilled the following criteria were retained: Reads had to have an average QS of ≥20 and a hit PID of ≥94% (i.e., not more that two mismatches when mapped to refseq Human Genome).

Only filtered high quality chimeric hits were sorted by chromosome and genome offset and were annotated in the following format: Chromosome I, Offset I, Chromosome II, Offset II. Inter-chromosomal chimeric hits were sorted numerically on the basis of the chromosome number. Sorted hits were then further filtered and duplicates were removed. Duplicates were classified as ‘where more than one pair of reads has both left and right sides of the chimera hitting the same start and end sites.’

Clustering of Chimera and Reporting Nomenclature

Sorted and unique PE hits were then clustered using a sliding window size of 200bp and for each putative chimera on either side, the following details were documented:

Hdr1

Chromosome number of the left-end segment of the cluster

Start1

Chromosome offset of the left segment

Range1

Total bp of the left segment

Reads

Total supporting reads for the cluster

Hdr2

Chromosome number of the right-end segment of the cluster

Start2

Chromosome offset of the right segment

Range2

Total bp of the right segment

++ ,--, +-,-+

Number of supporting reads in the ++, --, +- and -+ orientation

ReadId

ID of all supporting reads

ReadsLoc

Location for each supporting reads – target offset

Table 1: Annotation of Chimeras

Putative chimeras were ranked by two criteria: the number of supporting reads and whether the identified chimera was located near a read density change. The list of mapped chimeras was compared to identify those that were common and unique to both normal and tumor samples.

Using these mapped chimeras, further analysis was carried out to identify possible interesting chimeras based on the following criteria: missing exons, that may have been present in the same-chromosome chimeras, and chimeras that resulted in gene fusions.

Illumina genomic DNA chimeras were compared with those that had been previously identified by 454 pyrosequencing using the MPM Transcriptome data set (#Sugarbaker et al).

Chimera Validation via Inter & Intra Sequencing Platform Support of Reads and Edges

Chimeras that were identified were presented as in a table and annotated with all the parameters, as shown in Table 1. The chimeras in the list were subsequently ranked on the basis of read support indicating detection by either Illumina and/or 454 sequencing methods. Additional tables containing results such as read density edge support from the same sequencing platform (e.g. Illumina chimera reads supported by Illumina edges, 454 Chimera reads supported by 454 edges, unique Illumina tumor chimera reads supported by unique Illumina tumor edges, and unique 454 tumor chimera reads supported by unique 454 tumor edges) were also compiled.

The rule applied in filtering Chimeras by edge support is straight forward where Chimeras with both sides in close proximity (such as +/-15kb) of a unique tumor edge were considered true tumor chimeras irrespective of read support. It has been demonstrated in previous studies that even with a read support level of 1, if a chimera has both sides supported by an edge, then such a chimera is considered statistically significant if the observed number of chimeras is larger than the expected number.

Annotation and Visualization of Chimeras

To easily visualize chimeras detected using 454 and Illumina sequencing, as well as other results such as edges by Illumina and 454; read density by Illumina Normal Reads, Illumina Tumor Reads, 454 Tumor Reads, CGH and regions of Loss of Heterozygosity (LOH), Synamatix utilized a radial plot method, namely circos radial plot, where all these criteria could be plotted and co-visualized with clarity in a figure, either for individual chromosome or inter-chromosomes. Two main types of radial plots were generated for key chromosomes. The first type displayed all Illumina chimeras without both sides supported by edges with a read support of ≥2, and all Illumina tumor chimeras with both sides supported by edges and a read support of ≥1. For larger key chromosomes, such as chromosomes 8 and 10, Illumina chimeras without edge support and a read support of ≥3 were plotted instead clarity. Included in the radial plots were Illumina and 454 unique tumor edges, Illumina read density (both normal and tumor) and 454 read density (both normal and tumor).

The second type of radial plots had the same features as the first plot, with the addition of 454 chimeras[s5] unique to tumors with edge support and a read support of ≥1 plotted in a separate compartment from the Illumina chimeras.

Classification and Annotation of Structural Variations

The chimera results were presented in a table containing all the necessary information about the read support type and mapped locations as well as the existence of any altered genes (e.g. exonic deletions or gene fusions) in the chromosomal rearrangement. Chimeras were then classified as ‘unique to tumor’ or ‘normal’ if they met the chimera-calling criteria and if there was a corresponding non-chimeric read support in the opposing sample. We called non-chimeric read support if all reads spanning 1kb either side of a chimeric read mapped to the reference genome (Human RefSeq Genome 36.3). Only chimeras that met these criteria were called ‘tumor specific’ (also termed ‘unique to tumor’ or ‘tumor unique’).

Detection of Amplified Translocations by Chimera with Corresponding Differential CNVs in Tumor and Normal Samples

As mentioned earlier in previous sections, chimeras that were detected were clustered on the basis of their chromosomal locations. During this procedure we noticed a subclass of chimeras, present in both normal and tumor genomic DNA, that also displayed differential CNVs between the tumor or normal samples. We investigated this further and were able to describe a class of amplified translocations where one or both segments of the chimeras were amplified over the corresponding chromosome locus in either the tumor or normal by a factor of 5 and above. These chimeras were chosen for further analysis and we studied the genes involved and their arrangement.

**Section 7 Detection of SNVs**

SNV detection

As for the chimera pipeline, only unique PE reads obtained from Stage 2 of the pipeline were further filtered, and only high quality reads where both ends of a PE read fulfilled the following conditions were retained for SNV analysis: average QS of ≥20; and a PID ≥94. For each sample, the high quality single end hits were sorted by chromosome and genome offset in the following format: Chromosome, Offset, and Variant. Three types of SNVs were detected by this SNP pipeline: a single base substitution, insertions of a single base and deletions of a single base, hereafter referred to as SNPs and Indels. Any SNVs detected at query positions 1 or >31 of the read were discarded due to the high probability of base calling errors at these positions. A subsequent filtering was performed using the BQS and supporting reads of a SNV. The rule used was SNVs with a BQS ≥23 and fewer than two supporting reads were discarded.

Reporting of Tumor Specific, Normal Specific and Common to Normal and Tumor SNV

All identified SNVs were cross-checked against NCBI dbSNP (version number B129). SNVs that had met our identification criteria of read support ≥2 and were not present in dbSNP were classified as novel SNVs. SNVs identified by the above criteria were then classified as ‘unique to tumor’, ‘unique to normal’ or ‘common to both tumor and normal’ based on the following criteria: unique to tumor had an SNV read support of ≥2 in tumor reads, and an SNV read support =0 and non-SNV read support of ≥2 in normal reads; unique to normal were SNVs with a read support of ≥2 in normal reads, and an SNV read support =0 and a non-SNV read support of ≥2 in the tumor reads.

Classification of SNVs

Unique tumor-specific SNVs were further analysed to determine if any of the SNPs mapped to genes were in identifiable promoter regions, or mapped to known exons. SNV that mapped to known transcripts with putative open reading frames were then analyzed to determine if they caused an alteration of the putative amino acid sequence or introduced a stop codon, hence being able to be classified as non synonymous or synonymous silent mutations. The classification of synonymous and non-synonymous SNVs was confirmed by mapping to SwissProt (version 56.6) to determine if SNVs were present in genes, to the EPD database for promoters (release 97), and to NCBI’s CCDS database (version CCDS 38.2) for exons. Each of these criteria, save the amino acid substitutions, was detected by taking the original SNV-containing reads and mapping them to the corresponding reference data set. To detect amino acid substitutions for non-synonymous SNVs, the original SNV read was taken and 80 bases of the original reference sequence, with 40bp from either side, were added onto it and the resultant sequence was mapped against SwissProt to detect alignments with amino acid substitutions.

Comparison of identified SNVs and Structural Variations to those found in the MPM tumor Transcriptome

Genomic SNVs and structural variations that were detected in this study were compared to those previously identified in the MPM transcriptome (Sugarbaker et al).

**Section 8 Comparison of Illumina and 454 results and Merging of Data to improve pipeline efficacy**

All results obtained from genomic sequence reads of both Illumina and 454 were compared and contrasted to determine the biases in each of the sequencing platforms. Results in this paper demonstrated that Illumina is superior in calling SNPs and indels compared to the 454 platform which has been reported to have problems in base calling due to homopolymer errors (see Discussion in manuscript). Our results also confirmed that both Illumina and 454 have a high percentage of false positives in calling chimeras which has recently been reported to be as high as 90% (see Discussion in manuscript) and which can be plausibly explained by the chemistry of sample preparation. Despite this shortfall, the 454 platform, with its longer reads, was more effective in identifying the break points of chimeras. Given the respective strengths and weaknesses of the Illumina and 454 platforms, we have demonstrated in this study that a combinatorial approach results in the increase in identification of true positives.

Abbreviations

QS: Quality Score

PE: Paired End

SNVs: Single Nucleotide Variations

CNVs: Copy Number Variations

PID: Percentage Identity

Average QS: Average Read Quality Score

BQS: Base Quality Score

[s1] normal lung tissue/ sample

[s2] GA II

[s3] T-score

[s4] T-score

[s5] unique tumor chimeras
