# Supplementary material for: Second Generation Sequencing of the Mesothelioma Tumor Genome
Source: PLoS One. 2010 May 13;5(5):e10612. doi: 10.1371/journal.pone.0010612 (PMC2869344; doi:10.1371/journal.pone.0010612)
Supplement: Materials and Methods S2 — File describing methods to analyze rearrangements. (0.19 MB DOC) [file pone.0010612.s002.doc]

# Materials and Methods S2

# Rearrangement Discovery Methods and Results Based on 454 and CGH

This supplement provides:

A description of the process to filter chimera read alignments.

A description of the four informatic methods used to identify chimeras in the sequence data.

Tables and figure showing the results of chimera discovery.

The rationale for using this strategy to discover chimeras.

## Description of chimera read filtering.

The sequence alignment process identified 209,976 chimera reads, which was reduced to a list of 155,524 chimera locations.

| **Step** | **Description** | **Chimera Reads Removed** |
| --- | --- | --- |
| 1 | Chimera reads with multiple high scoring alignments to one part of the chimera, i.e., ambiguous location. | 23,338 |
| 2 | Chimera reads with alignment showing less than 95% sequence identity at base level. Short inserts and deletes were allowed. | 5,717 |
| 3. | Duplicate reads removed. The sequencing process creates between 10 and 20% duplicate fragments which produce reads that differ only in sequencing errors. These were identified as having the same alignment location as an earlier read and then discounted when counting supported chimera locations. | 20,098 |
| 4. | Combine unique chimera reads to produce a list of 155,612 chimera locations, some supported by multiple reads. |  |
| 5. | Remove 7 locations covered by > 40 reads (includes CV, JW & transcriptome reads). For a tumor specific chimera and based on haploid genome the probability of this is 10^-38^. | 2619 |
| 6. | Remove 81 chimera locations with 3 or more alternate graph edges from either side. | 354 |
|  | Chimera Reads | 157,800 |
|  | Chimera locations identified | 155,524 |

## Description of the four methods used to identify chimeras in the sequence data.

Chimeras resulting from somatic chromosomal mutations had to be separated from germ line chimeras and chimeras created *in vitro* during the sequencing process. We proceeded to look for chimeras that were associated with copy number variations (CNVs). We analyzed the read density and comparative genomic hybridization (CGH) data to identify the locations of CNV changes, and then we looked for chimeras near the CNV. Details of the process are discussed in section 3 of this supplement. Four variants of the process were used to produce lists of chimeras for PCR validation.

### Read Density change at both alignment locations of the chimera.

Student’s t-test was used to detect a change in mean read density across the alignment location of each of the chimera parts. Read density was measured in 50Kbp windows and a t-test score of at least 4 was required.

### CGH Log ratio change at both alignment locations of the chimera.

Student’s t-test was used to detect a change in mean CGH log ratio across the alignment location of each of the chimera parts. Mean CGH score was measured using 100 probes and a t-test score of at least 3.5 was required.

### Read Density and CGH Log ratio change at both alignment locations of the chimera.

For this list we required both a CGH and read density change that agreed in direction of change at both alignment locations of the chimera. This allowed the t-test threshold to be reduced to 2.5 while maintaining good separation between somatic chimeras and others. For read density, windows of 100Kbp pair were used for the t-test. For CGH we used 100 probes.

### Supported chimeras with Read Density or CGH Log ratio change at one of the alignment locations.

We started with a list of ~1500 chimeras that were supported by more than one read. Then we selected from this list, a list of chimeras where at least one side had a read density or a CGH log ratio change. We used 100Kbp windows to measure mean read density and CGH log ratio with a t-test threshold of 3.0 to identify CNV sites.

## Tables and figure showing the results of chimera discovery

Supplementary Table 1 Number of candidate chimera junctions from each set of reads by the number of unique reads supporting the junction. The projected column is the estimated number of chimeras in the genome given the number actually found and the probability of having this many reads cover a chimera. For the tumor and James Watson projections, the number of reads used to calculate the binomial probabilities was discounted by 20% to allow for duplicate fragments.

| **No. Reads Crossing a Junction** | **Tumor** | |  | **JC Venter** | |  | **J Watson** | |
| --- | --- | --- | --- | --- | --- | --- | --- | --- |
|  | **Detected** | **Projected** |  | **Detected** | **Projected** |  | **Detected** | **Projected** |
| **> 1** | 155,524 | 239,825 |  | 52,419 | 60,896 |  | 206,951 | 242,738 |
| **> 2** | 1,477 | 5,217 |  | 7,189 | 12,261 |  | 5,129 | 8,993 |
| **> 3** | 488 | 5,424 |  | 2,648 | 8,388 |  | 2,547 | 8,485 |
| **> 4** | 158 | 7,087 |  | 937 | 6,798 |  | 1,298 | 10,159 |

Supplementary Table 2 Number of chimeras at sites identified with each of the four methods

|  | **Method** | | | |
| --- | --- | --- | --- | --- |
|  | **Read Density** | **CGH Log Ratio** | **Both Read Density and CGH** | **Chimera supported by >= 2 reads** |
|  | **CNV at both sides** | | | **CNV at least one side** |
| **Window** | 50 kbp | 100 probes | 100 kbp or 100 probes | 100 kbp |
| **t-test score** | >= 4 | >= 3.5 | >= 2.5 | >= 3.0 |
|  | **No. Read Density or CGH log ratio level changes** | | | |
| **Number of Sites with Read density or CGH change (CNVs)** | 849 | 1442 | 835 | 7,908 |
| **Confidence (bp)** | ±12,000bp | ±15,000bp | ±17,000bp | ±15,000bp |
|  | **Chimeras with both edges at density change** | | |  |
| **Total** | 26 | 32 | 23 | 39 |
| **Validated with PCR** | 15 | 16 | 17 | 13 |
| **Unique to method** | 0 | 0 | 1 | 2 |
| **Supported by Single Read** | 5 | 5 | 4 | 0 |


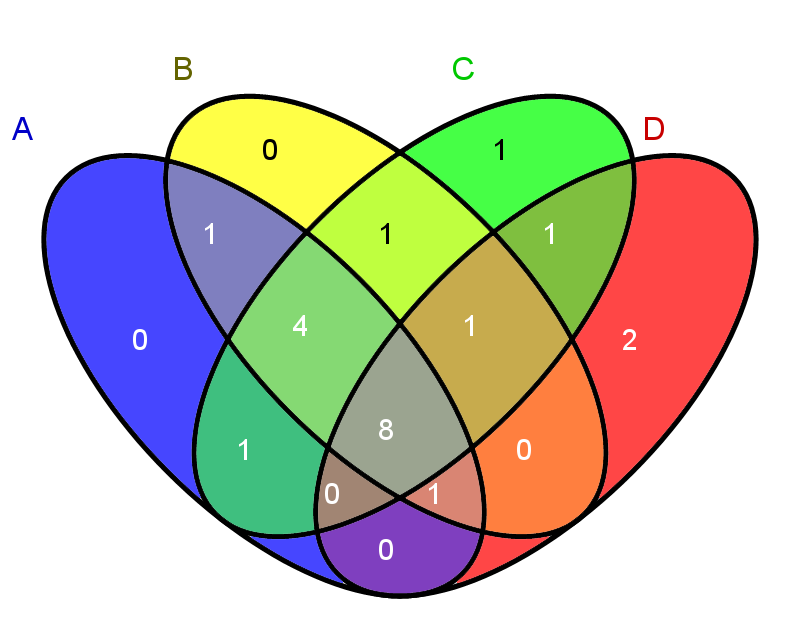


Supplementary Figure 1. Venn diagram of the chromosomal mutations detected by each filtering method. Method A-D are described above.

## Rationale for using this strategy to discover chimeras

The general idea or assumption is that cancer chimeras should align to locations where there is a copy number change or variation (CNV), and artifact chimeras should align to random locations which may or may not, by chance, have a copy number change.

In the following diagram (Supplementary Figure 2) we have two chromosomes indicated by the long rectangles and a set of chimera reads illustrated by the red lines connecting the two alignment locations

Figure 2. Two chromosomes with chimera pairs. Chimera reads could be the result of somatic or germ line mutations together with chimeras formed *in vitro* during the sample sequencing process.

If we take the locations of all the copy number variations (Green in Supplementary Figure 3) and overlay this on the two chromosomes, the result is similar to that depicted in Figure 3:

Figure 3. Chromosomal rearrangements may be indicated by chimeras that join two CNV sites. *In vitro* chimera artifacts should not be related to CNV sites. CNV sites are shown in green.

Chimeras that correspond to CNV sites on both ends of the chimera are possibly tumor chromosomal mutations.

The sites of CNV are detected using a t-test to identify changes in the mean read density along the chromosome. For the t-test we can vary the length of the window used to calculate the mean read density and vary the threshold used to detect a change in the mean read density. The edge detection uses a pair of sliding windows together with a local peak detection algorithm to identify the most probable sites for a CNV. The site of the CNV has some statistical uncertainty and each edge is defined by the position of the peak in the t-test score and the uncertainty is defined by the region where the t-test score is greater than 70% of the peak value. It is possible to adjust the window length and the t-test threshold to change the number of edges detected and the degree of uncertainty (referred to as CNV window) in the edge detection.

We started with ~155,000 chimera locations (see Supplementary Table 1 above) in the MPM tumor sample. Only ~1700 of these were supported by 2 or more unique reads. Using binomial distribution and read density we estimated that around 150,000 of the chimeras were artifacts of the sequencing process and many of the remainder were germ line mutations.

The tumor-specific chromosomal mutations were extracted from the full list of chimeras by identifying chimeras where both locations were at CNV sites as follows.

We can calculate the probability of a randomly generated chimera having both its alignment locations landing on a CNV from the density of CNVs as follows:

Let Nv equal the number of CNV sites in the genome;

W is the CNV window length; and

Ng is the genome size,

Then the fraction of the genome covered by CNV windows is:

Fc = Nv * W / Ng

The probability that one side of a randomly generated chimera lies on a CNV window, P(CNV) is:

P(CNV) = Fc

The probability that both sides of a randomly generated chimera lie on CNV sites is:

P(CNV_1&2_) = P(CNV).P(CNV)

Let Nc equal the number of chimera locations. If they are randomly generated by the sample preparation process, the number of chimeras that we could expect to have with both ends on CNV changes is:

Er = Nc * P(CNV_1&2_)

If the actual number of chimeras with both ends in CNV windows is greater than expected, there is a good chance that the excess chimeras are true tumor chromosomal mutations.

As we increase the t-test threshold, the number of CNV sites starts to drop and the probability that a randomly generated chimera has both ends at CNV sites drops as in the following illustration:

Supplementary Figure 4. Increasing t-test threshold decreases the number of CNV and the probability that chimeras produced in vitro join two CNV locations. True chromosomal mutations related to CNV should remain.

If this process finds more chimeras than we expect from a random process, the excess are probably chimeras from chromosomal mutations.

We then run the chimera-filtering process with several t-test window sizes and gradually increase the t-test score threshold until the number of chimeras selected exceeds the expected number from the random model. Results of this process are shown in Supplementary Table 3 below which shows the number of chimera with both alignments on CNV edges as a function of the window length and threshold used for the t-test. As the number of edges detected drops below 1000 we start to see a clear excess of chimeras compared to the random model. Based on this we created tables of candidate chimera that were used for mutation discovery and validation.

| **t-test Window Kbp** | **t-test Score** | **Number of CNV Edges Detected** | **CNV Window Width bp** | **Number of Chimera** | **Expected False +ves** | **Est. True +ves** | **Est. % True +ve** |
| --- | --- | --- | --- | --- | --- | --- | --- |
| 20 | 6 | 190 | 9474 | 4 | 0 | 4 | 99% |
| 20 | 5 | 355 | 10228 | 9 | 0 | 9 | 97% |
| 20 | 4 | 636 | 10788 | 13 | 1 | 12 | 94% |
| 20 | 3.5 | 971 | 10650 | 20 | 2 | 18 | 91% |
| 20 | 3 | 1943 | 10072 | 29 | 7 | 22 | 77% |
| 20 | 2.5 | 5325 | 9156 | 76 | 41 | 35 | 46% |
| 20 | 2 | 16988 | 8173 | 339 | 332 | 7 | 2% |
| 30 | 6 | 315 | 14156 | 8 | 0 | 8 | 96% |
| 30 | 5 | 458 | 14908 | 10 | 1 | 9 | 92% |
| 30 | 4 | 774 | 15280 | 16 | 2 | 14 | 85% |
| 30 | 3.5 | 1145 | 14750 | 24 | 5 | 19 | 80% |
| 30 | 3 | 2263 | 13432 | 35 | 16 | 19 | 55% |
| 30 | 2.5 | 6002 | 11993 | 109 | 89 | 20 | 18% |
| 30 | 2 | 17212 | 10418 | 500 | 554 | -54 | -11% |
| 50 | 6 | 419 | 23773 | 12 | 2 | 10 | 86% |
| 50 | 5 | 567 | 24240 | 19 | 3 | 16 | 83% |
| 50 | 4 | 825 | 23699 | 26 | 7 | 19 | 75% |
| 50 | 3.5 | 1204 | 22377 | 30 | 13 | 17 | 58% |
| 50 | 3 | 2398 | 20025 | 56 | 40 | 16 | 29% |
| 50 | 2.5 | 5795 | 17355 | 188 | 174 | 14 | 7% |
| 50 | 2 | 15347 | 14522 | 766 | 855 | -89 | -12% |
| 100 | 6 | 449 | 46334 | 22 | 7 | 15 | 66% |
| 100 | 5 | 560 | 46030 | 24 | 11 | 13 | 52% |
| 100 | 4 | 777 | 44133 | 36 | 20 | 16 | 44% |
| 100 | 3.5 | 1130 | 40986 | 46 | 37 | 9 | 20% |
| 100 | 3 | 2164 | 35598 | 111 | 102 | 9 | 8% |
| 100 | 2.5 | 4652 | 30120 | 359 | 338 | 21 | 6% |
| 100 | 2 | 11340 | 24041 | 1197 | 1280 | -83 | -7% |

Supplementary Table 3. The number of chimeras with both alignments on CNV edges as a function of the window length and threshold used for the t test on read density. The list produced using a window of 50Kbp and t test threshold of 4 was validated using PCR, 14 chimeras were validated as being in the tumor and not in normal lung tissue.

Columns in Table 3 above are:

| **t-test Window Kbp** | The length of window used for calculating mean read density. A pair of adjacent windows was used in the t-test. |
| --- | --- |
| **t-test Score** | The t-test threshold used for detecting copy number changes or edges. |
| **Number of CNV Edges Detected** | The number of copy number changes detected using these settings |
| **CNV Window Width bp** | The width of the region or, uncertainty in location of the CNV edge. This was calculated as the region where the t-test score was ≥ 70% of the peak value. |
| **Number of Chimera** | The number of chimeras, where both alignments fell on CNV edges |
| **Expected False positives** | The expected number of chimeras, based on the random model. As the number of edges increases we can expect more chimeras by chance. This is also an estimate of the number of false positives in the list. |
| **Estimated True positives** | The balance of chimeras is likely to be true positives. This is the difference between the number of chimeras and the expected false positives. |
| **Estimated % True positive** | This is the percentage of expected true positives as a function of the total chimeras selected by these settings. |
