# Supplementary material for: Second Generation Sequencing of the Mesothelioma Tumor Genome
Source: PLoS One. 2010 May 13;5(5):e10612. doi: 10.1371/journal.pone.0010612 (PMC2869344; doi:10.1371/journal.pone.0010612)
Supplement: Table S1 — Chimera read sequences for tumor chromosomal mutations. (0.07 MB DOC) [file pone.0010612.s003.doc]

# Supplement 3: Chimera read sequences for tumor chromosomal mutations

| **Chromosomal**  **BreakPoint(bp)** | **Chromosomal**  **BreakPoint(bp)** | **ReadSequences** |
| --- | --- | --- |
| **Intrachromosomal** |  |  |
| Chr2:115,918,613 | Chr2:116,314,797 | >ECLRS0G02IRNUN  ACACAGCAGGAAAACACGTTCTTCTAAGAAAGTAGCTTACCTGCATAGTATAATTCACTCCAGCTTTTA  TTAGGTGCTTGATTAATTCTGCTGAGTGTTGGAAATGAACTTTTAGAAACACTTTTGAAATATAATAGA  TGAGTAACTACAGAGAAAGACTCAGCACTTCTTGGAAAGAAACAAATTCCCAAGAGAATCTGTGATTGA  CAGAGACTTATTATAACATAATTCATCAATGAGAATATTTTGATTTACTAA  >EQFUB5D02HEAIM  AACTAAAAACATATTAAAAATAATAGTAGGGAGAATGGAATAATAGAGAAGCTGTGTTGCGTATCTCAC  AAGTGAAAAAACACAGCAGGAAAACACGTTCTTCTAAGAAAGTAGCTTACCTGCATAGTATAATTCACT  CCAGCTTTTATTAGGTGCTTGATTAATTCTGCTGAGTGTTGGAAATGAACTTTCAGAAACACTTTTGAA  ATATAATAGATGAGTAACTACAGAGAAAGACTCAGCACTTCTTGGAAAGAAACAAATTCCCAAAG  >EQEF71401CZF4V  AAAAACATATTAAAAAATAATAGTAGGGAGAATGGAATAATAGAGAAGCTGTGTTGCGTATCTCACAAG  TGAAAAAACACAGCAGGAAAACACGTTCTTCTAAGAAAGTAGCTTACCTGCATAGTATAATTCACTCCA  GCTTTTATTAGGTGCTTGATTAATTCTGCTGAGTGTTGGAAATGAACTTTCAGAAACACTTTTGAAATA  TAATAGATGAGTAACTACAGAGAAAGACTCAGCACTTCTTGGAAAAGAAACAAATTCCCAAGAAG  >EPEWS9O01C2NHC  CTGCATAGTATAATTCACTCCAGCTTTTATTAGGTGCTTGATTAATTCTGCTGAGTGTTGGAAATGAAC  TTTCAGAAACACTTTTGAAATATAATAGATGAGTAACTACAGAGAAAGACTCAGCACTTCTTGGAAAGA  AACAAATTCCCAAGAGAATCTGTGATTGACAGAGACTTATTATAACATAATTCATCAATGAGAATATTT  TGATTTACTATTAAATTATGCCCTTCTACTTTCTTAATTATTGTT |
| Chr3:98,917,287 | Chr3:99,007,383 | >EPGAQ4K01BP8QS  TCAATTGATTGGTCTCTCCTCTGCAGATTAGAGGCTTATATAGTAATACTCAGGTGTTAAATGCACCTA  TAATACAACTCCACTCTGGGGATATAGGGCAGTTTTATATTTATTTAAATTTCTGTCTTCACTCTTAAT  ATATTCTACCTCCCAGAAACAAACTGAGAAAAATGCCTGCATGAGATTATTTCATGACAGAATGAAAAG  TTATCATGCTAAGAGTGTCTTACTCTAAGGAAATTTTTATATTCAGTTAA  >EQFT8KP02IGK0P  TAATACTCAGGTGTTAAATGCACCTATAATACAACTCCACTCTGGGGATATAGGGCAGTTTTATATTTA  TTTAAATTTCTGTCTTCACTCTTAATATATTCTACCTCCCAGAAACAAACTGAGAAAAATGCCTGCATG  AGATTATTTCATGACAGAATGAAAAGTTATCATGCTAAGAGTGTCTTACTCTAAGGAAATTTTTATATT  CAGTTAACTCATGATTTTGTTTTTTCTTTAGCCCTACTTTAAATTGTTTTTCAAAGGGGCTAAATTTAT  ATTAA  >EQJEM6Z01CA7HW  AACTTTTCATTCTGTCATGAAATAATCTCATGCAGGCATTTTTCTCAGTTTGTTTCTGGGAGGTAGAAT  TATTAAGAGTGAAGACAGAAATTTAAATAAATATAAAACTGCCCTATATCCCCAGAGTGGAGTTGTATT  ATAGGTGCATTTAACACCTGAGTATTACTATATAAGCCTCTAATCTGCAGAGGAGAGACCAATCAATTG  AAATGTAGTAAGGATACAGAGGACTTAATACTAGGAAACCAAAGTAGGAAAATTCTAG |
| Chr6:64,615,701 | Chr6:57,319,605 | >EP0VDDS01C6NYM  ATAAATGGAGACAAGTCCAGAATAGGGGTATTAACTGTGCTGTTGTCCTTTGTATGCCTTCAGAGGGGTG  GGGAGTAGCAGAATGGATATATTCACAGGGCCTGGGCCAAATGGATCTGTTAAGCAACTACTTCCTCCTC  CCTTTGTAACTCAGATAAATCATTTAATATGAATCTTCACTCATAAAATTGGGTATGTACAAATATAGAC  ACTAAGTTATTTCTCATATATTTTGATAAGATCAACTAAACTTGAATAGTA |
| Chr8:6,749,979 | Chr8:34,877,265 | >EP93YF002F66PZ  CTATTTTTGGTGCGAGTTTGGCTTTGGGAAATGCTTTGGAGCTTCTTCTCAGTCCAGCCTTATTATGGA  AGTCCTAAAACTAGGGACTTATGGGAACAATCAGATTCCCATTCTTAGAATTATAAATACTATACTTGT  TGGGGCTCAGAACACAATATCCCAAAGTATGGCACCTTGGCATATGGAGTATTTTAAGCTGAAGAAATT  TGAGAAAATTGAAAAAGCAGGGTGGTCTTTCTGAACTTTTCCCAACCCCCTCTCTCCTGATGTGGA |
| Chr8:6,786,552 | Chr8:34,863,640 | >EC9VU5F02FN3O9  AGAATTGTTTGGGGAAGTTGAACTGCCATGTAGTTTTTTGTTGGGAGTGCTAGTCAATACTACAGATAA  TCTGAAGCTGGAATGAAAATTACAGAGATATTCCTGTTCTCATGGTAGTGAATAAGTTTCACAAGATCT  GATGGTTTTATATAGGAGTTTCCCTGCAGAAGCTCTCTCTTTGCCTGCTGCCACCCACGTAAGACTTGA  CTTGCTCCTTT  >EQLUM5D01DI0S1  CATGTTGTGTTCCCATGCATGAGGTGCCCTTAGGGTTAATAGCCATGGAAGGAATGAATGGGGAATAGA  ATTGTTTGGGGAAGTTGAACTGCCATGTAGTTTTTGTGGGAGTGCTAGTCAATACTACAGATAATCTGA  AGCTGGAATGAAAATTCAGAGATATTCCTGTTCTCATGGTAGTGAATAAGTTTCACAAGATCTGATGGT  TTTATATAGGGAGTTTCCCTGCAGAAGCTCTCTCTTTG |
| Chr8:21,254,202 | Chr8:72,593,210 | >EP8U4WM01EDG2L  TGTTTGATTAGCTGCCTTTGCAAAGGCCGAGTGCATGGTAGATATAAGAAAAACATCTGAAGGCTGAGG  CTTTCATGGGAGGTAATGGTCACACTATTCCTAGACATGACAGAGAGAAAATAGCATTGTCGCTTCACC  TTTACATCGTCTTCAAGTTCTTGTTTGTGAGTGGCCCTACCGGTATTAGGTGGATCGCCTTTTTCAATA  CCAGGACAAGGAAAATGTCACTCAAAACTGCTAAGAATCTCAAGAATTTA |
| Chr8:34,816,557 | Chr8:72,573,255 | >EQGINZ201ESE0F  AAATAAACCTAAAGACTCTTAAATCCAATCACATTAGTCTCTTGCCATGAGAGAACCTATTTGCATCGA  AATATTCTAATAGAGTAGAATTATGTTCAGACTTTTCCTATTGATTTAATCTGATCCATCTTTCCAAGC  AATTATCTTTTAAAGAACAGGGAAAATATTGTATATTAAATATGTAATGCATGTAGAGATTAATGCTTT  TAGTAAAAAGTAATTAAATGTATTCAAAGTCTGTACCTTTCATTATTTATTTTTGGTTCTTTTGCTAA |
| Chr8:34,816,593 | Chr8:72,577,581 | >EQB7H4Q02IQKSN  CTAAAATAAAATTTAAGTAAACATTCTGCATTTTATCTGGCAACTCTAGTGACTTGATAAAATCATAGA  TTATGACTTTGAACTTATCACGCTACAGATAAGGTGCCTGAAGCATGGAGATGAAGAAACTGATGTAAC  TCAAACAGGCACCCAGTAACAGCCTTGACTACCACCCAGGCCTTGAAACCGCCAGTCAACCTACCTATA  TTTTATAAAATATATATTAATTTAGACATAAAACTAAACATTTTAAATTTTTTTAAAATAAGTATTTTA  GCAGG  >EQFT8KP01CB4B8  ATGAAGAAACTGATGTAACTCAAACAGGCACCCAGTAACAGCCTTGACTACCACCCAGGCCTTGAAACC  GCCAGTCAACCTACCTATATTTTATAAAATATATATTAATTTAGACATAAAACTAAACATTTTAAATTT  TTTTTAAAATAAGTATTTTAGCAGGCAATGATTTGAGTGGGTCTAATATCAGCCCACTGATAGTGAGTG  ATGCCTCCAAGTTCCTGGAATCACCATGATCCAAAACAACTAACCAGCGAGCCTCCAGCTA  >ECRAQM101D9VW3  GTTTCTCTAAAATAAAAATTTAAAGTAAACATTCTGCATTTTATTCTGGCAACTCTAGTGACTTGATAA  AATCATAGATTATGACTTTGAACTTATCACGCTACAGATAAGGTGCCTGAAGCATGGAGATGAAGAAAC  TGATGTAACTCAAACAGGCACCCAGTAACAGCCTTGACTACCACCCAGGCCTTGAAACCGCCAGTCAAC  CTACCTATATTTATAAATATATATTAATTTAGACATAAAACTAAACAATTTTAAATTTTTTAA |
| Chr8:36,550,070 | Chr8:72,552,661 | >D78KKVM01CVP4H  CATTTGCAATCCGAATTTTCTTTGGAAAAGGAAGGATGAATGTACCACGTTTTATTTTTCTGTTTATCA  GCTGATAGATGTTTGCATTTGGCTATTATGAATAAT  >EP1J2MD02HNRCT  CCAAATGCAAACATCTATCAGCTGATAAACAGAAAAATAAAACGTGGTACATTCATCCTTCCTTTTCCA  AAGAAAATTCGGATTGCAAATGAATGGTCTTCTGGGGCTGAGAGTCCAAGACTTCTGTTTACTCCATGT  TTTATATTGTTTTATACTGTCTGTGTCCTTTTGGGGTAAAGCTTTGTCTCTTCTTCTTCCTCCACCTCA  TCATATAAAAGCTAACTATTAAAAATCTGTACCATTCTAGGTNTGCTACTTGAATTAACTCATTTAA |
| Chr9:21,957,500 | Chr9:22,075,035 | >EKHCFIA01BXT4P  CTGGGAGCCGAGTCGGATTCCGAGACTATGGGCCAGGGTTGGCTGGATTCAGTTACCTGGCTGAGGCCT  GGTGAGCAAAATATCCCAAACCTCGCGTGATCTGGAAGGGGAAGCCGGATAAATACGGAGTGAACATTT  TTCCACAGAGAACTCAAATATGGTAACATTCTCATAGCTTTGATAATCCCCAATAAACTGTGGTTACTA  AAATGCATGACAAAAAGAACTTCTATCTATAAAGAACTTCTTTATACATTTCCAATACTTGGGTTA |
| Chr10:84,083,200 | Chr10:84,157,179 | >EP0UBPO02G1MZ6  AGCACAGTAAGGCATTATTTTGTGTGTCTTTATTCTCTTCACACCTTCTTTATGAGGGACAAAGTCATT  TTCCCATTTGACAGAAGAGTTAACTACCACACAGAGGTTCCTTAACTTTTACACAATCACAAATTCATA  AGTGATGTAGCAGTAACTCTTTAATCTCTGCCTTTTGAAATCTCATCCATTTTCCCACATGCAACTCAG  AGTCAATTCAGCTGTGTACTTTTCCCCAGTCTTCCACCTGGCATTAA |
| Chr11:5,5762,996 | Chr11:55,765,854 | >EPVB62D01DPY0K  TCTCTAGCTGGAAGTACAGTGATGGGGGTTGGTTTTCTTGCTGTATAGGACTAGAGTCACAGAGTCAAA  ACATAAAATGGGGAAAGGATATCCTTTTCAATAAATGGTGCTGGGAAACCTGGCAAGCCAATGTAGAAG  AATGAAACTGTATCCTCATCTCTCACCTTATAAAAAATCAATTCAAGACTGTTAAAAGACTTAAATCTA  AAACCTAAAACTATAAAAATTCTAGAACACCATAAAAACTCTTTTAGACATTGGCTTAGGAAAATAATT  CACAGCTAAG |
| Chr11:5,9,018,943 | Chr11:59,023,522 | >EQBY87O01AWTD5  CATGGAAAGACAGAGAAAAGAAAATCATTGCTATCATAAGGTTTCAGAAAACAATACCCTATAATGAAG  GCCTCAGAAGTAGCCTCAGAAGTAAAAGTTTTTCTCTGACCTCCTCCTGCCTTCCTGTGTCAGGTGGCT  GGACCTTCAACGTTTTTCAAACTTCTCACCATGACCACCATTACCATTCTAAGTCATGTCTTCATTCTT  TCTCTCATGGGGCTATTATAGTAGGCTCTGAATTGCTGCCTGGTTTTTGTCCTTA |
| Chr12:127,300,384 | Chr12:126,530,113 | >EQBY87O01DO7D3  TCTGTGCCCTTGCGGGAAACAAGTTTCTCTCAACAAAAGCACCGGTGGCATTTGGGCAGGACAGAGTTC  ACCGTGTGGGACGATGAGGCGGTTTCCTATTCCTGGCCTGTGCCATTGGAACAACTAAAAGTGTCTGTG  ATTCTACTTGGGACCAAACCCTTCACCAGACACAGTCCATAAAATCGCATTTTGCCAAGGAGACTAAGA  CAAAGTAGCATCTGAAATTCAGCAGGCATTATGAGACACGCCCCAGCCCCGGGAA |
|  |  |  |
| Chr17:23,113,949 | Chr17:65,721,910 | >EPF2LCE02JQ3DI  GGCCACCATCCTCCAGAAACCCCAGAATGGTAGATCCACTGAGAGCTTGTACTATGCACCTGGAAAAGTG  GCAGACACTCAACATCAGCCTATGAAAGCATGAGCCAGGGGCAGAGGCTCTGGAACAGCCCCGCTCCGCC  CATTATTGGACATCTGTTCATCAACTCAGATTCAGCTCCCTCATCAGTTCAAGGAGGGAGGGAACACCCA  CAGCCCAGAGTTGTTAACAGTATTAGGAGGTG  >ECYR39402FTRQS  TCTCTCCCACACACCTCCTAATACTGTTAACAACTCTGGGCTGTGGGTGTTCCCTCCCTCCTTGAACTGA  TGAGGGAGGCTGAATCTGAGTTGATGAACAGATGTCCAATAAATGGGCGGGAGCGGGGGCGTGTTCCAGA  GCCTCTGCCCCTCGGCTCATGCTTTCTATAGGCTGATGTTGAGTGTCTGCCACTTTTCCTAGGTGCATAG  TACAAGCTCTCAGTGGATCTACCA  >EDB1XSD02GY0YW  TCTCTCCCACACACCTCCTAATACTGTTAACAACTCTGGGCTGTGGGTGTTCCCTCCCTCCTTGAACTGA  TGAGGGAGCTGAATCTGAGTTGATGAACAGATGTCCAATAATGGGCGGAGCGGGGCGTGTTCCAGAGCCT  CTGCCCCTCGGCTCATGCTTTCATAGGCTGATGTTGAGTGTCTGCCACTTTTCCTAGGTGCATAGTACAA  G  >EP6EJ3Y01ENOTU  CCCAGAATGGTAGATCCACTGAGAGCTTGTACTATGCACCTGGAAAAGTGGCAGACACTCAACATCAGCC  TATGAAAGCATGAGCCAGGGGCAGAGGCTCTGGAACAGCCCCGCTCCGCCCATTATTGGACATCTGTTCA  TCAACTCAGATTCAGCTCCCTCATCAGTTCAAGGAGGGAGGGAACCACCCACAGCCCAGAGTTGTTAACA  GTATTAGGAGGTGTGTGGGAGAGAT |
| Chr17:26,853,914 | Chr17:47,815,374 | >EQJETID02I9JQ1  CATGGTGAAACCTTATATCTACTAAAAATACAAAAATTAGCCAAGCATGGTGGCGCACACCTGTAATCAC  AGCTACTTGGGGGGATGAGGCAGGAGAATTGCTTGAACCCAGGAGGCGGAGGCTTCAGTAAGCCAGTCTG  TGGTCCTTTGCTATGACAGCCGTAGCAGACCAATAGACCAATATACCAATATCCCTTCTCCTCCCCTAGG  AAGGCTTCATGGAAACCCCAACCCAAGCTCAGTCTGGATTTTTTGCCCACCTT  >ECRAQM102GVFDJ  AATCCAGACTGAGCTTGGGTTGGGGTTTCCATGAAGCCTTCCTAGGGGAGGAGAAGGGATATTGGTATAT  TGGTCTATTGGTCTGCTACGGCTGTCATAGCAAAGGACCACAGACTGGCTTACTGAAGCCTCCGCCTCCT  GGGTTCAAGCAATTCTCCTGCCTCATCCCCCCAAGTAGCTGTGATTACAGGTGTGCGCCACCATGCTTGG  CTAATTTTTGTATTTTTAGTAGATATAAGGTTTCACCAT  >EP93FDM02IYJLM  TTGGGGGGATGAGGCAGGAGAATTGCTTGAACCCAGGAGGCGGAGGCTTCAGTAAGCCAGTCTGTGGTCC  TTTGCTATGACAGCCGTAGCAGACCAATAGACCAATATACCAATATCCCTTCTCCTCCCCTAGGAAGGCT  TCATGGAAACCCCAACCCAAGCTCAGTCTGGATTTTTTGCCCACCTTCTCACCCTGATAACAAAAGAGAA  CTTTTTAAAGGTCAATTCAATTCAACCTTTATTTTTTATTTTTTGAGATACAGTCTTGCTCTGTCACCAG  G |
| Chr17:47,110,011 | Chr17:68,044,222 | >EDB1XSD01E1T8Y  CCGGCCCTTCATTGAGCTCTAAAAATAAACTCCGTAGGGATATTGATATGACGTATTTCATCAAGCGGGG  AAGCTTGATGAAGACGGTCCTGGAGACAGGGTGGAGTAGAGAGAGAGAGAGAACTGTCATAAATTACCTA  AAGTACTTTTTGAACTACTGTGG  >EC6R2IL01ER2HL  CCGGCCCTTCATTGAGCTCTAAAAATAACTCCGTAGGGATATTGATATGACGTATTTCATCAAGCGGGGA  AGCTTGATGAAGACGGTCCTGGAGACAGGGTGGAGTAGAGAGAGAGAGAGAACTGTCATAAATTACCTAA  AGTACTTTTTGAACTACTGTGG |
| Chr17:65,036,914 | Chr17:47,505,787 | >EP79KJK01DDMKX  AGTCTACCATTGTGAATCACCAGTTTAATCCAATGATTCTCAACTGTGGCTGCTCATTTATAAAACTCTA  GTGAGCTTTCAAAATCACTGCCCCCTGATACTTTTTCTTCCTATGATATTAAGAAGCCCTGAGGCTTGTA  TCTTTGTGGTTGAAGGAGATTTTCTGACATAGGATGAGTGAAACTGAAGACCCACTCCTTCTGGAACTGA  CAGTGGGCTTAGGGGACTTTAAAACAAATAGAAGAGAGACAAAGAGCTTTAA  >EC8IP8301COW88  AAAGTTTGAGAAAGCTGAGTCTACCATTGTGAATCACCAGTTTAATCCAATGATTCTCAACTGTGGCTGC  TCATTTATAAAACTCTAGTGAGCTTTCAAAATCACTGCCCCCTGNTACTTTTTCTTCCTATGATATTAAG  AGCCCTGAGGCTTGTATCTTTGTGGTTGAAGGAGATTTCTGACATAGGATGAGTGAAACTGAAGCCCACT  CCTTCTGGAC |
| Chr17:47,696,961 | Chr17:63,830,958 | >EPGAQ4K02HFYYK  TTTCCTAGTTTTCATTGACTTCTCAGACCTAGAATTTATAAATTAGAGTCTGCAGTGACTCAAACTATCT  GAACTTCATTTTACTCATATGTAAGTGAGGATAATAATGCTACTAATTCCAATTTTACATACATAAGCTG  GTGAAATGACCACTGTAAACTGGATATTGTCTAGGACTCCATTTATTACCCGAGTCCTATGTGTCTTCAC  TTTAATTTTGTGGCCATGATGATGTTTCAGGTCTCAGAGTCT  >EP6ENZ402I3I64  ATTAATAAGAGGGTTCTTTCTCTCTCTCTCATTAAGTATGTCTATGGCTTTGGGGTAAAGTCAAGTTAAT  TTGAGTCCTTTTCCTAGTTTTCATTGACTTCTCAGACCTAGAATTTATAAATTAGAGTCTGCAGTGACTC  AAACTATCTGAACTTCATTTTACTCATATGTAAGTGAGGATAATAATGCTACTAATTCCAATTTTACATA  CATAAGCTGGTGAAATGACCACTGTAAACTGGATATTGTCTAGGACTCCATTTA |
| Chr19:38,085,640 | Chr19:38,091,701 | >D8LTS5201DQXSY  GGGTTGCCAATGGGCAGAGAAAGCAAGACAAAATACCCATTATTTGCCTTCTATTTCCTGTTCTTTGTGC  ATGTCAGAAATTGTTCTGCCTACTGTTTGTAGTGTTTCTTTCCTAGGCTTTACGCAGTTTCCTCTCACAC  ACGGACAGATCAGTACCCAGCCAAAGACCCTTCAAGGTCTCTAGAGCCCTTTCTCGCTCCGTGAAATGCC  CTCTGCCCTGCCC |
| Chr21:18,540,069 | Chr21:33,670,572 | >EP0UBPO02HGX4N  TGAGCCACCGCAGTCGGCCAAAAATTATTTTTAAAAGATTATTAGTGTGCAAAGGAAAGGCATCTACACG  CAATGACCACACTTCAGTTCCTTGCCAGTAGTCCTGCTGAGAACAAAGACCGAATGCCCAGGCATCCGGA  GGCGACCCACAGCGAACTGATCTGGTCACAGAAAGCATCCGAGTGAACACGGTGCTGGGACCAAAGTCCA  GTCAGAGCGACAGGAGGGTGGCTATTTCNGGCGGTGGCTTCTTCG  >EPLZZ1G01EM2ZL  TTGGTCCCAGCACCGTGTTCACTCGGATGCTTTCTGTGACCAGATCAGTTCGCTGTGGGTCGCCTCCGGA  TGCCTGGGCATTCGGTCTTTGTTCTCAGCAGGACTACTGGCAAGGAACTGAAGTGTGGTCATTGCGTGTA  GATGCCTTTCCTTTGCACACTAATAATCTTTTAAAATAATTTTTGGCCGACTGCGGTGGCTCATGCCTGT  AATCCCAACTCTTTGGGAGGCCGAGGCGGGTGGATCAT |
| Chr21:20,864,799 | Chr21:18,281,613 | >EPMLJQA01DQK6J  TGGTTACCTAGGAGATGAGGACAAAATGAAAATTGATTGACTTATCCAATTGATTGCTTCAAACTGAGCT  TCAAGTGGCAGCTGTGAGTGCTGCACATAAAAGGTAGCAGTTTGAAAGTGATTCTTTTTATTTACCACAT  CACTAATGATGCCAAACACACTACTGGCCTGACACACTTAGAAAACGTTTCTTCCTTCTATATGGTTTNA  AATAGTTTGAGAAAGTTTGCTGTTTAATTTTCTGAAATTGTTTGGTAGAATTT  >EP6EX5Z01DJDE1  ATAAAAGGTAGCAGTTTGAAAGTGATTCTTTTTATTTACCACATCACTAATGATGCCAAACACACTACTG  GCCTGACACACTTAGAAAACGTTTCTTCCTTCTATATGGTTTGAAATAGTTTGAGAAAGTTTGCTGTTAA  TTTTCTTGAAATGTTTGGTAGAATTCTCTCTTTTCCTGTTTTTTTTTGTGAGAAGTTTTTGATTACTAAT  TTAATGTCTTTACTTTTTAAAGCTTATTTTCAGATTTTGTATTTCTTTAAGTTACTTTTAATATTTTGTG  TCCAGGCCTTATCCATTTCATCTTAG  >EDHM8M002JIVCA  GGATAAGGCCTGGACACAAAATATTAAAAGTAACTTAAAGAAATACAAAATACTGAAATAGCTTTAAAAA  GTAAAGACATTAAATTTAGTAATCAAAAACTTCTCACAAAAAAAACAAGGAAAGANAGAATTCTACCAAA  CATTTCAAGAAAAGTTAAACAGCAAACTTTCTCAAACTATTTCAAACCATATAGAAGGAAGAAACGTTTT  CTAAGTGTGTCAGGCCAGTAGTGTGTTTGGCATCATTAGTGATGTGGTAAATAAAAAGAATCACTTTCAA  ACTAGCTACCTTTTATG  >EQJB72S01D7688  AAAAGTAACTTAAAGAAATACAAAATCTGAAAATAGCTTTAAAAAGTAAAGACATTAAATTAGTAATCAA  AAACTTCTCACAAAAAAAAAACAGGAAAAGAGAGAATTCTACCAAACATTTCAAGAAAATTAACAGCAAA  CTTTCTCAAACTATTTCAAACCATATAGAAGGAAGAAACGTTTTCTAAGTGTGTCAGGCCAGTAGTGTGT  TTGGCATCATTAGNGATGTGGTAAATAAAAGATCACTTTCAAACTGCTACCTTTTANGTGCAGCACTCAC  AGCTGCCACTTAG |
| [Chr21:19,693,189](http://synasite.mgrc.com.my:8080/chimera/ttest/examples/chr21_19692939..19693439-chr21_31062171..31062671.html) | Chr21:3,1062,421 | >D72V04201DQXCE  TAGAAAACAAACTATAATTGGGTCCATAAAGATAAAAATAAATAGGCAAGATATAGGAAGTANAAGGAAA  GAAGTTAGCATTAAATTATCAGATATGCTGAACATTAGCATTTGAAAATAAAAGCGTGGANACTTTTCCT  TACTAGGAAAAAAACCCTGTTTATAGTGTTTATGATTTTAAAAGTTGGAGCAGGAAACATTGCCTGTGGT  GTTGTATATTTGAAGATTATAAAAAACTAATGACCTTATTTTCACTTGATTCCCTTTATAAATATCCTGT  CTCC  >EQLUM5D02G4WS5  TAGGAAAAAAAACCCTGTTTTATAGTGTTTATGATTTTAAAAGTTGGAGCAGGAAACATTGCCTGTGGTG  TTGTATATTTGAAGATTATAAAAACTAATGACCTTATTTTCACTTGATTCCCTTTATAAATATCCTGTCT  CCAATAGGTTACATTCTGGAGACTAGGACTCCAATATACCTTTTTTTGAGAGGACACAATTCAACCCATT  AAATGGTTCCCCTTGCCTGAATACAATACAAAATAGCTAAATATTTATACTATGAAATATGTGTATGTGT  AGTGAAACA |
| Chr21:21,167,077 | Chr21:27,861,346 | >EC96DKP02H0D8C  TGGGACTACAGGCACAAATCCATGCCCGGCAATTTTTTTAATTTAAAAATGTTTTTTGATGAGGCTAGAC  AGGGCGCTAGTGGGGGAGAGAAAGTTCTGGAGCATTCTTTCTCAATTGGTAGTGAATGGCTGGGAAGGAT  TAGGAAAGTTCTAGGATATTAGTAATCTAAAAGAAAACAGGGCAGAGTATGGAGGGGATGTGGACAAGAA  AACCTGAAGTTAGAGA |
| **Interchromosomal** |  |  |
| Chr9:31,759,669 | Chr11:84,923,849 | >EQEF71401E2E54  CAAAACAAACAAAATTCCTTACCATGGTCTACAAATCTCCCTATATTTTCCTTTCTGGTAGAGGTTTCAG  TTAAAGTACAGAAAATAGAATCAGGAAACCATTCCTAAATGGAATAATTGTTGATGTGATTCAAACAAAG  GTTTTTGTTATATGAAACTCCATTGTTTTCCTAAGAGTTTGAGTTATATTAAGAGAGTAAAACTGGCCCA  GTTGTGCCTTGGAAGTGATATTTATGGTTTCTTTTGAATAAACATAAAATCCACCTCTCTGTCTTAAACT  GAGAAACTTTCATT |
| Chr10:117,768,069 | Chr17:48,272,943 | >ECLRS0G01C6IAJ  TTTGAAGGCCAGTCAATAAAAGTTATTAGTCACTTTTCAAATTCCATAAACTGCCTTAGAGATTACCCAG  ATTACATGATAGCTGATTCCATGGGTGATTCAGGATACAGAAAACCTGAAAGAATTACTTGGAGAACAAG  AGAGAATGAAGCCAAATGAGCTGCAAGGGGAATGTCAAAGAGTCAGGAGAGATCTCTGTGGCCTGGGAGC  GTCTGAGAAGGCATAAGGAGGTGACAAATGTCTCTTGG |
| Chr10:121,001,050 | Chr17:21,238,007 | >EKHCFIA01D1UQO  AACGCACAGGCCGGCCCCCATGACAAAGAATTATCCAGCCCCAGATGGCAACGGTGTCAAGGTTGACAAA  CCCTGCTTTAGGTGTGTAGATGCTAGAAGGTTTTTTGTTTTATTTTCTTTCCCCCAGACATCTTTCATCA  TCGCTTTAGAAGACAGCAAGAAAATAACGTGGGAGTATGACAATTACAACTCCAGATAAATGTTCGCAGC  CTATCTATCTCGTGGGGATCTCGTCACGTGTCAATGACATTTCAGTCAGTG  >EDDX9G001BEJX9  CCCTTGATGATCTTATGGGATCACTCTCATACACACCCTCCATCACTGACTGAAATGTCATTGACACGTG  ACGAGATCCCCACGAGATAGATAGGCTGCGAACATTTATCTGGAGTTGTAATTGTCATACTCCCACGTTA  TTTTCTTGCTGTCTTCTAAAGCGATGATGAAAGATGTCTGGGGGAAAGAAAATAAAACAAAAAACCTTAC  TAGCATCTACACACCTAAAG |
| Chr15:23,876,380 | Chr6:67,079,455 | >EP61YAF02HAGHF  TTGATATTAGCTGTAGATTTGTGTTGGTGTTATTTATAATGTTAAGGAATTTCTATCCCTATTTTACTGA  GATTATTAATCAGAAATAGTGTTGAGCTTTGTCAAATATTTTTATACATTAACTGAGACGCTTACACAGC  TTTTTCTTTAGTCTGGTCATATGATATTTTGAAGGTTTTCAAAAGTTAGCAGTTACTAATAGCTGAATAA  TCTAATTGTTAGCCCCTTTTGCTCCTCTAGCTCCTAACAAAAAAAAAATTAGACCAAACCTAACTTTTCC  CTCTGTTTAA |
| Chr17:61,459,185 | Chr21:21,169,765 | >EPKTF5D02I139E  TAATAAAGGCTGTGGATGATGTTATCTTCTTCCAGAGAGGCTCTTCTCTCACTTCTGAGAGGCAGCTGAG  AGCATTAACTTGCATATATGTCTCAGTTAAAATATAGTAGAAACAGCATATTCGATTAAGACCTTGCTAT  TCCTCTTACCAATGGTACACATGGGAAAACACTGGAAAATGGAGCATCAAGTCCTAAATCTTTAAGCAGA  ATAATATATTTGTACTAATAAATCTGTAGAAAAGTTTAA |
| Chr21:35,609,289 | Chr17:58,726,449 | >D8SP0DK01B0DEL  TTATACTTCAGCTCTCAGTGGGATCTGCCTCCAACTCCCCACGCTGGATTGCTTTGCTACATTAGGGGCC  AATCAGCAGAAGCTGTTTATCTCTCAGAAGAGTATGTCAACATGAGAACAAGTTATCTTAATAGACTATT  TCTCAGAAACTGAGGCCTTTGAGTCTGTACTTATTCCATGTTATAAATTAG  >EQFT8KP02JYCUO  TGAATATGACTAATGCTAATTTATAACATGGAATAAGTACAGACTCAAAGGCCTCAGTTTCTGAGAAATA  GTCTATTAAGATAACTTGTTCTCATTGTTGAACATACTCTTCTGAGAGATAAACAGCTTCTGCTGATTGG  CCCCTAATGTAGCAAAGCAATCCAGCTGGGGAGTTGGAGGCAGATTCCCACTGAGAGCTGAAGTATAAAA  TCATTCCAACAGGGAGCTCGAGAGTAAACCCAGGAG  >EPVYR4C01CRIS8  AAAGGCCTCAGTTTCTGAGAAATAGTCTATTAAGATAACTTGTTCTCATGTTGACATACTCTTCTGAGAG  ATAAACAGCTTCTGCTGATTGGCCCCTAATGTAGCAAAGCAATCCAGCTGGGGAGTTGGAGGCAGATTCC  CACTGAGAGCTGAAGTATAAAATCATTCCAACAGGGAGCTCGAGAGTAAACCCAGGAGATATCCACTTTC  CAAAGCTTAAAACATAGCATTGTTCAGCATTAAGA  >EQK8B9O01ETVBM  GAGACTTCGTTGCTTTCTAAATAGACTTATCTCTTCAGGTCCAAGATTACTTTACATGTTGTTTCAAAGG  AGGCTTTCATGAATATGACTAATGCTAATTTATAACATGGAATAAGTACAGACTCAAAGGCCTCAGTTTC  TGAGAAATAGTCTATTAAGATAACTTGTTCTCATGTTGACATACTCTTCTGAGAGATAAACAGCTTCTGC  TGATTGGCCCCTAATGTAGCAAAGCAATCCAGCTGGGG  >EP61YAF02F8785  ATACTTCAGCTCTCAGTGGGAATCTGCCTCCAACTCCCCAGCTGGATTGCTTTGCTACATTAGGGGCCAA  TCAGCAGAAGCTGTTTATCTCTCAGAAGAGTATGTCAACATGAGAACAAGTTATCTTAATAGACTATTTC  TCAGAAACTGAGGCCTTTGAGTCTGTACTTATTCCATGTTATAAATTAGCATTAGTCATATTCATGAAAG  CCTCCTTTAGAAACAACATGTAAAAGTAATCTTACGG |

Read sequences for validated chimera. Sequences in grey were treated as duplicates of other reads and not counted in when determining if a chimera was supported by more than one read.
