# Supplementary material for: Second Generation Sequencing of the Mesothelioma Tumor Genome
Source: PLoS One. 2010 May 13;5(5):e10612. doi: 10.1371/journal.pone.0010612 (PMC2869344; doi:10.1371/journal.pone.0010612)
Supplement: File S1 — (1.11 MB PPT) [file pone.0010612.s005.ppt]

## Slide 1
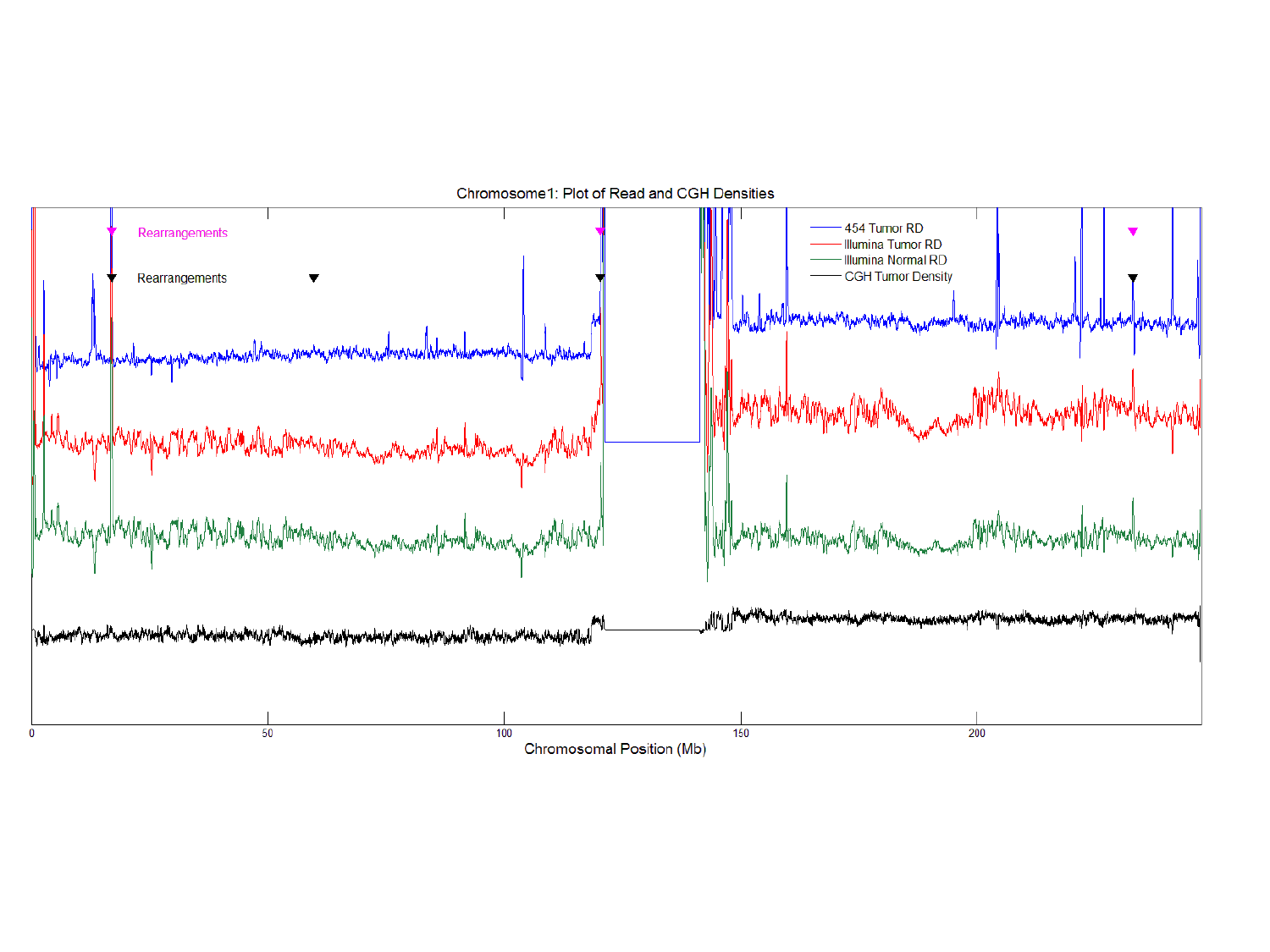

## Slide 2
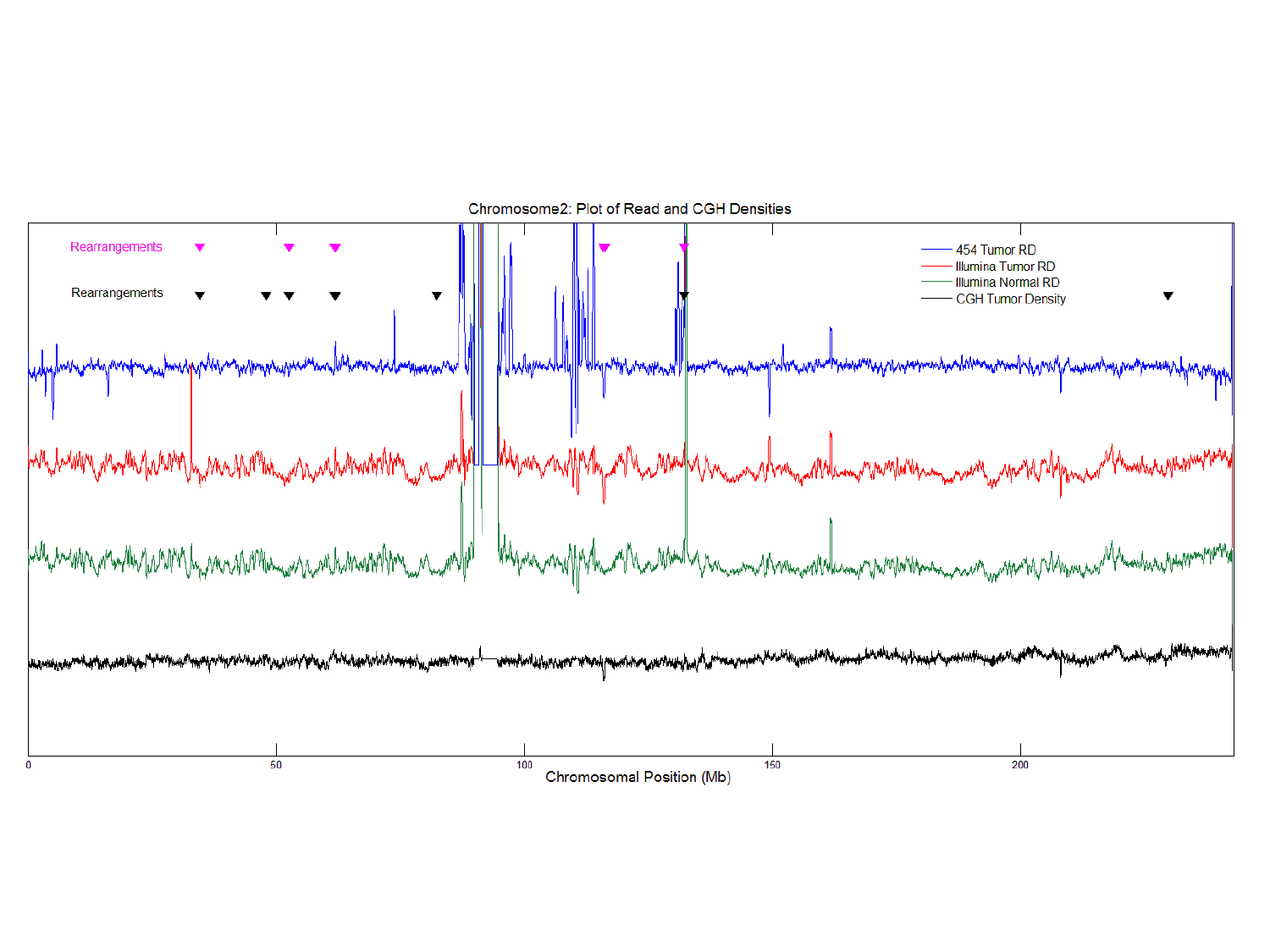

## Slide 3
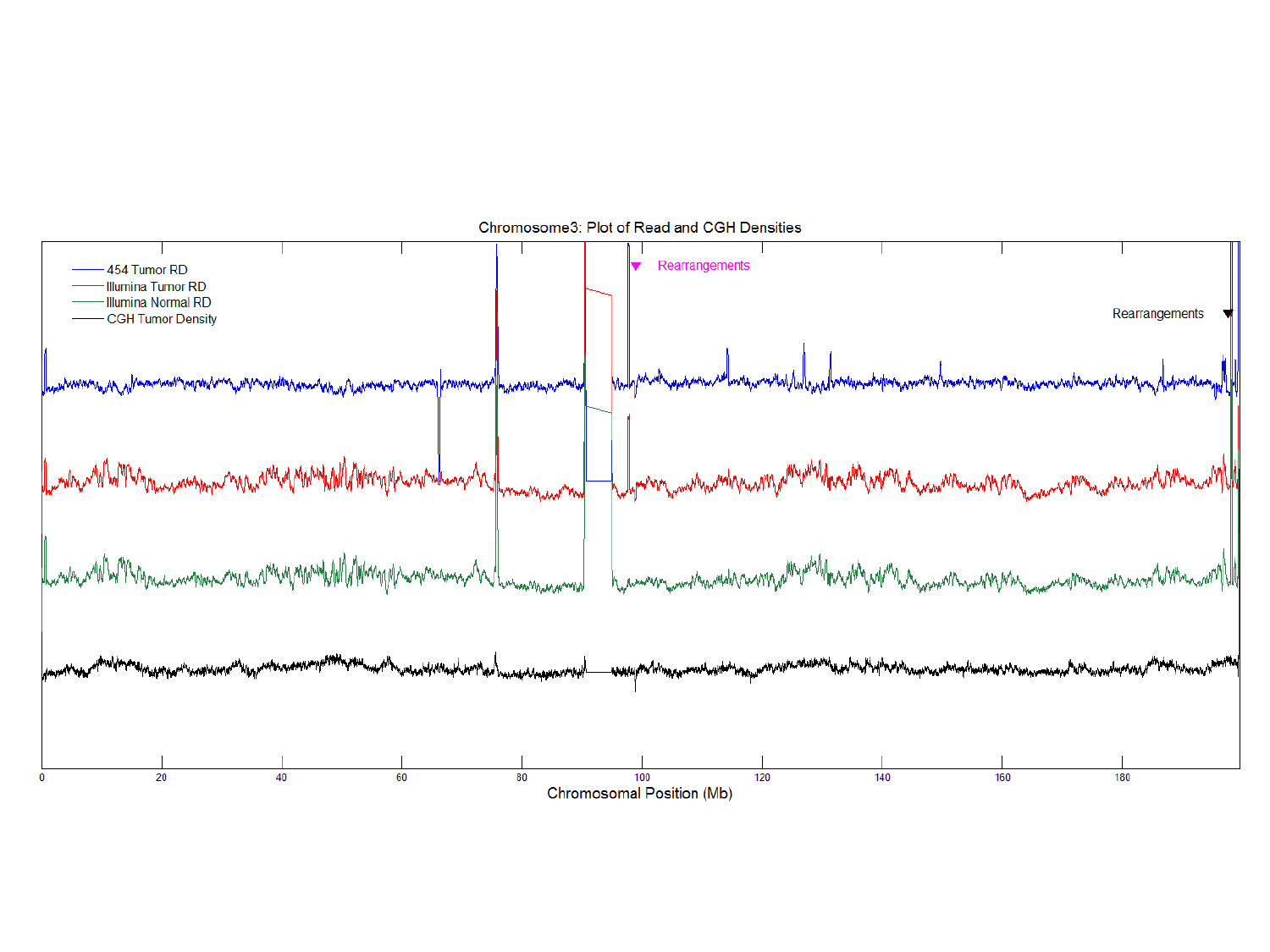

## Slide 4
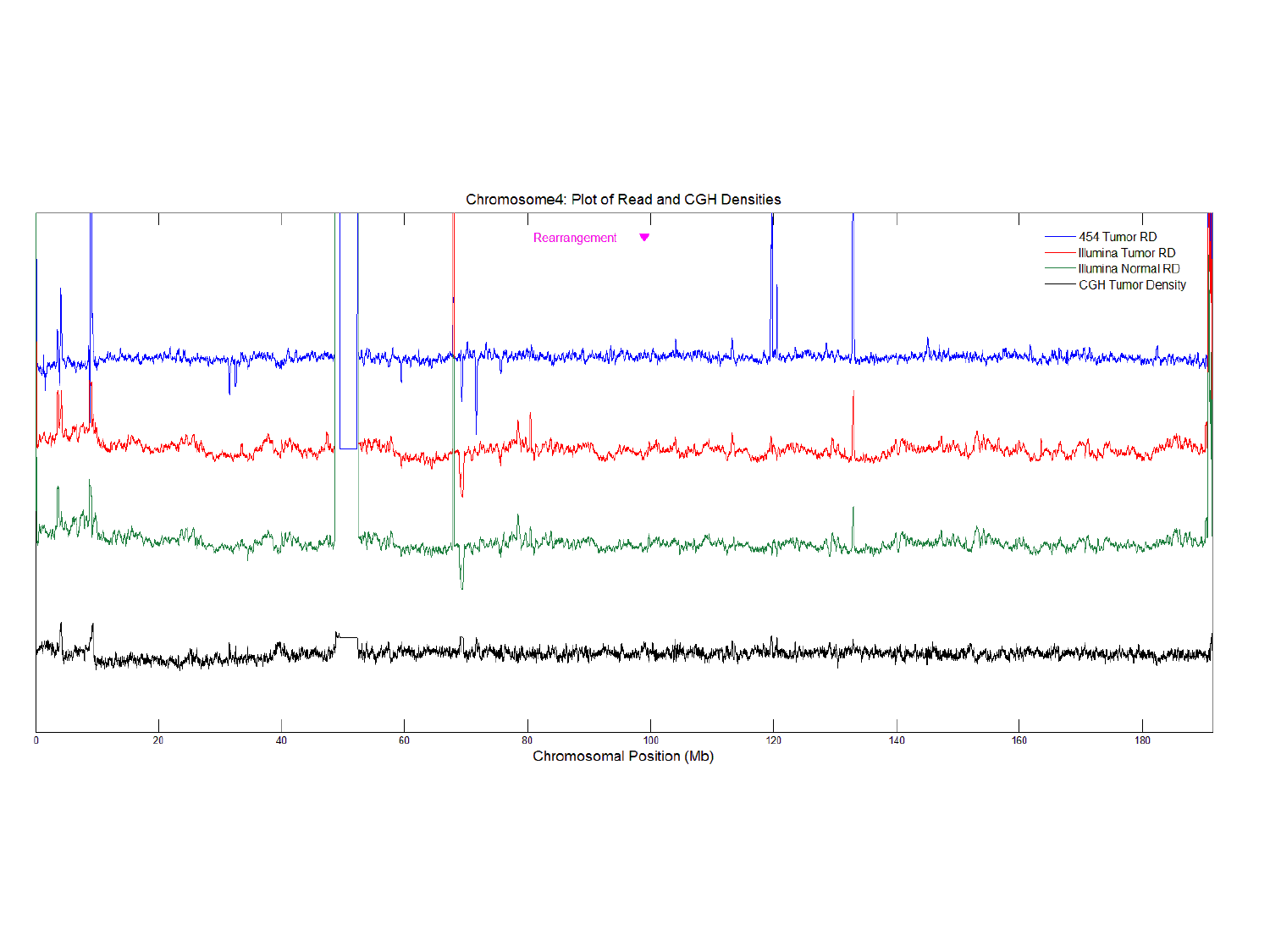

## Slide 5
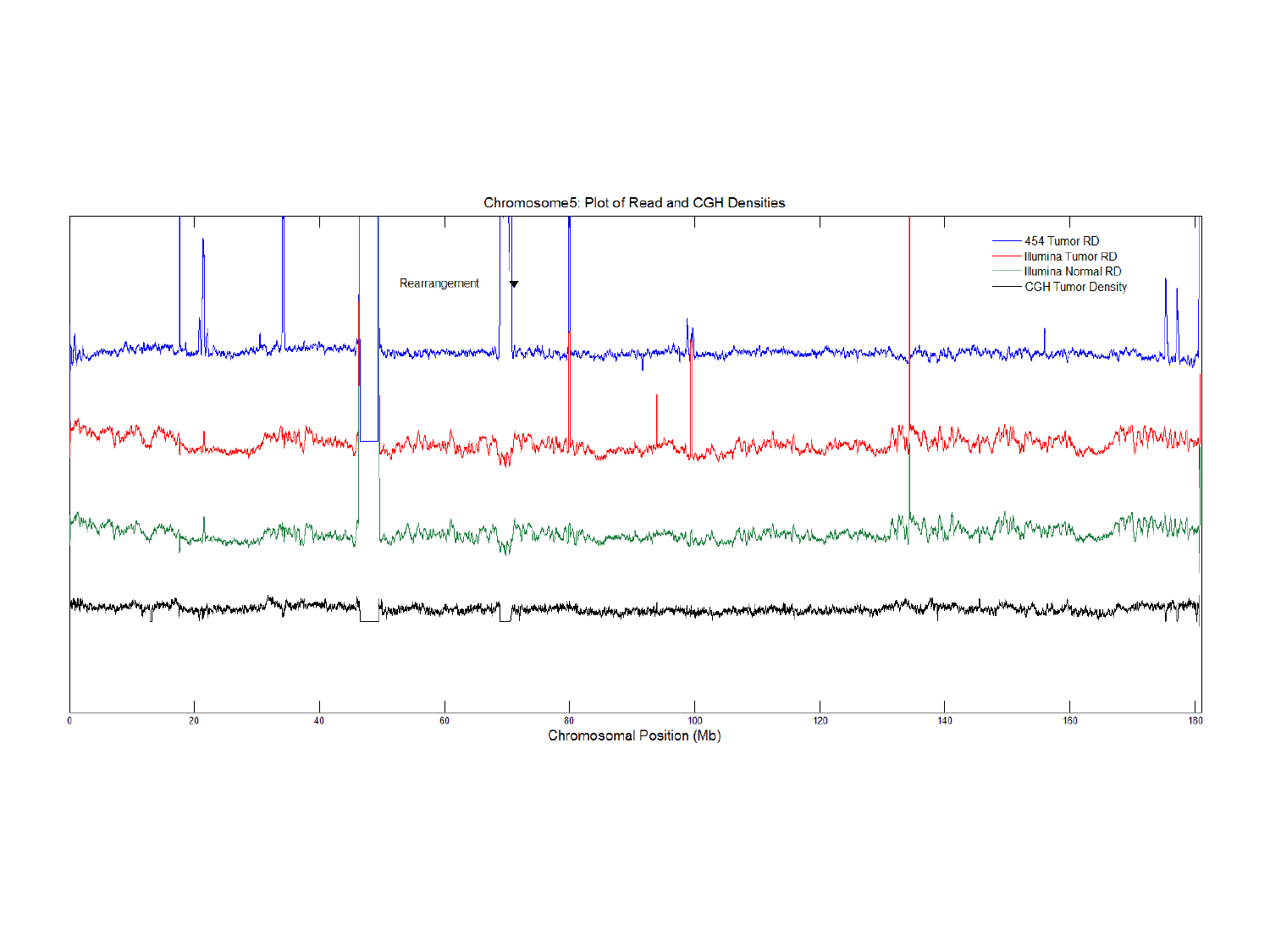

## Slide 6
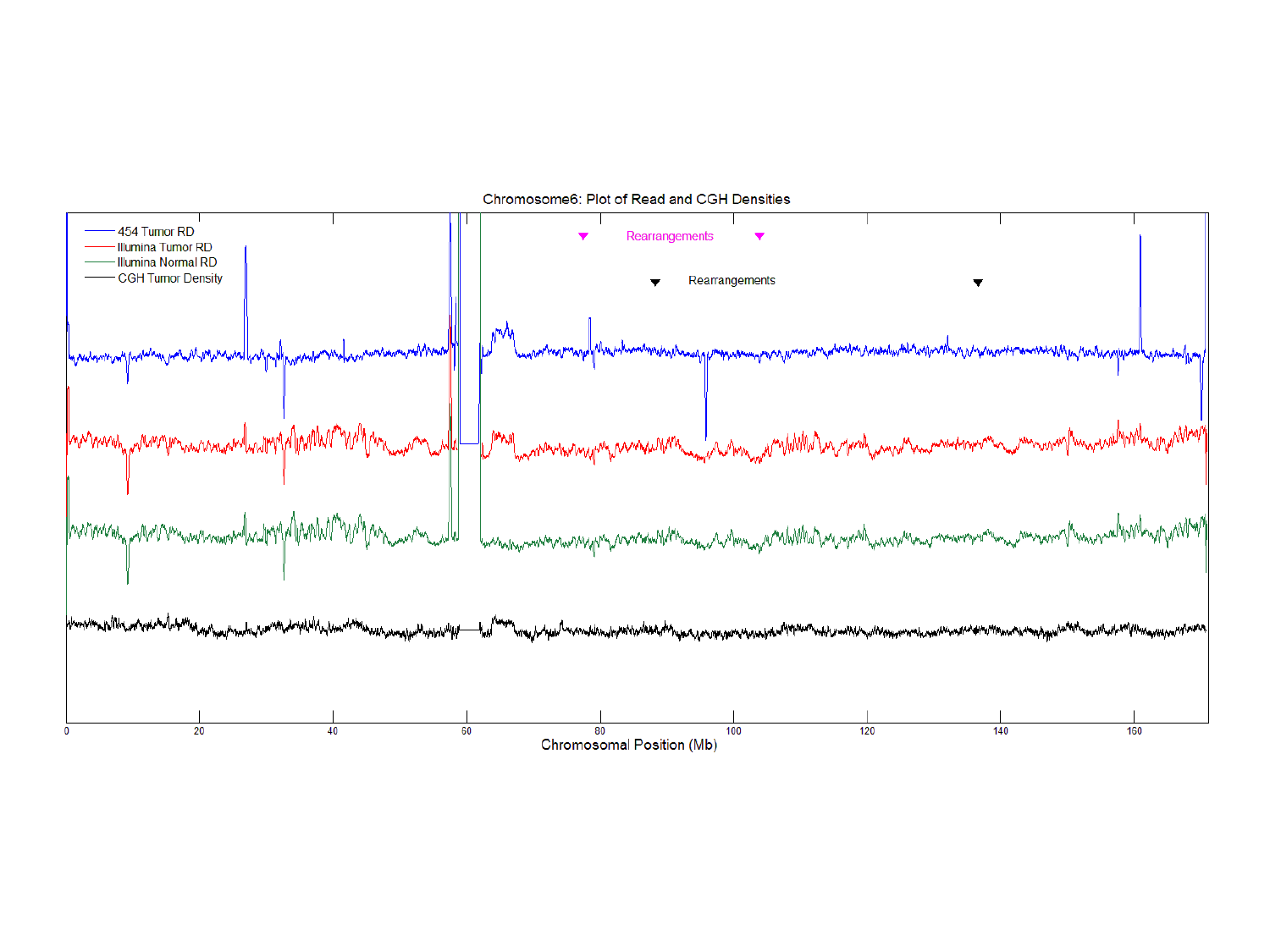

## Slide 7
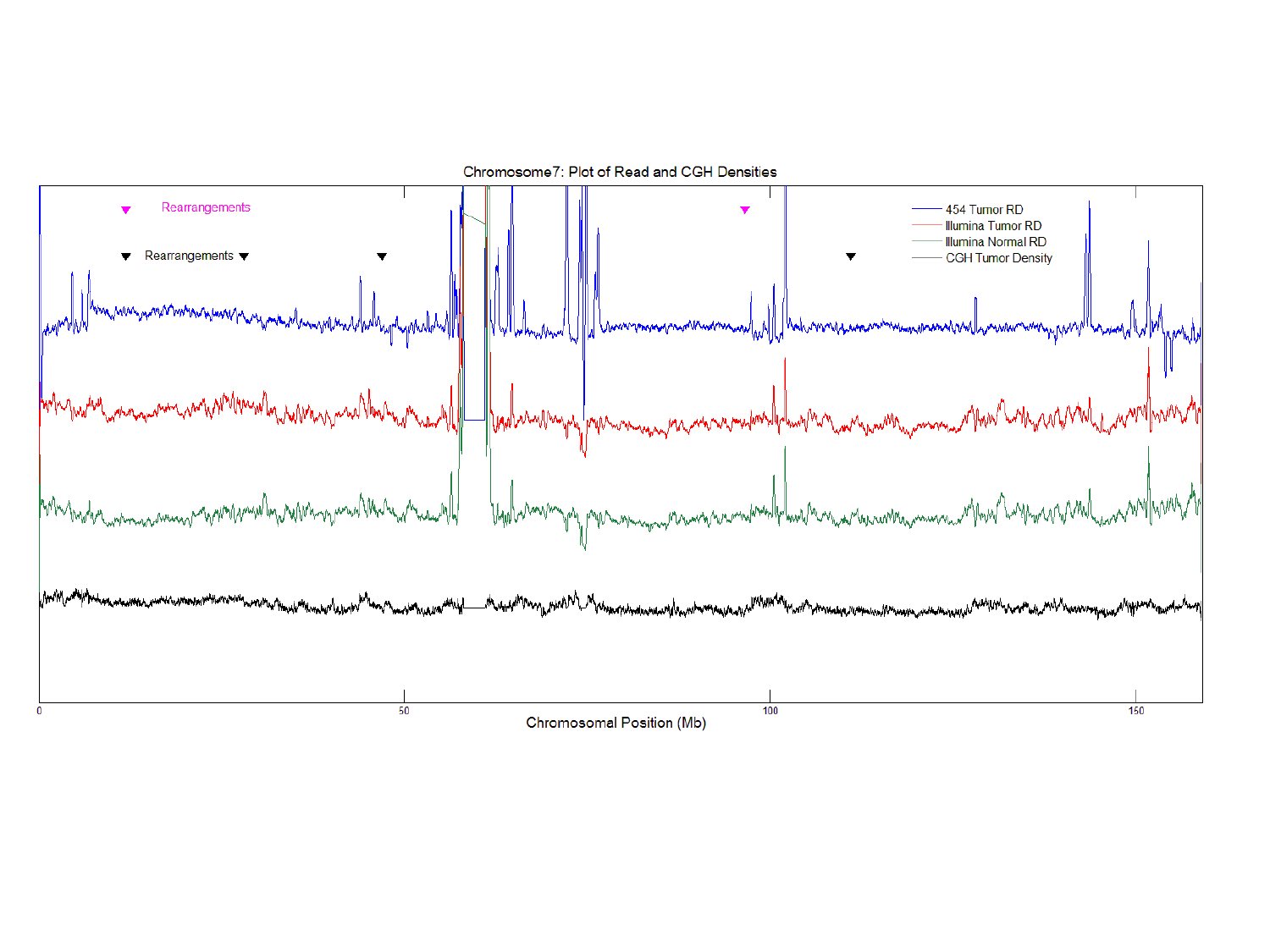

## Slide 8
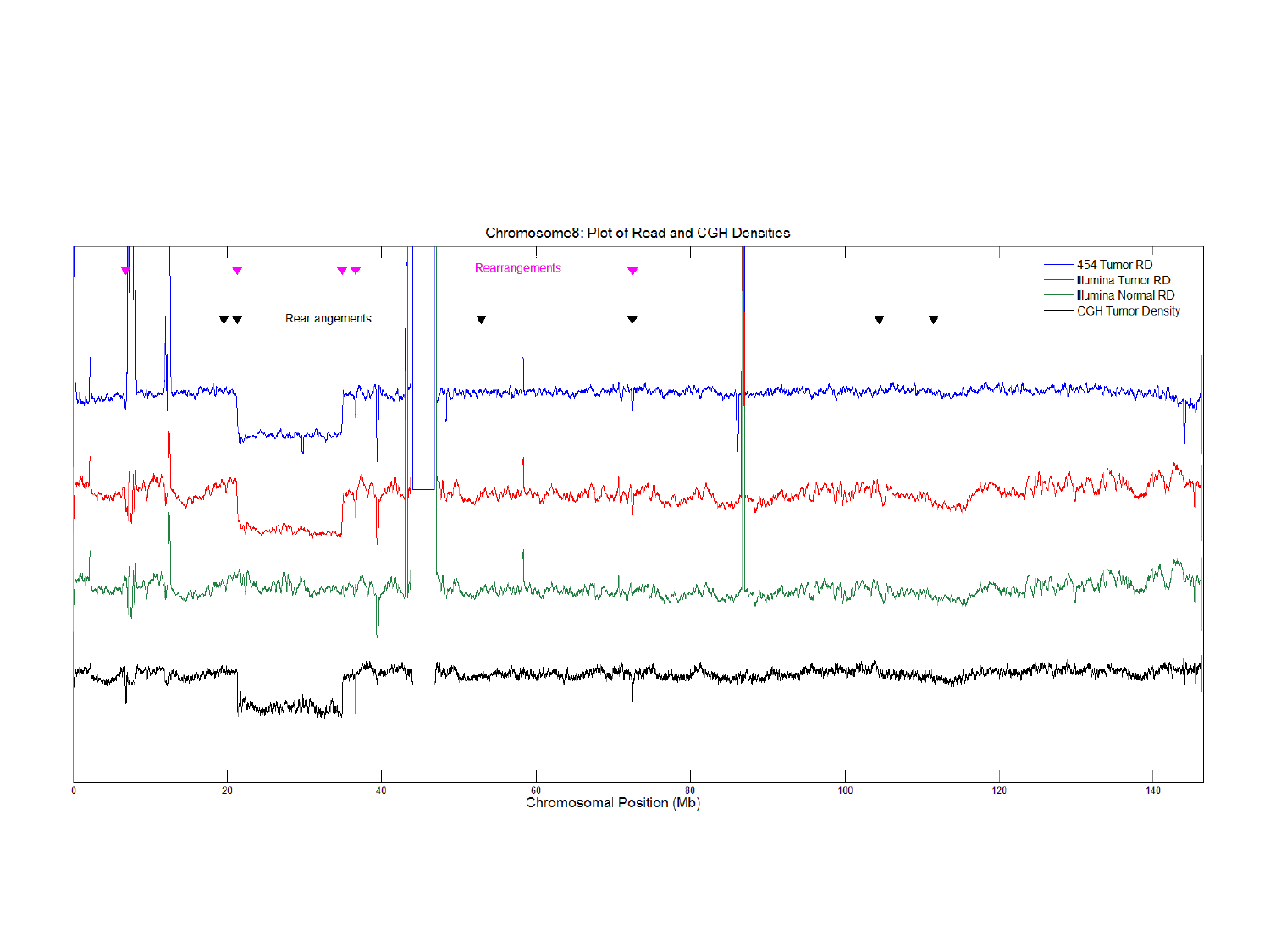

## Slide 9
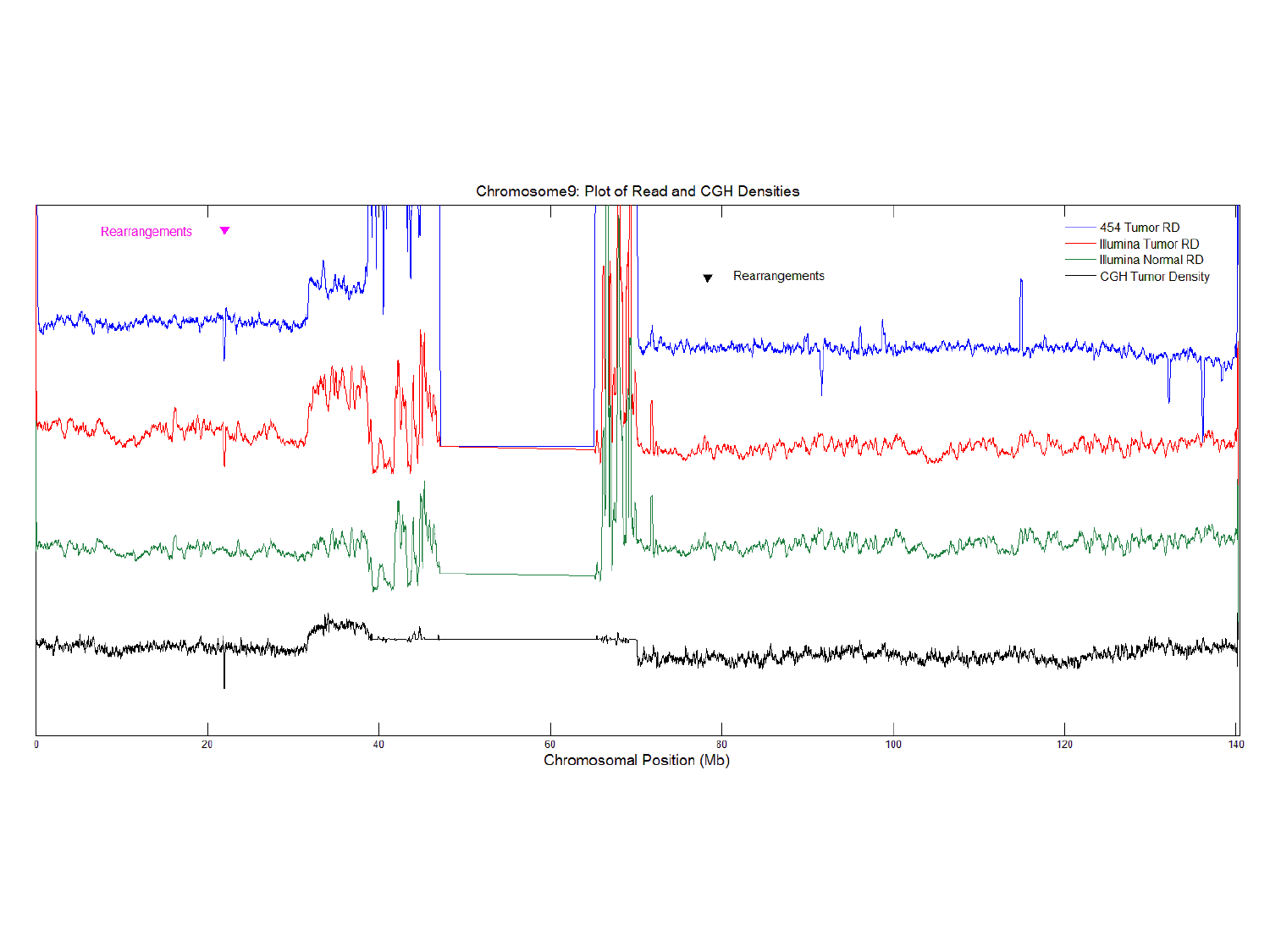

## Slide 10
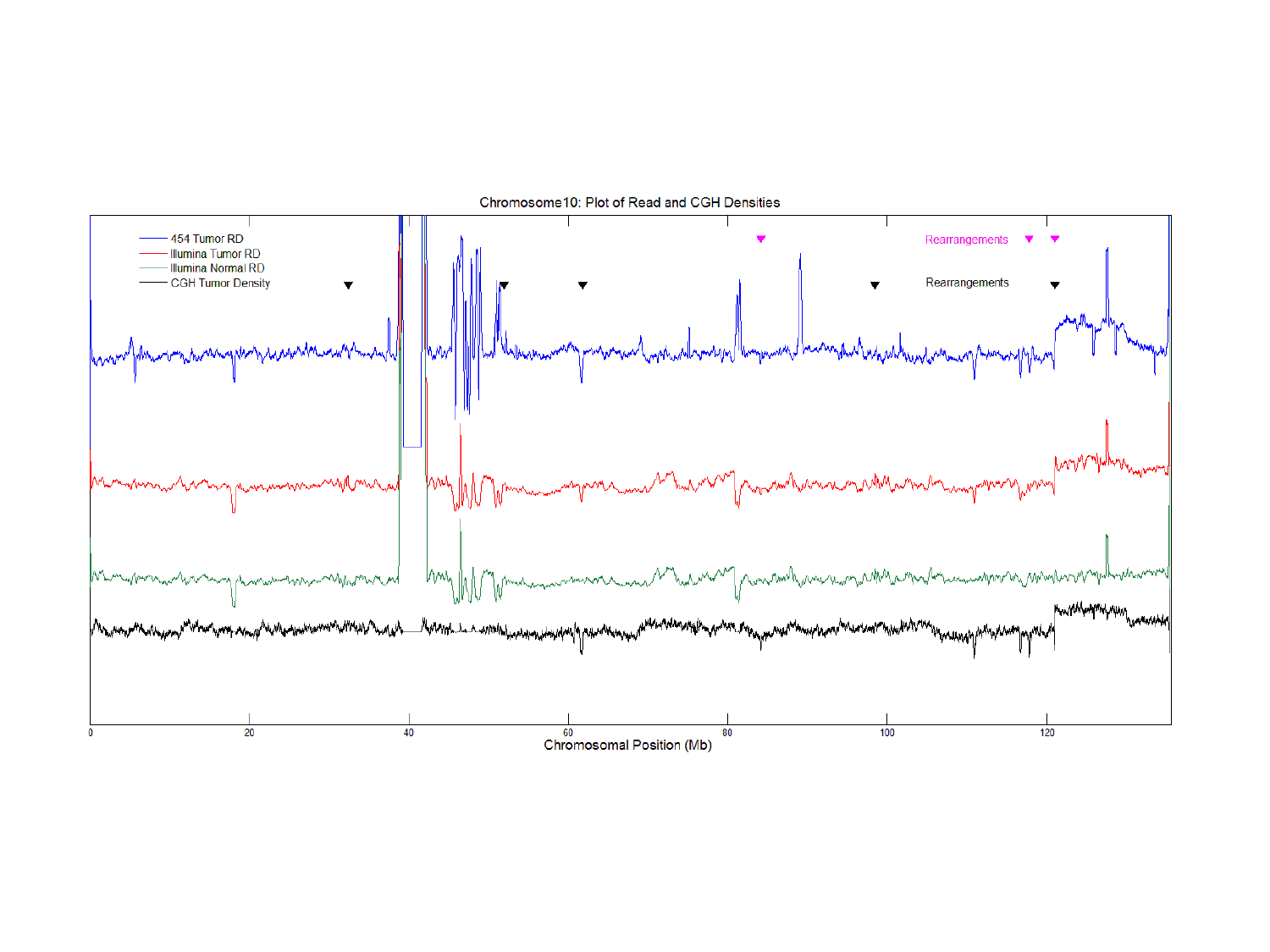

## Slide 11
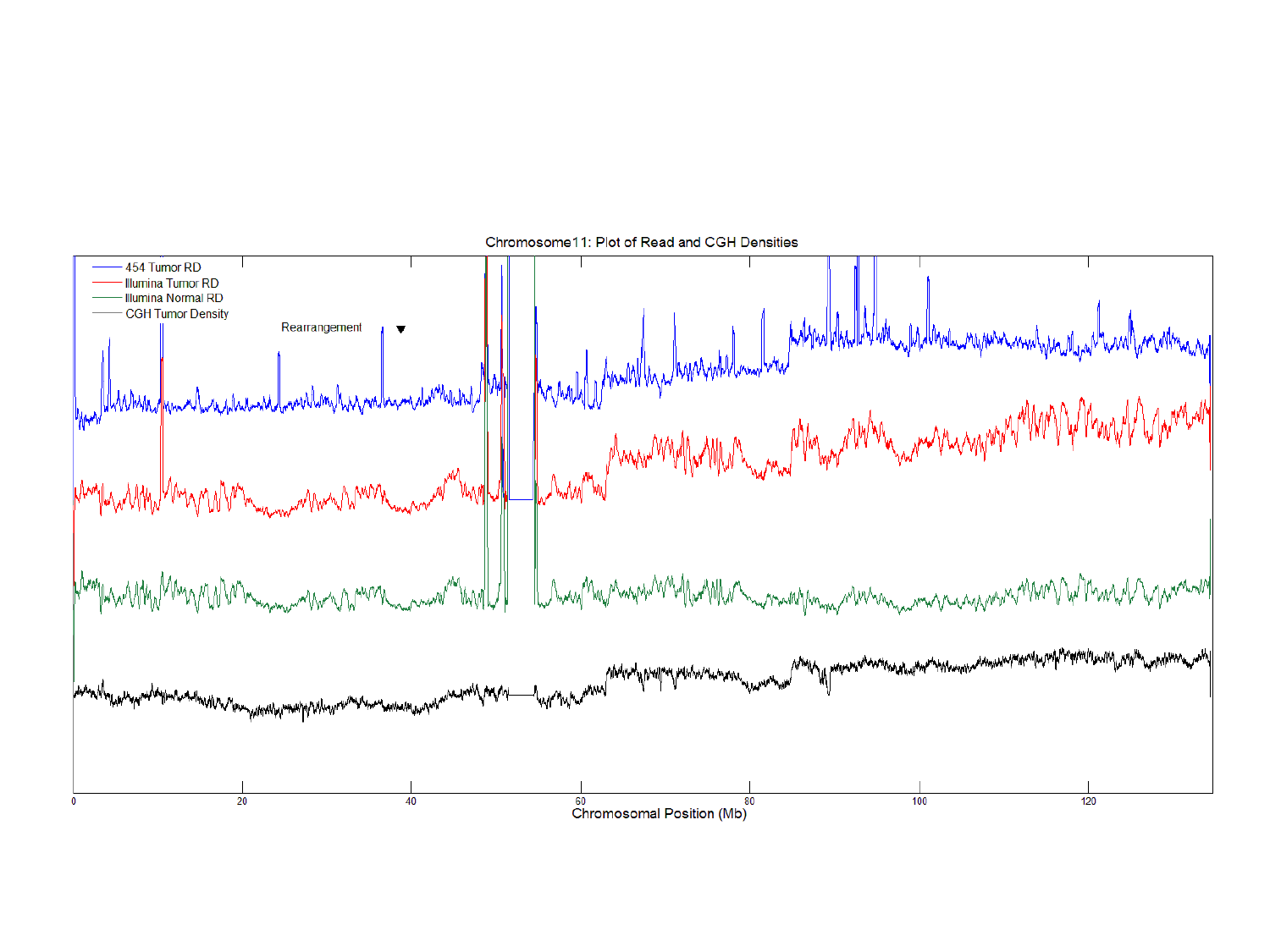

## Slide 12
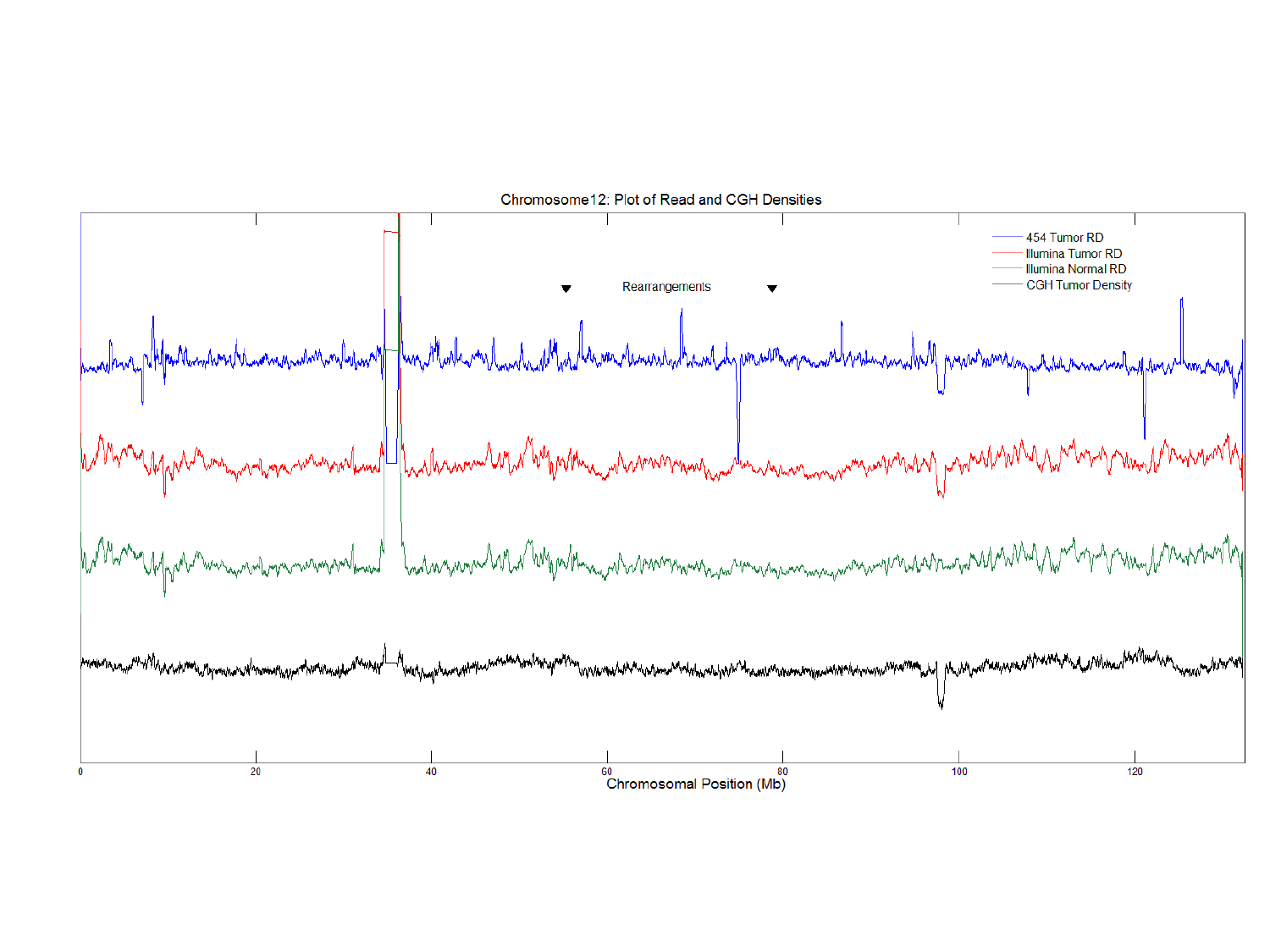

## Slide 13
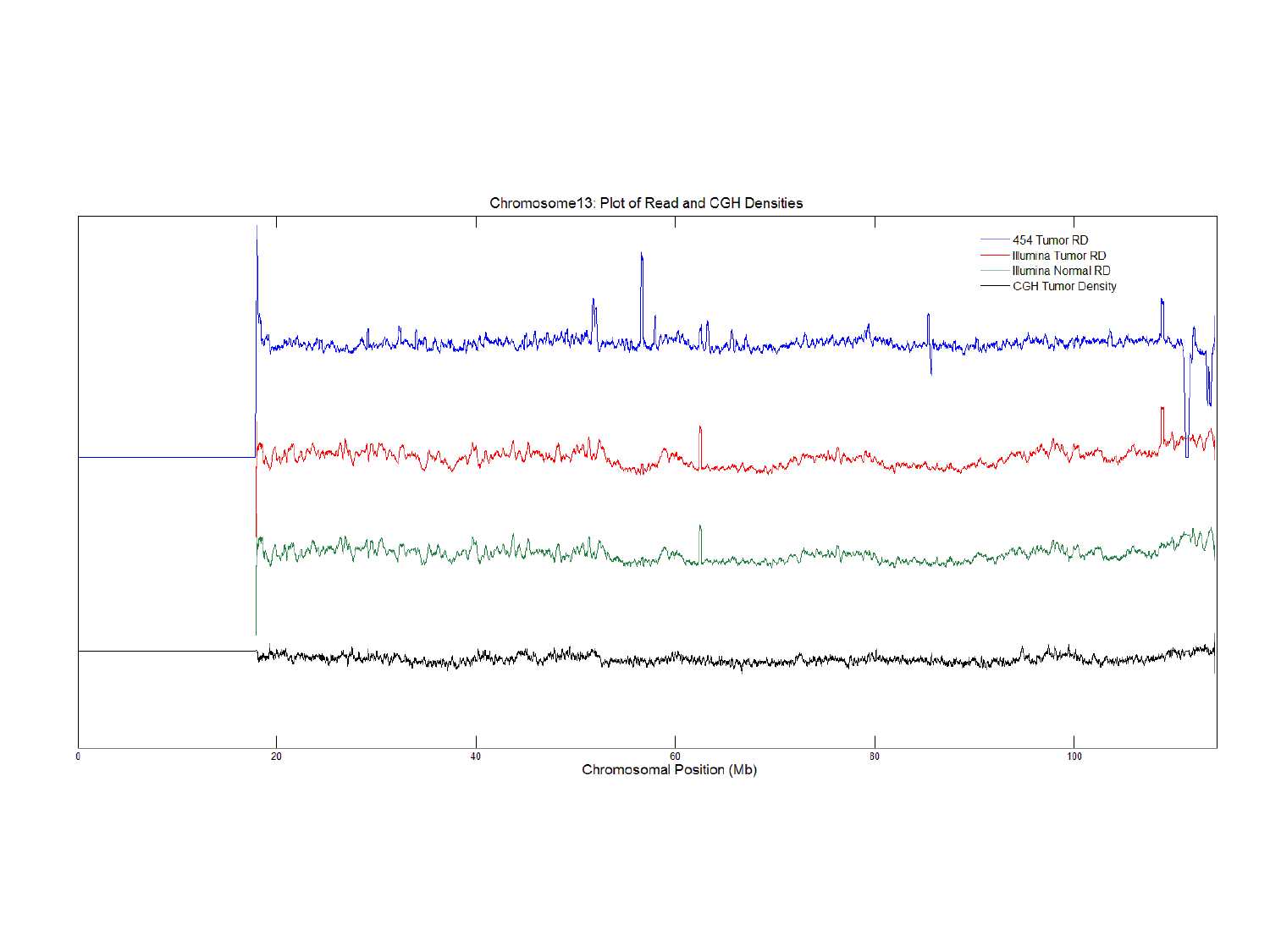

## Slide 14
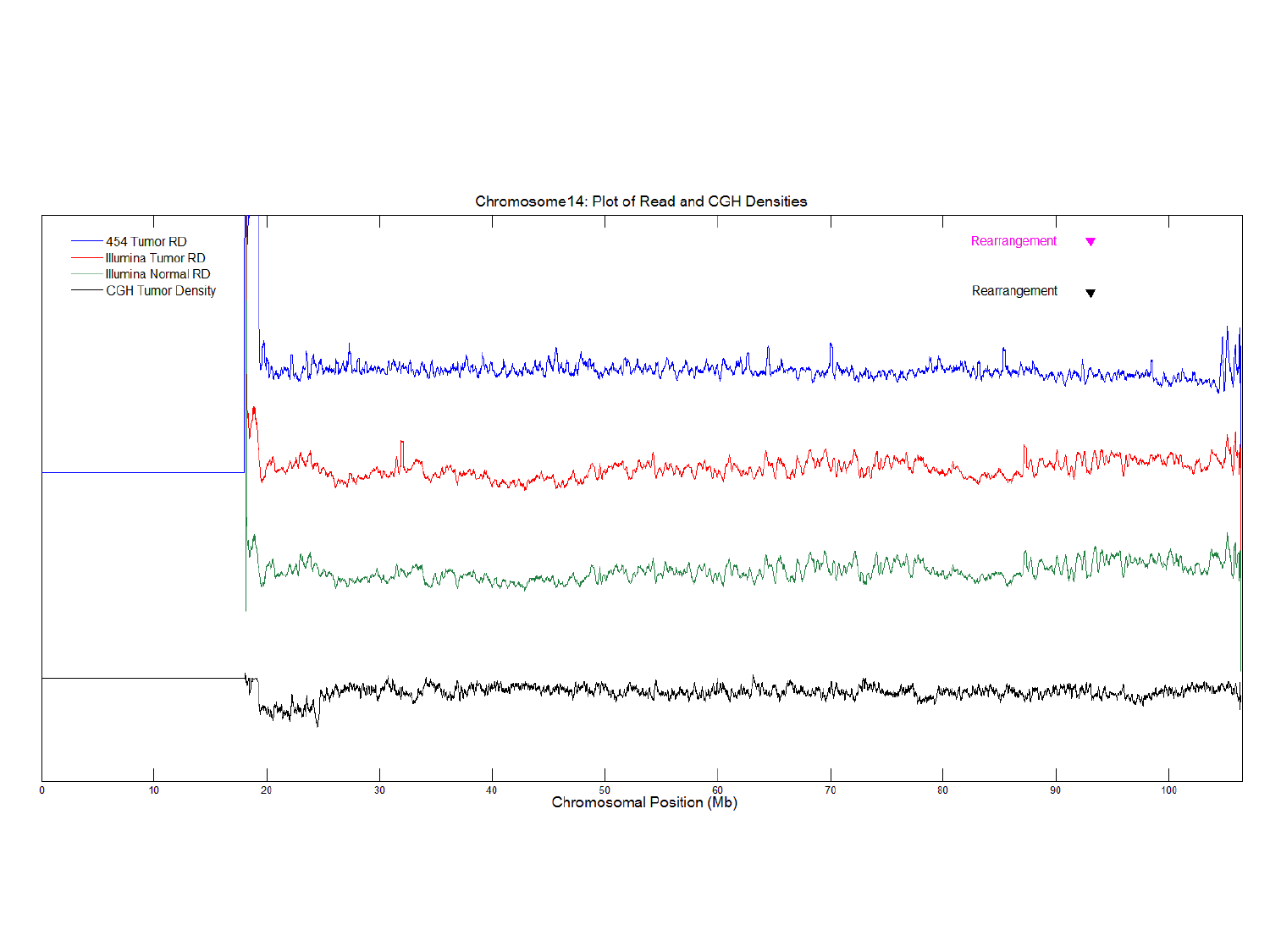

## Slide 15
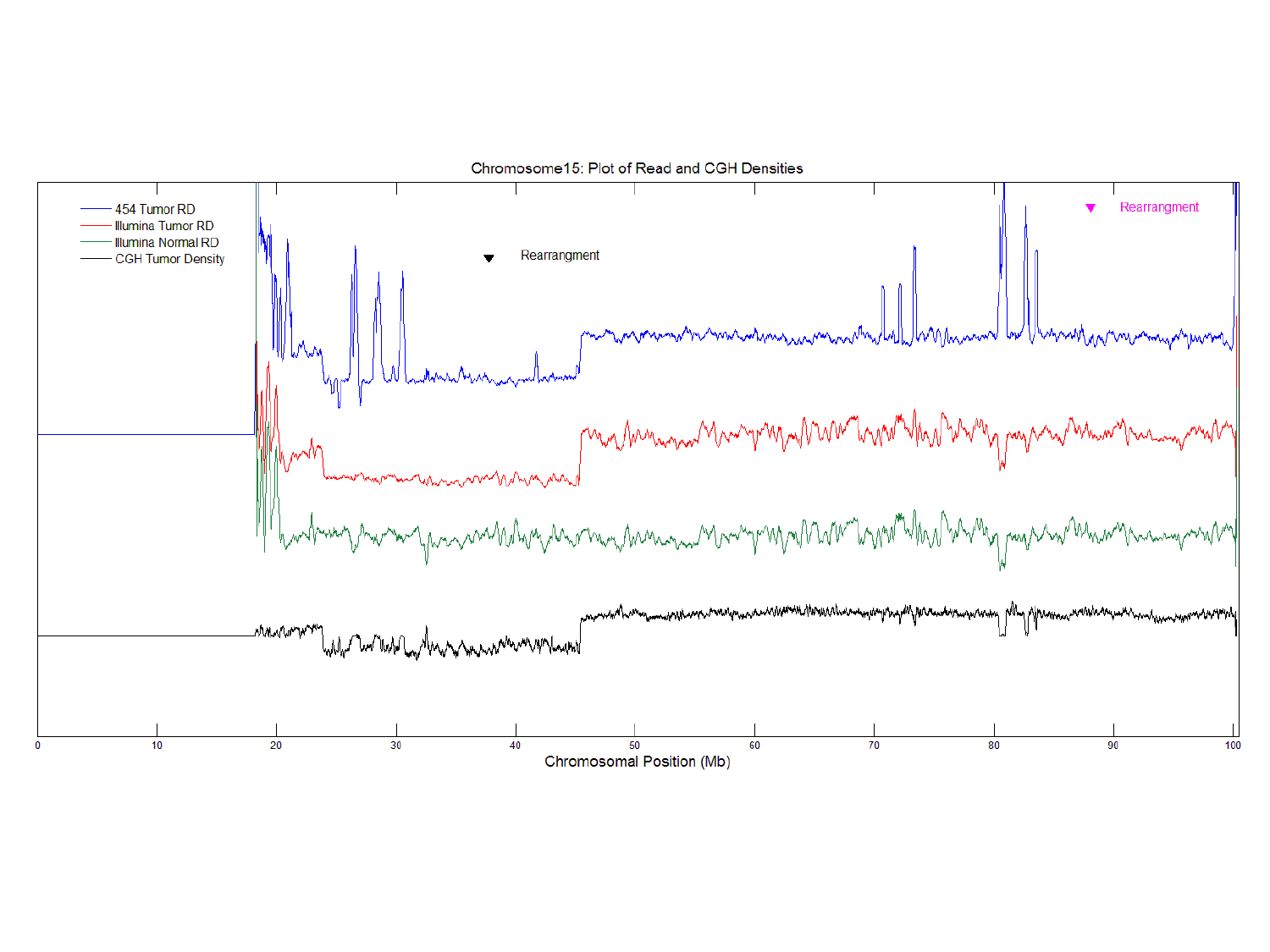

## Slide 16
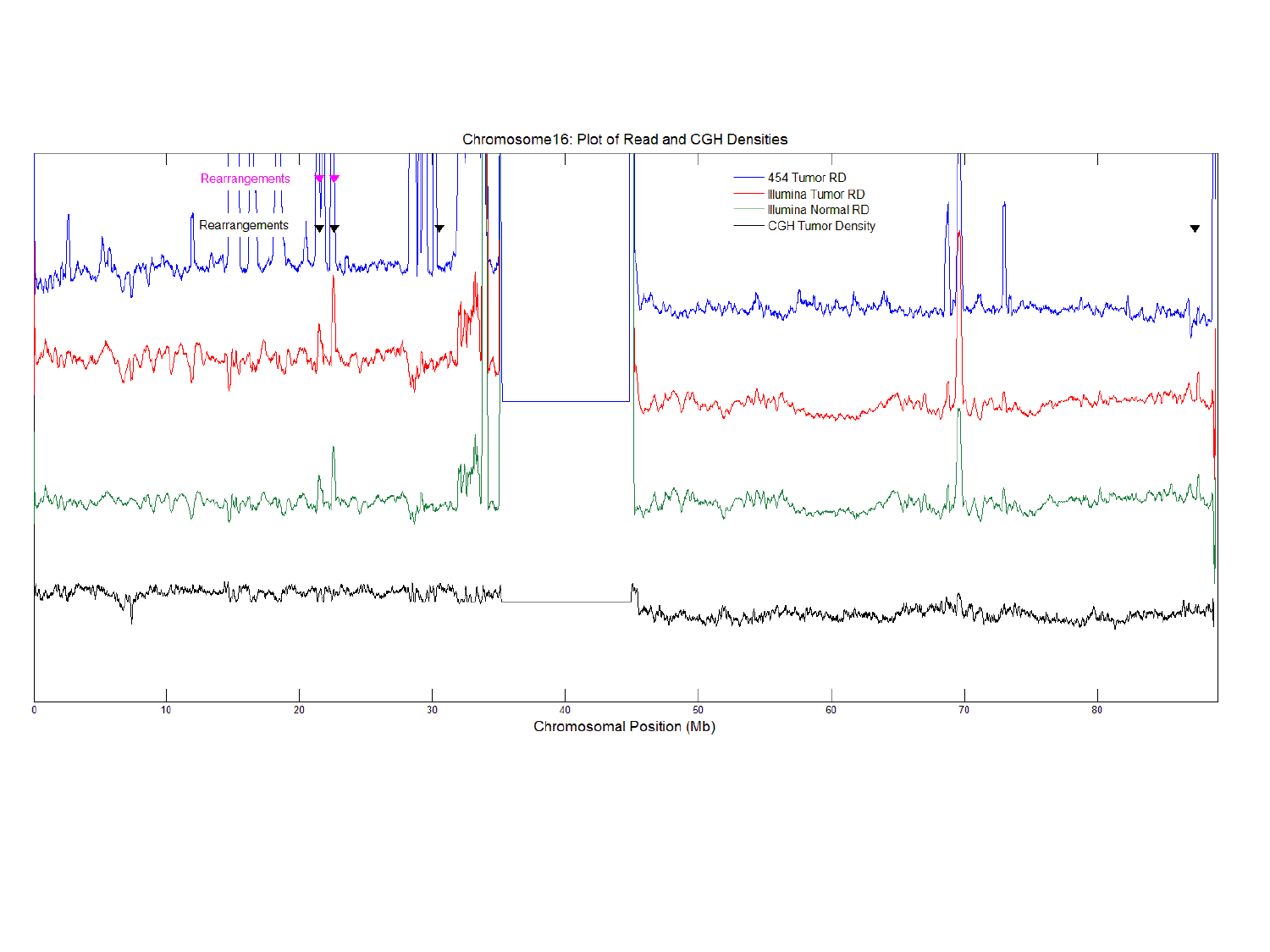

## Slide 17
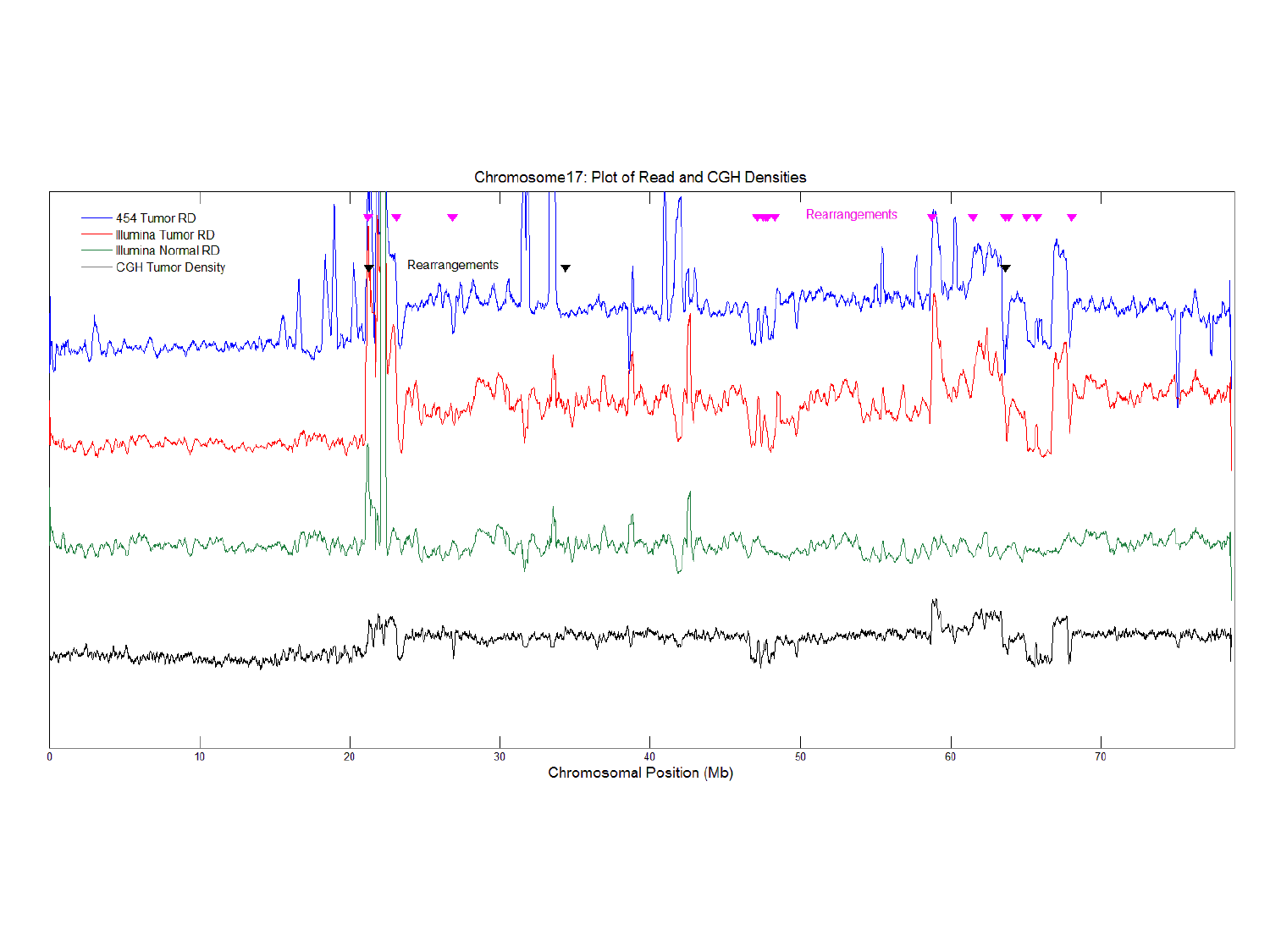

## Slide 18
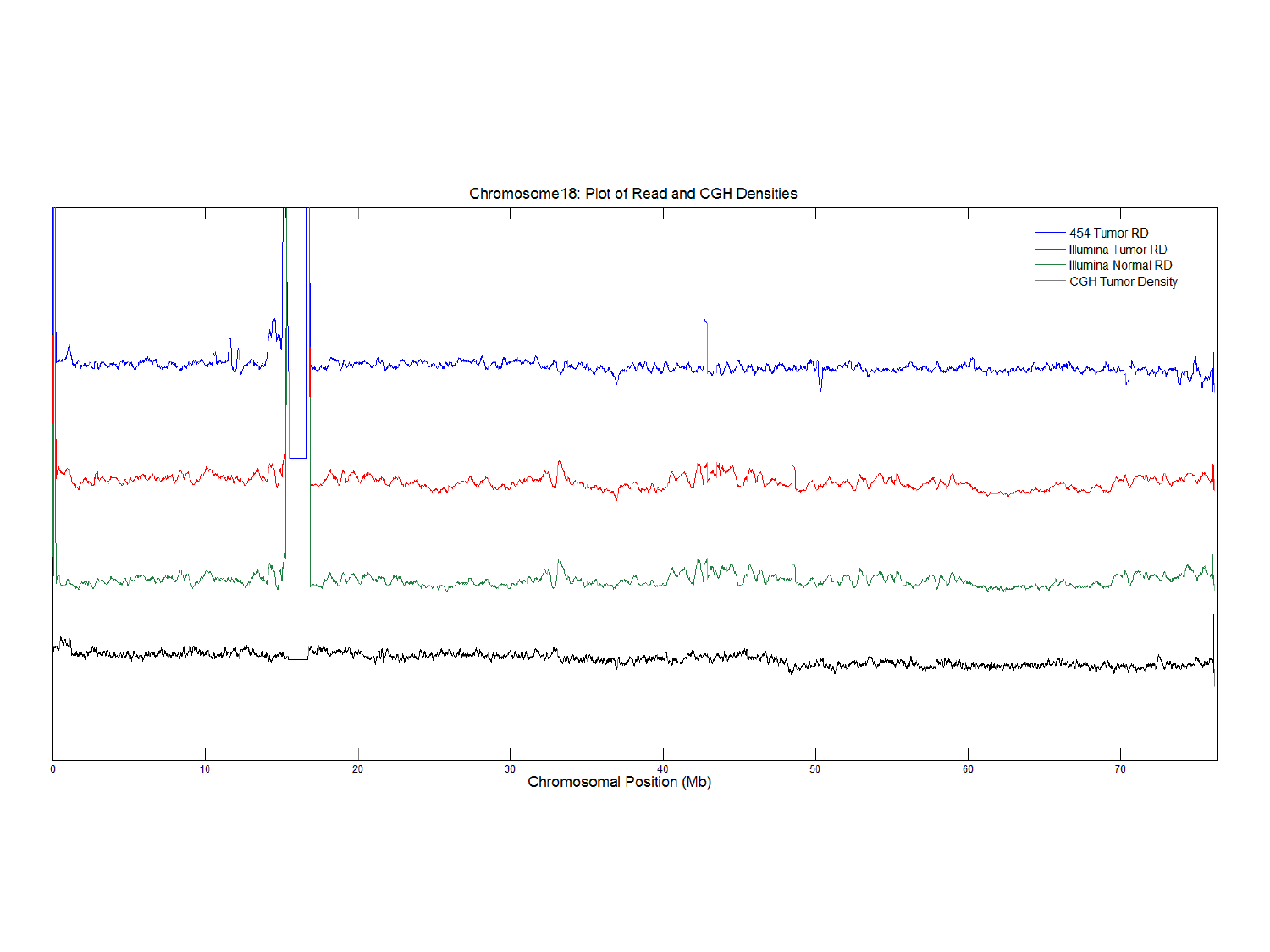

## Slide 19
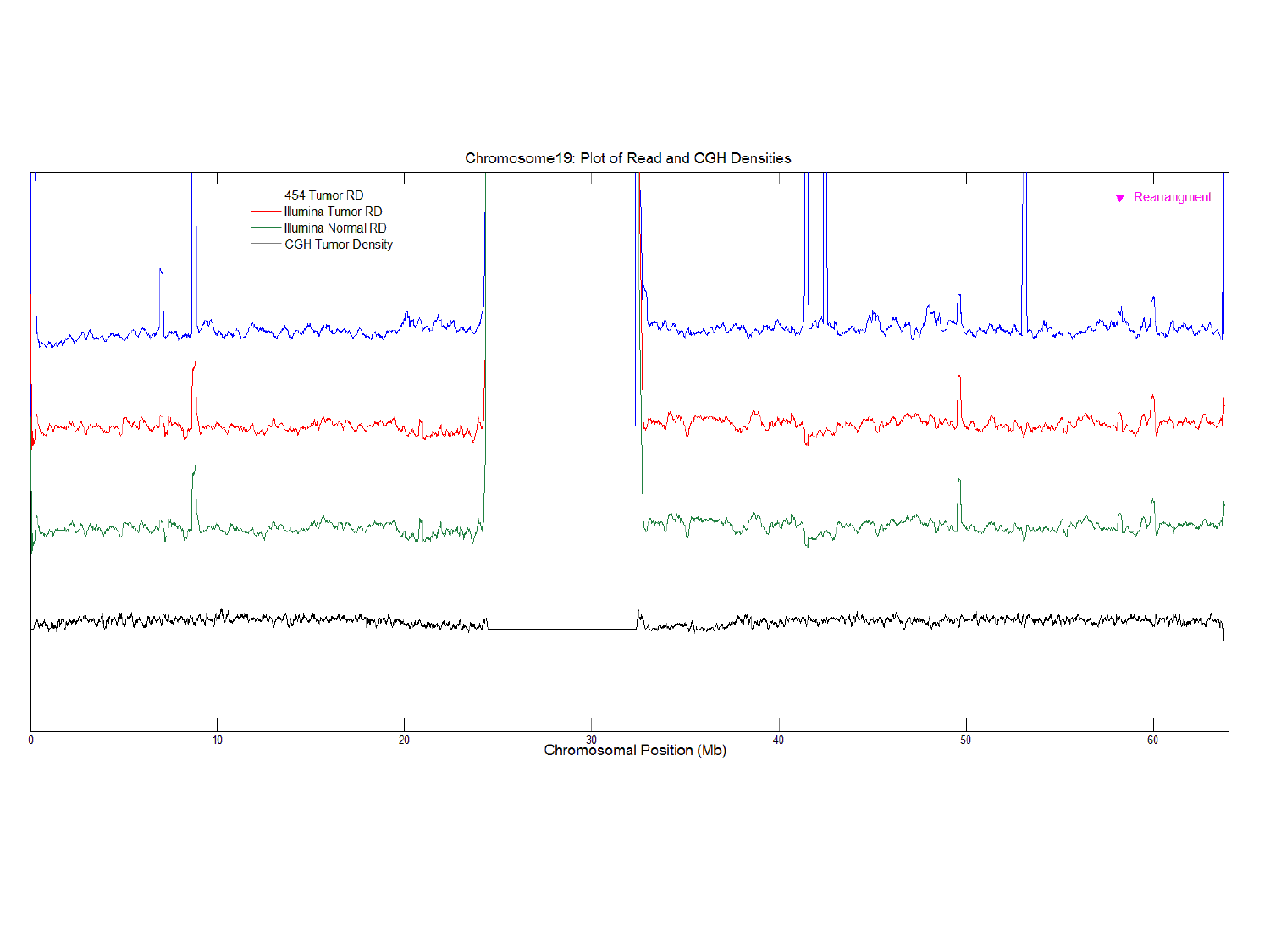

## Slide 20
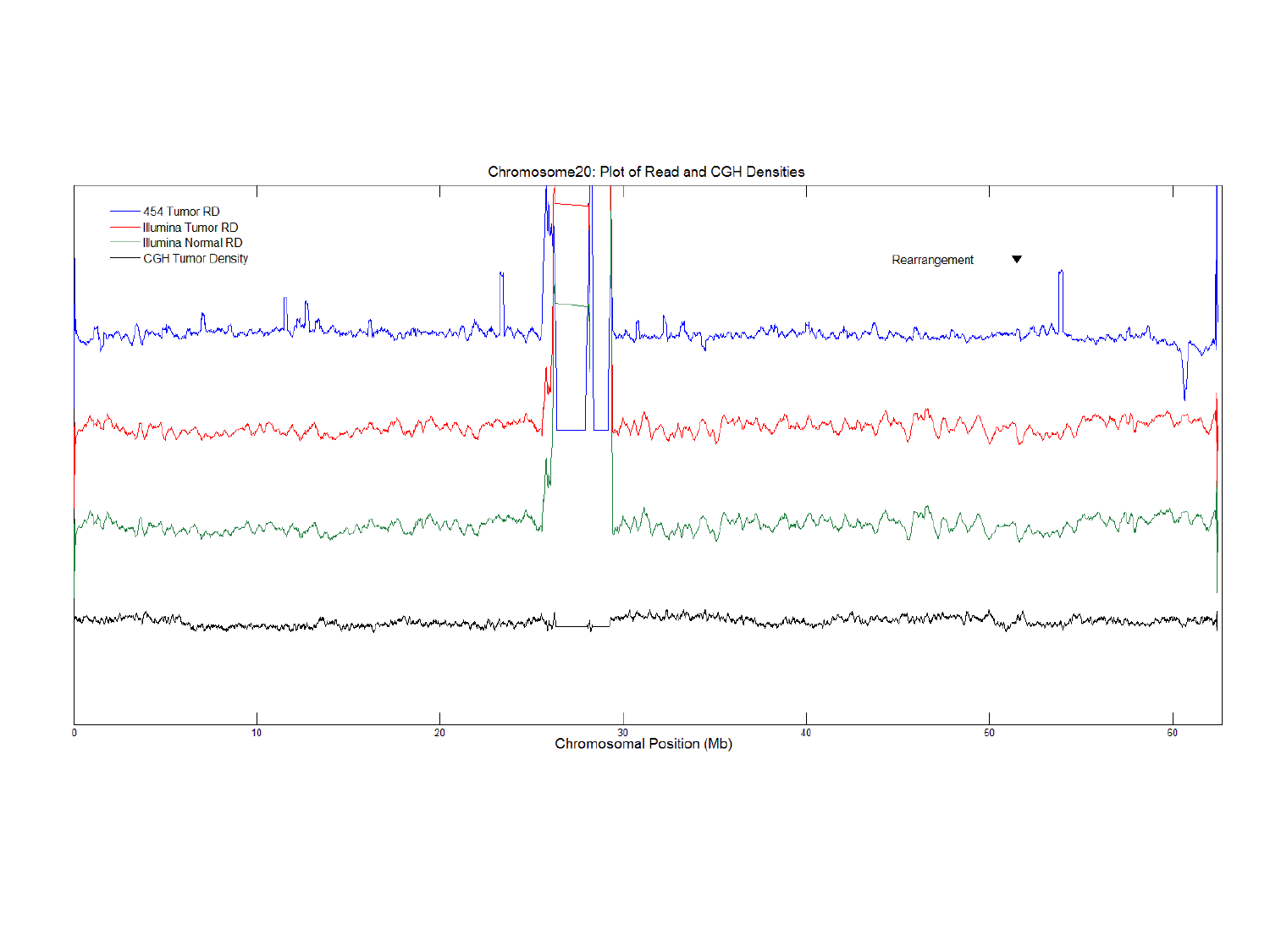

## Slide 21
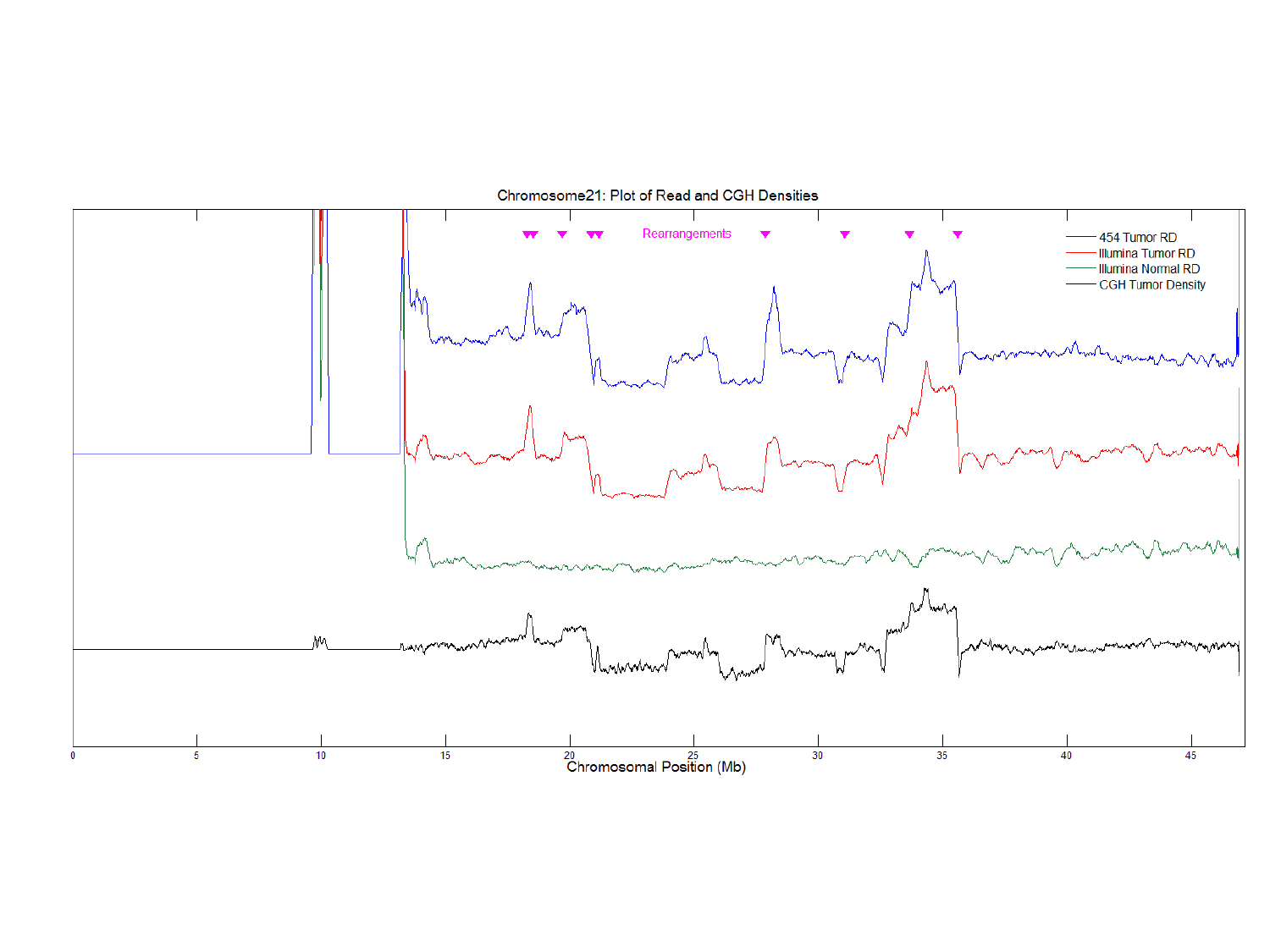

## Slide 22
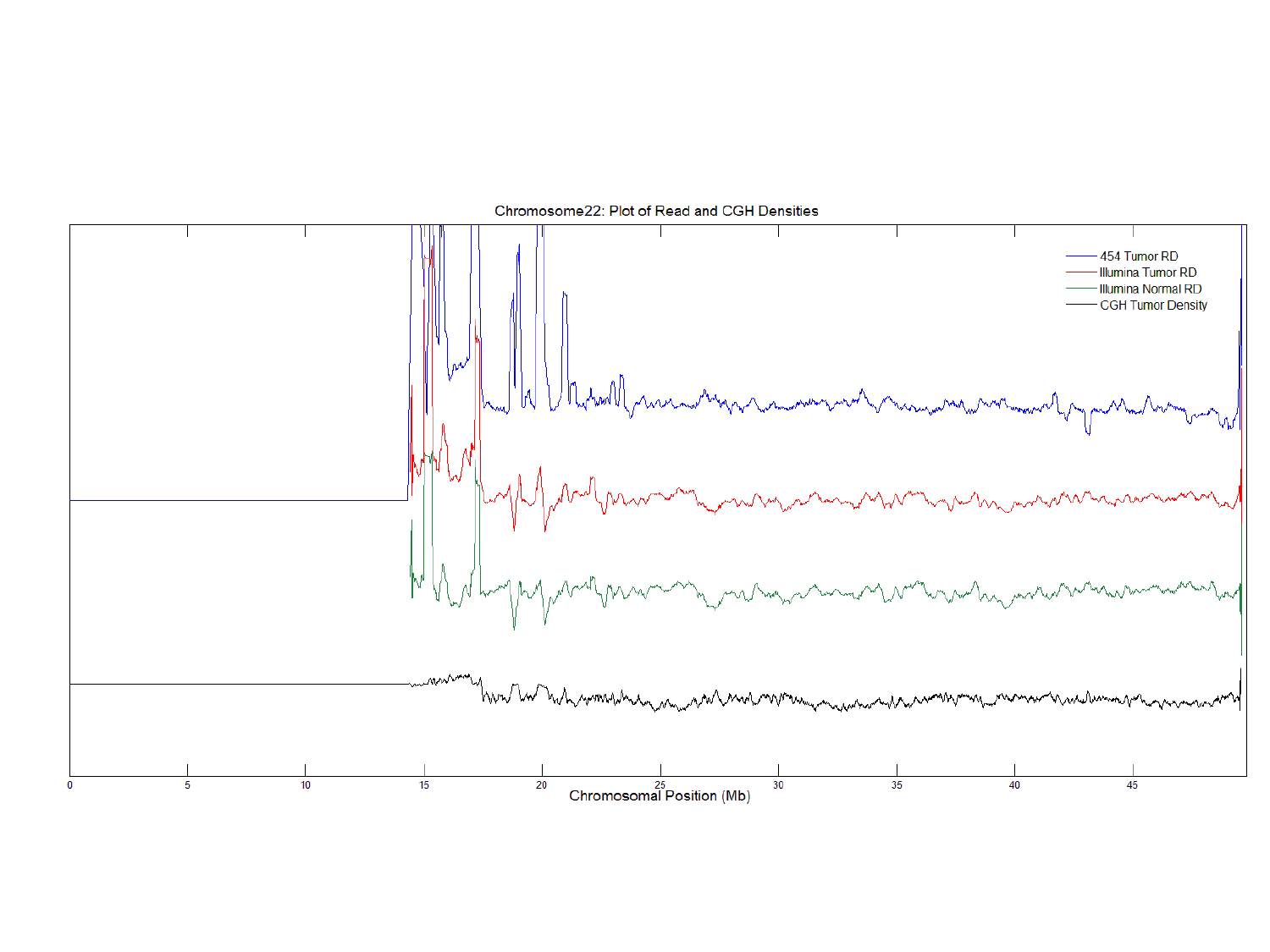

## Slide 23
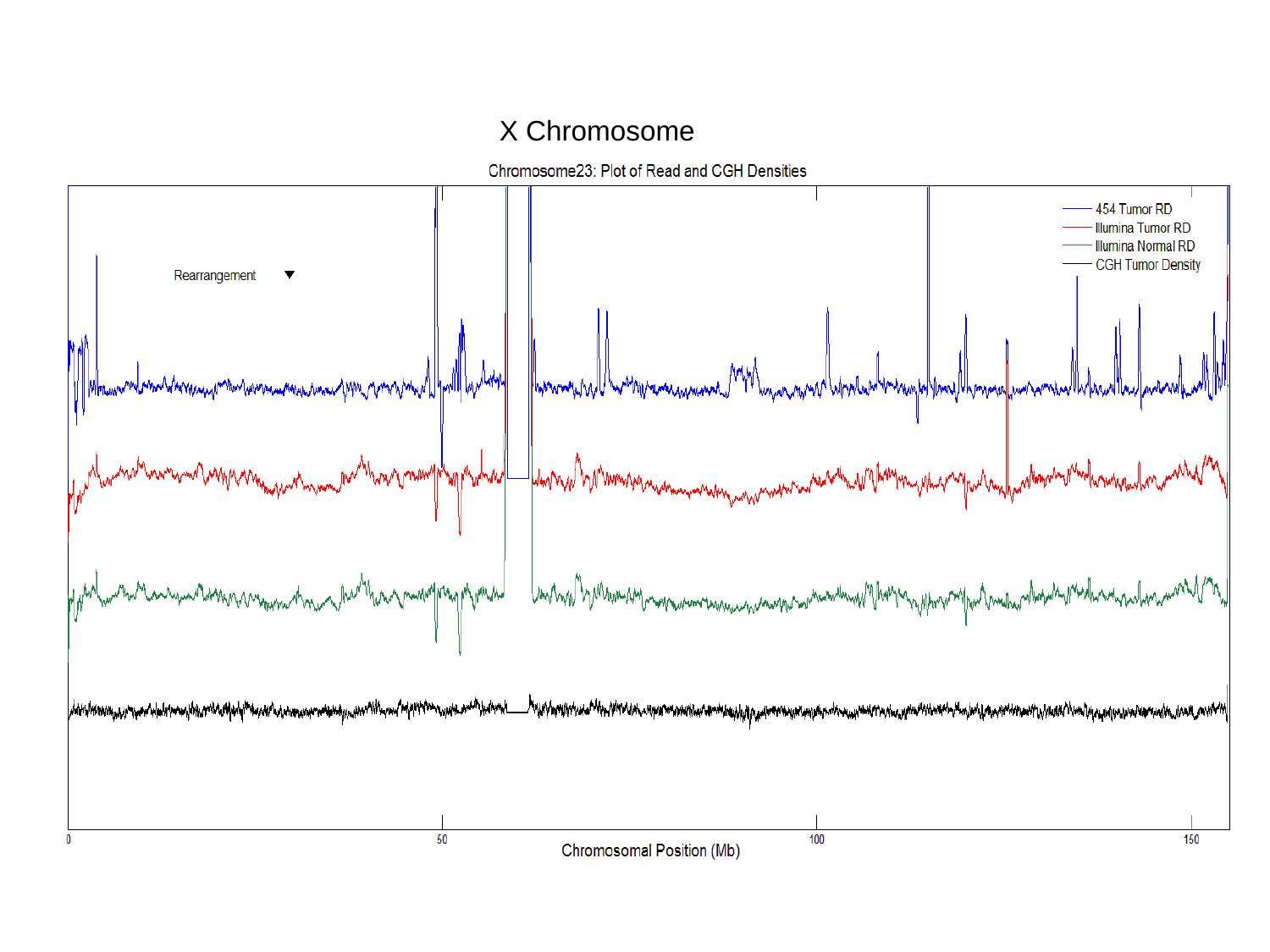

X Chromosome

## Slide 24
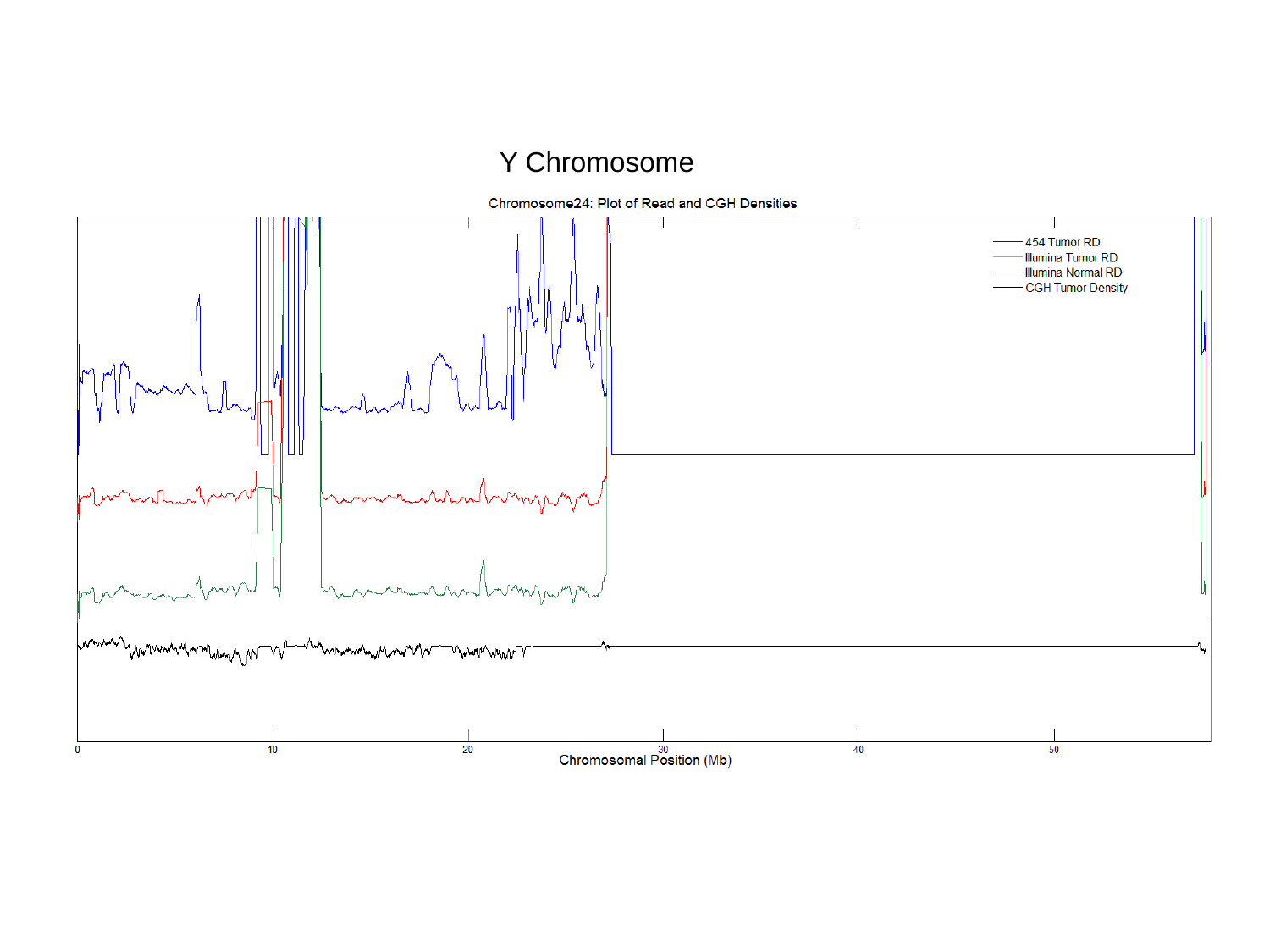

Y Chromosome
