# Supplementary material for: Second Generation Sequencing of the Mesothelioma Tumor Genome
Source: PLoS One. 2010 May 13;5(5):e10612. doi: 10.1371/journal.pone.0010612 (PMC2869344; doi:10.1371/journal.pone.0010612)
Supplement: File S2 — Direction and chromosomal locations of the rearrangement. (1.79 MB PPT) [file pone.0010612.s006.ppt]

## Slide 1
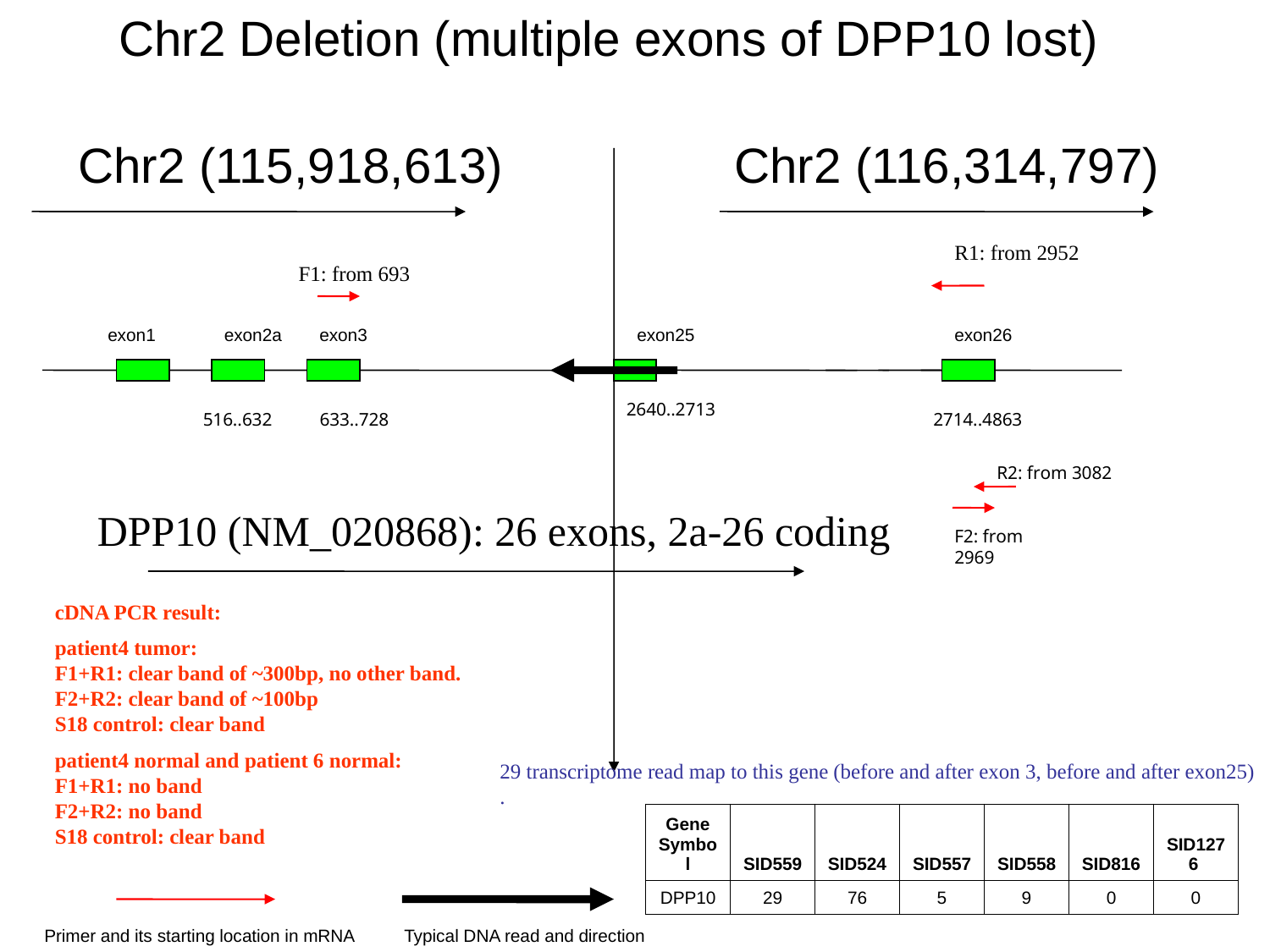

Chr2 Deletion (multiple exons of DPP10 lost)
Chr2 (115,918,613)
Chr2 (116,314,797)
R1: from 2952
F1: from 693
exon1
exon2a
exon3
exon25
exon26
2640..2713
516..632
633..728
2714..4863
R2: from 3082
DPP10 (NM_020868): 26 exons, 2a-26 coding
| |
| --- |
F2: from 2969
| |
| --- |
cDNA PCR result:
patient4 tumor:
F1+R1: clear band of ~300bp, no other band.
F2+R2: clear band of ~100bp
S18 control: clear band
patient4 normal and patient 6 normal:
F1+R1: no band
F2+R2: no band
S18 control: clear band
29 transcriptome read map to this gene (before and after exon 3, before and after exon25)
.
| Gene Symbol | SID559 | SID524 | SID557 | SID558 | SID816 | SID1276 |
| --- | --- | --- | --- | --- | --- | --- |
| DPP10 | 29 | 76 | 5 | 9 | 0 | 0 |
Primer and its starting location in mRNA
Typical DNA read and direction

## Slide 2
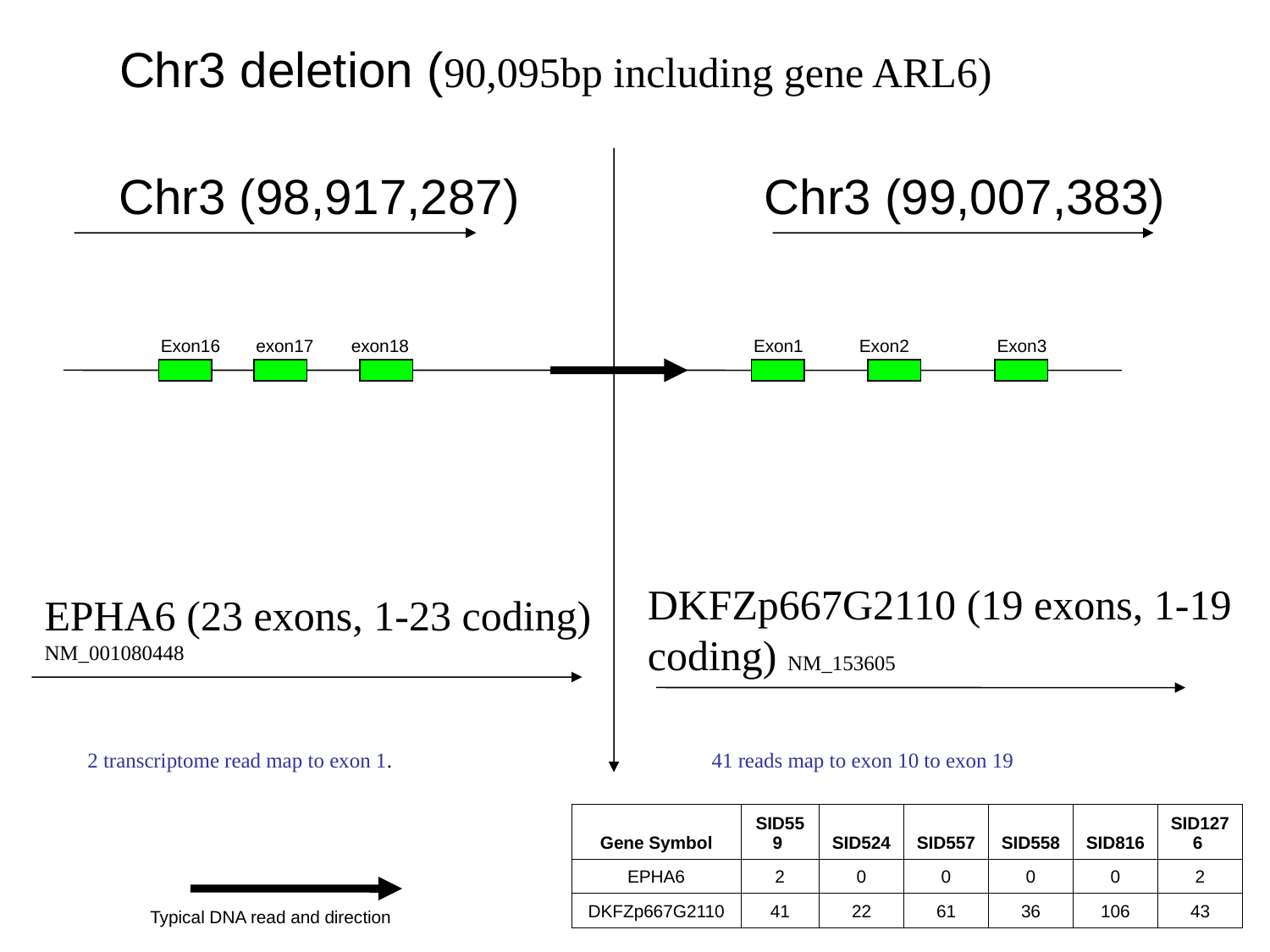

Chr3 deletion (90,095bp including gene ARL6)
Chr3 (98,917,287)
Chr3 (99,007,383)
Exon16
exon17
exon18
Exon1
Exon2
Exon3
| |
| --- |
DKFZp667G2110 (19 exons, 1-19 coding) NM_153605
EPHA6 (23 exons, 1-23 coding)
NM_001080448
2 transcriptome read map to exon 1.
41 reads map to exon 10 to exon 19
| Gene Symbol | SID559 | SID524 | SID557 | SID558 | SID816 | SID1276 |
| --- | --- | --- | --- | --- | --- | --- |
| EPHA6 | 2 | 0 | 0 | 0 | 0 | 2 |
| DKFZp667G2110 | 41 | 22 | 61 | 36 | 106 | 43 |
Typical DNA read and direction

## Slide 3
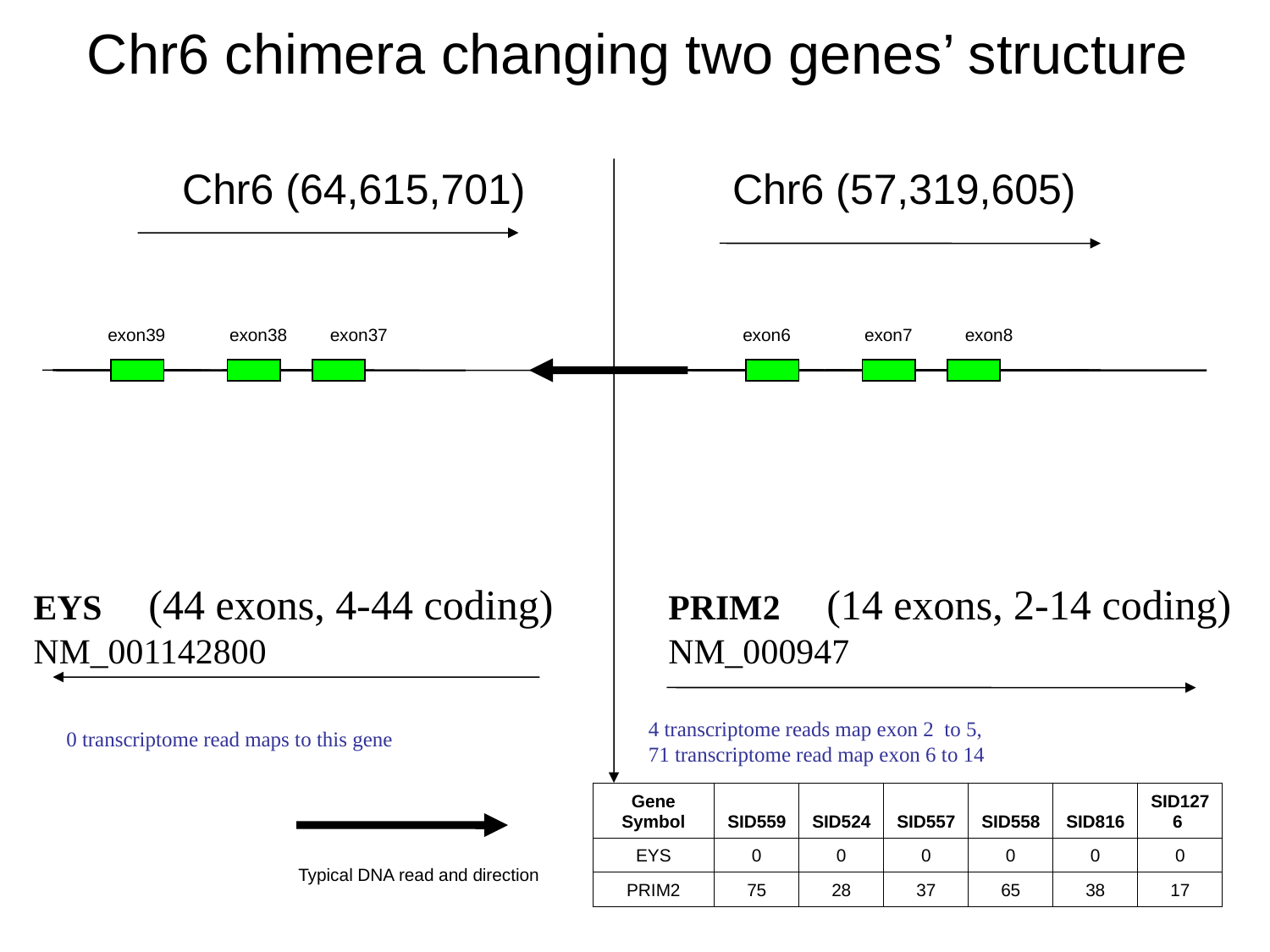

Chr6 chimera changing two genes’ structure
Chr6 (64,615,701)
Chr6 (57,319,605)
exon39
exon38
exon37
exon6
exon7
exon8
| |
| --- |
| |
| --- |
EYS (44 exons, 4-44 coding)
NM_001142800
PRIM2 (14 exons, 2-14 coding)
NM_000947
4 transcriptome reads map exon 2 to 5,
71 transcriptome read map exon 6 to 14
0 transcriptome read maps to this gene
| Gene Symbol | SID559 | SID524 | SID557 | SID558 | SID816 | SID1276 |
| --- | --- | --- | --- | --- | --- | --- |
| EYS | 0 | 0 | 0 | 0 | 0 | 0 |
| PRIM2 | 75 | 28 | 37 | 65 | 38 | 17 |
Typical DNA read and direction

## Slide 4
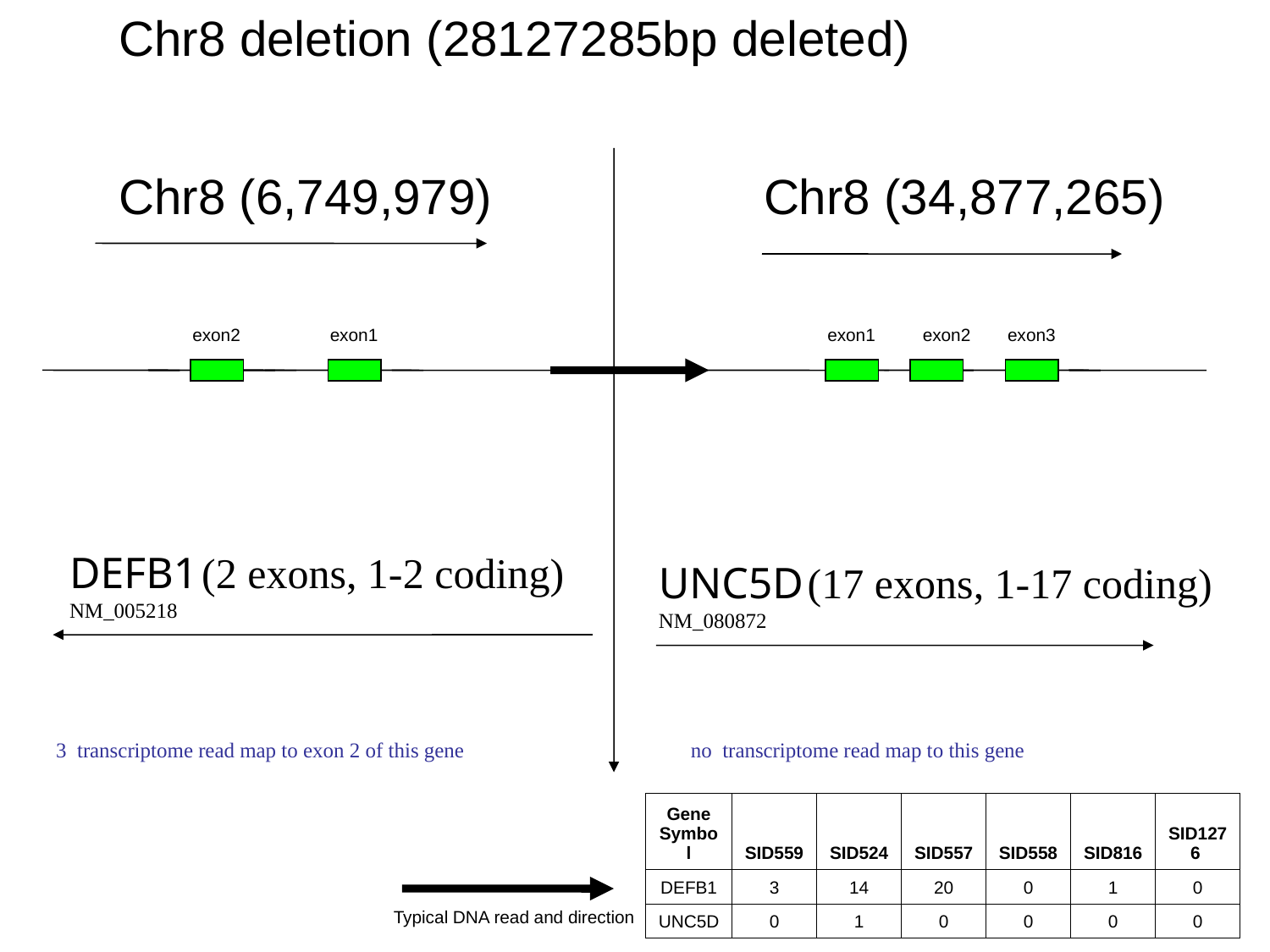

Chr8 deletion (28127285bp deleted)
Chr8 (6,749,979)
Chr8 (34,877,265)
exon2
exon1
exon1
exon2
exon3
| |
| --- |
DEFB1 (2 exons, 1-2 coding)
NM_005218
| |
| --- |
UNC5D (17 exons, 1-17 coding)
NM_080872
3 transcriptome read map to exon 2 of this gene
no transcriptome read map to this gene
| Gene Symbol | SID559 | SID524 | SID557 | SID558 | SID816 | SID1276 |
| --- | --- | --- | --- | --- | --- | --- |
| DEFB1 | 3 | 14 | 20 | 0 | 1 | 0 |
| UNC5D | 0 | 1 | 0 | 0 | 0 | 0 |
Typical DNA read and direction

## Slide 5
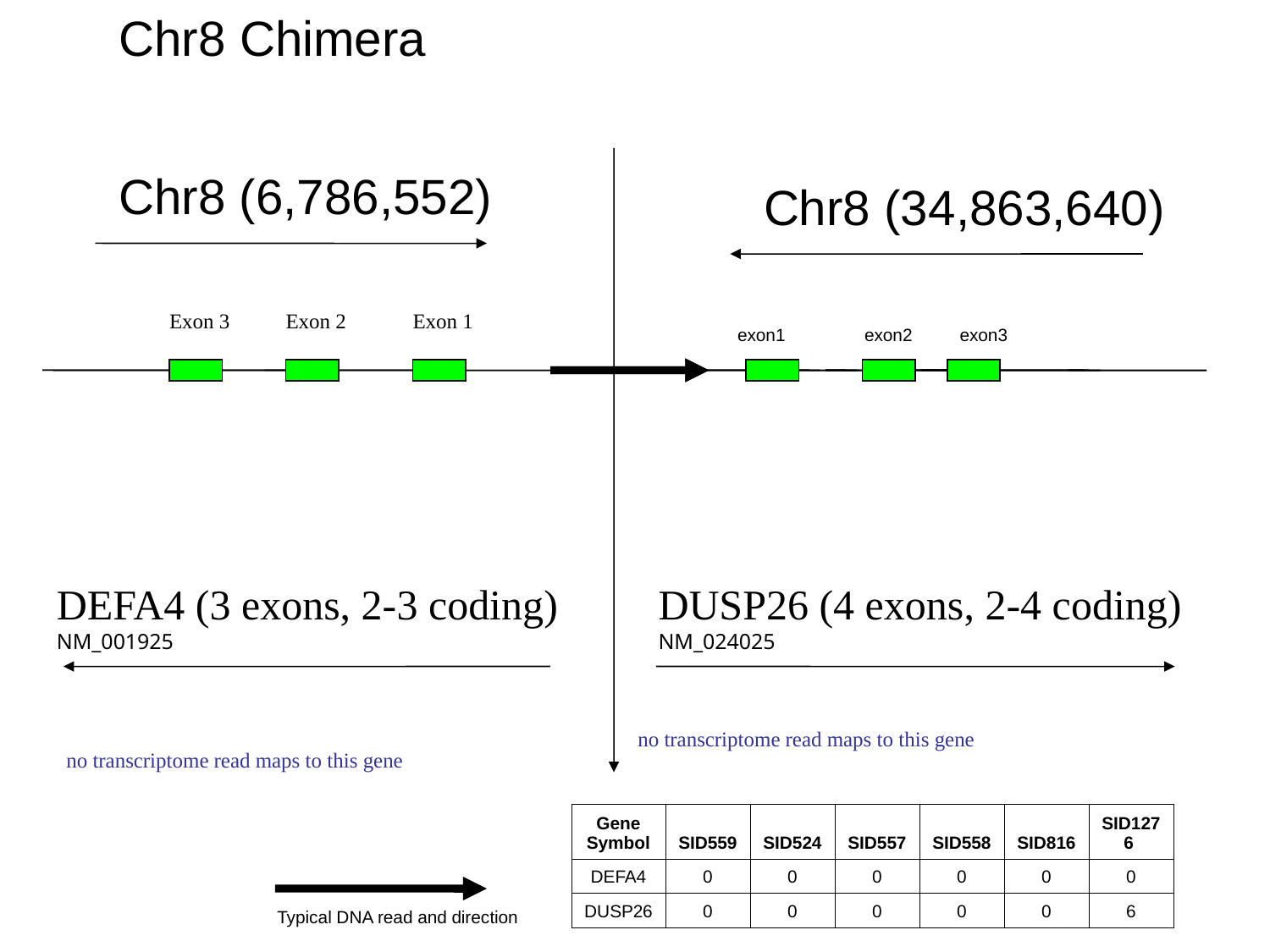

Chr8 Chimera
Chr8 (6,786,552)
Chr8 (34,863,640)
Exon 3
Exon 2
Exon 1
exon1
exon2
exon3
| |
| --- |
| |
| --- |
DEFA4 (3 exons, 2-3 coding)
NM_001925
DUSP26 (4 exons, 2-4 coding)
NM_024025
no transcriptome read maps to this gene
no transcriptome read maps to this gene
| Gene Symbol | SID559 | SID524 | SID557 | SID558 | SID816 | SID1276 |
| --- | --- | --- | --- | --- | --- | --- |
| DEFA4 | 0 | 0 | 0 | 0 | 0 | 0 |
| DUSP26 | 0 | 0 | 0 | 0 | 0 | 6 |
Typical DNA read and direction

## Slide 6
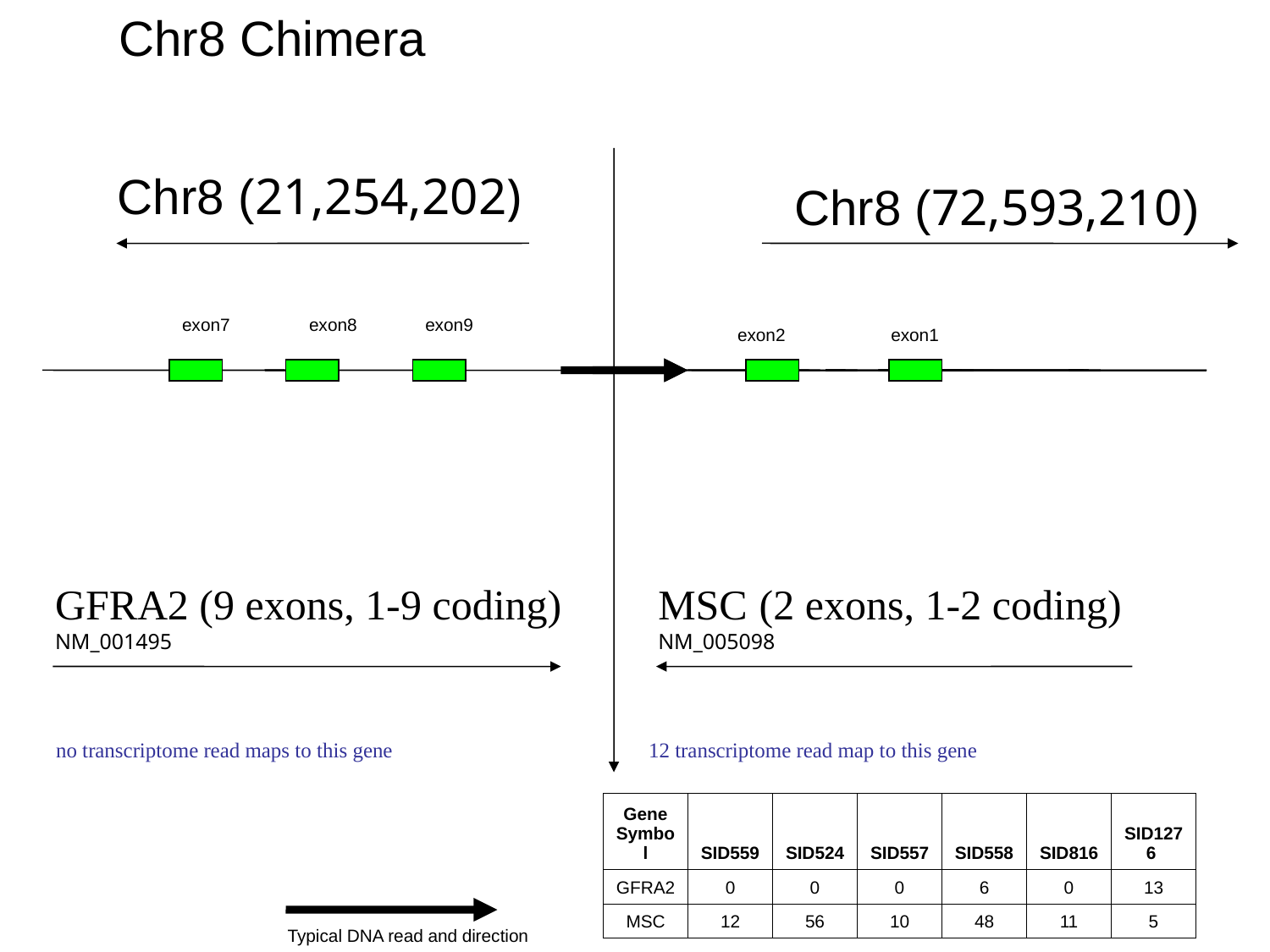

Chr8 Chimera
Chr8 (21,254,202)
Chr8 (72,593,210)
exon7
exon8
exon9
exon2
exon1
| |
| --- |
| |
| --- |
GFRA2 (9 exons, 1-9 coding)
NM_001495
MSC (2 exons, 1-2 coding)
NM_005098
no transcriptome read maps to this gene
12 transcriptome read map to this gene
| Gene Symbol | SID559 | SID524 | SID557 | SID558 | SID816 | SID1276 |
| --- | --- | --- | --- | --- | --- | --- |
| GFRA2 | 0 | 0 | 0 | 6 | 0 | 13 |
| MSC | 12 | 56 | 10 | 48 | 11 | 5 |
Typical DNA read and direction

## Slide 7
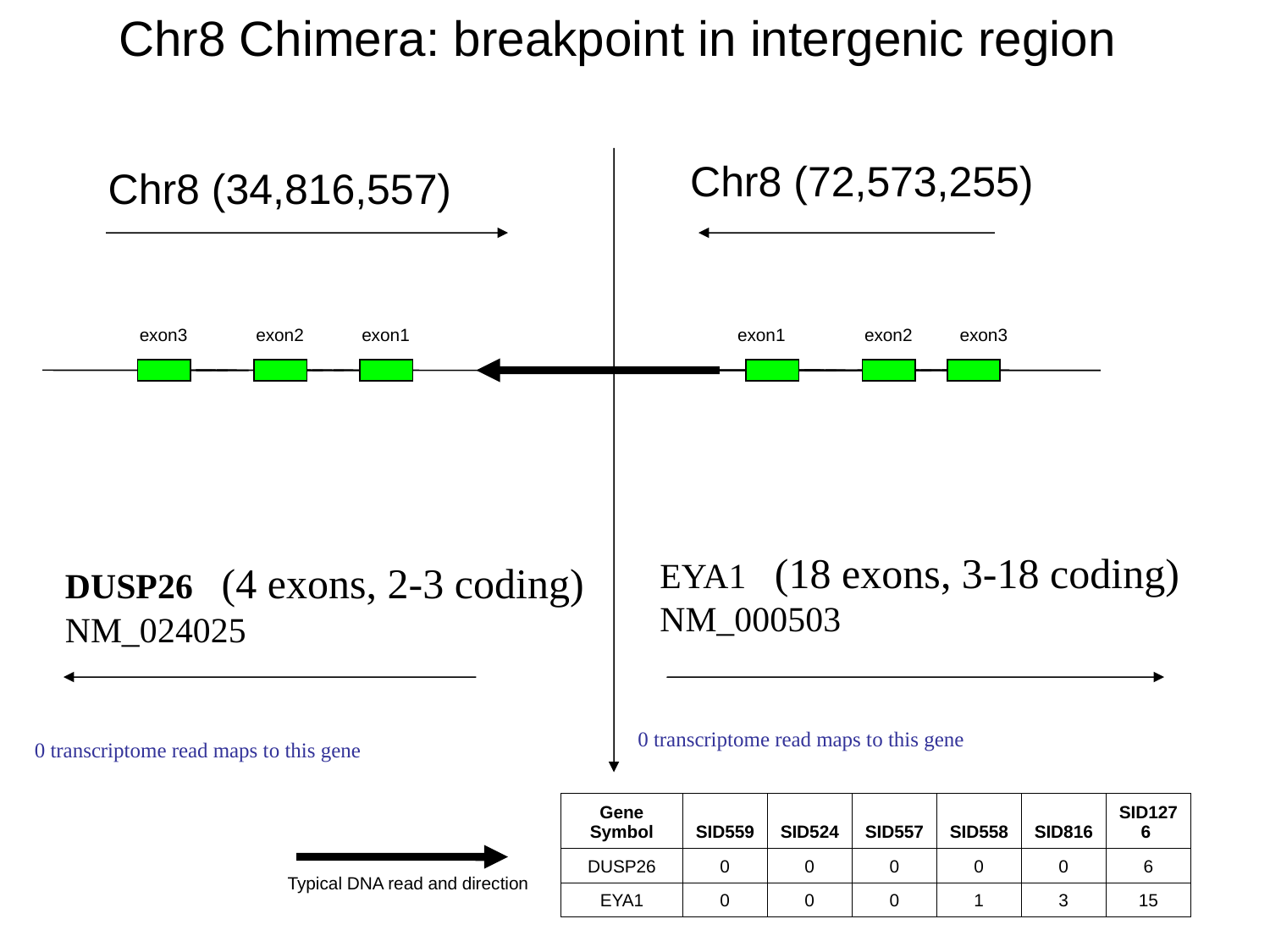

Chr8 Chimera: breakpoint in intergenic region
Chr8 (72,573,255)
Chr8 (34,816,557)
exon3
exon2
exon1
exon1
exon2
exon3
EYA1 (18 exons, 3-18 coding)
NM_000503
| |
| --- |
DUSP26 (4 exons, 2-3 coding)
NM_024025
0 transcriptome read maps to this gene
0 transcriptome read maps to this gene
| Gene Symbol | SID559 | SID524 | SID557 | SID558 | SID816 | SID1276 |
| --- | --- | --- | --- | --- | --- | --- |
| DUSP26 | 0 | 0 | 0 | 0 | 0 | 6 |
| EYA1 | 0 | 0 | 0 | 1 | 3 | 15 |
Typical DNA read and direction

## Slide 8
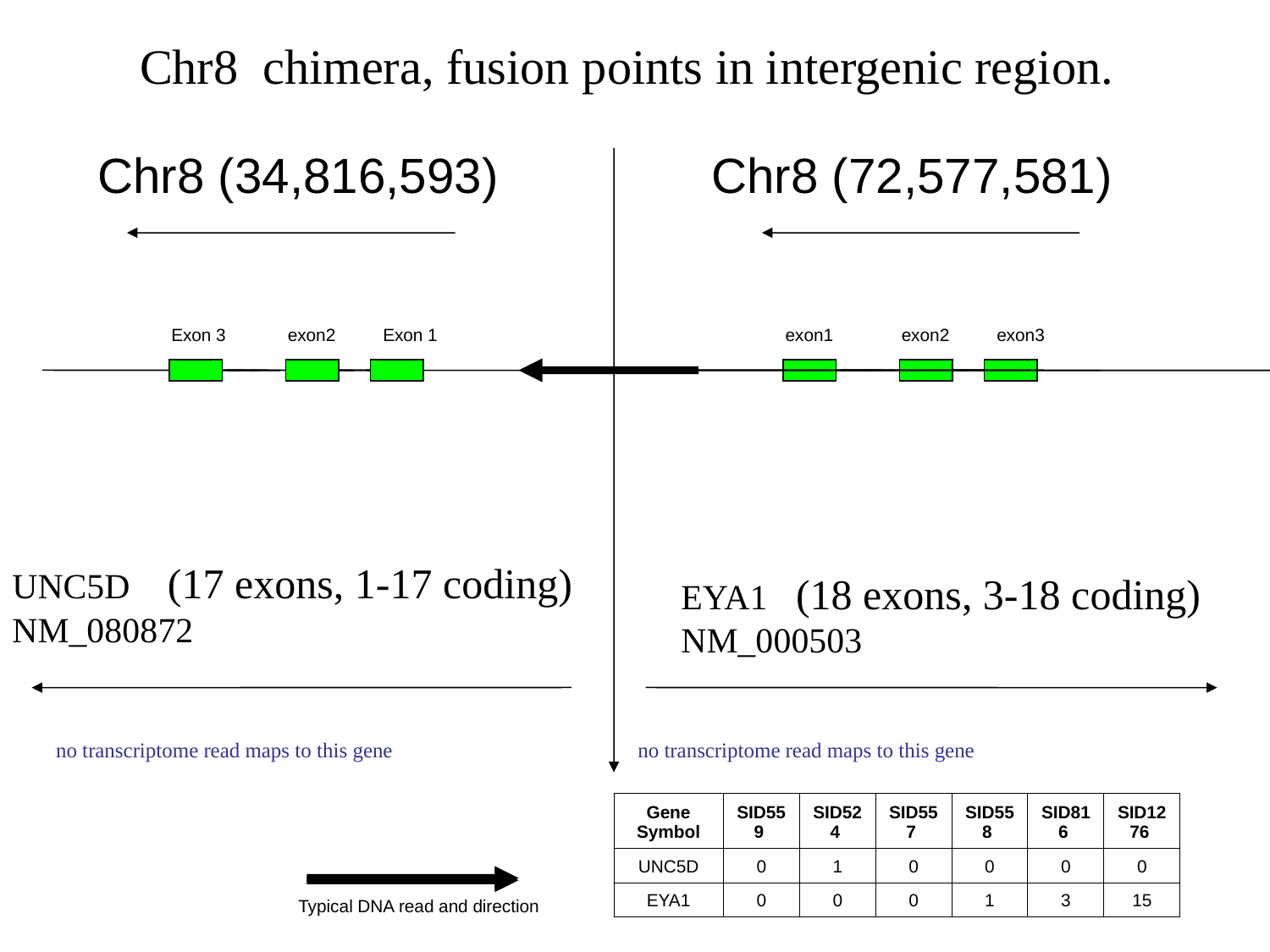

Chr8 chimera, fusion points in intergenic region.
Chr8 (34,816,593)
Chr8 (72,577,581)
Exon 3
exon2
Exon 1
exon1
exon2
exon3
| |
| --- |
UNC5D (17 exons, 1-17 coding)
NM_080872
EYA1 (18 exons, 3-18 coding)
NM_000503
no transcriptome read maps to this gene
no transcriptome read maps to this gene
| Gene Symbol | SID559 | SID524 | SID557 | SID558 | SID816 | SID1276 |
| --- | --- | --- | --- | --- | --- | --- |
| UNC5D | 0 | 1 | 0 | 0 | 0 | 0 |
| EYA1 | 0 | 0 | 0 | 1 | 3 | 15 |
Typical DNA read and direction

## Slide 9
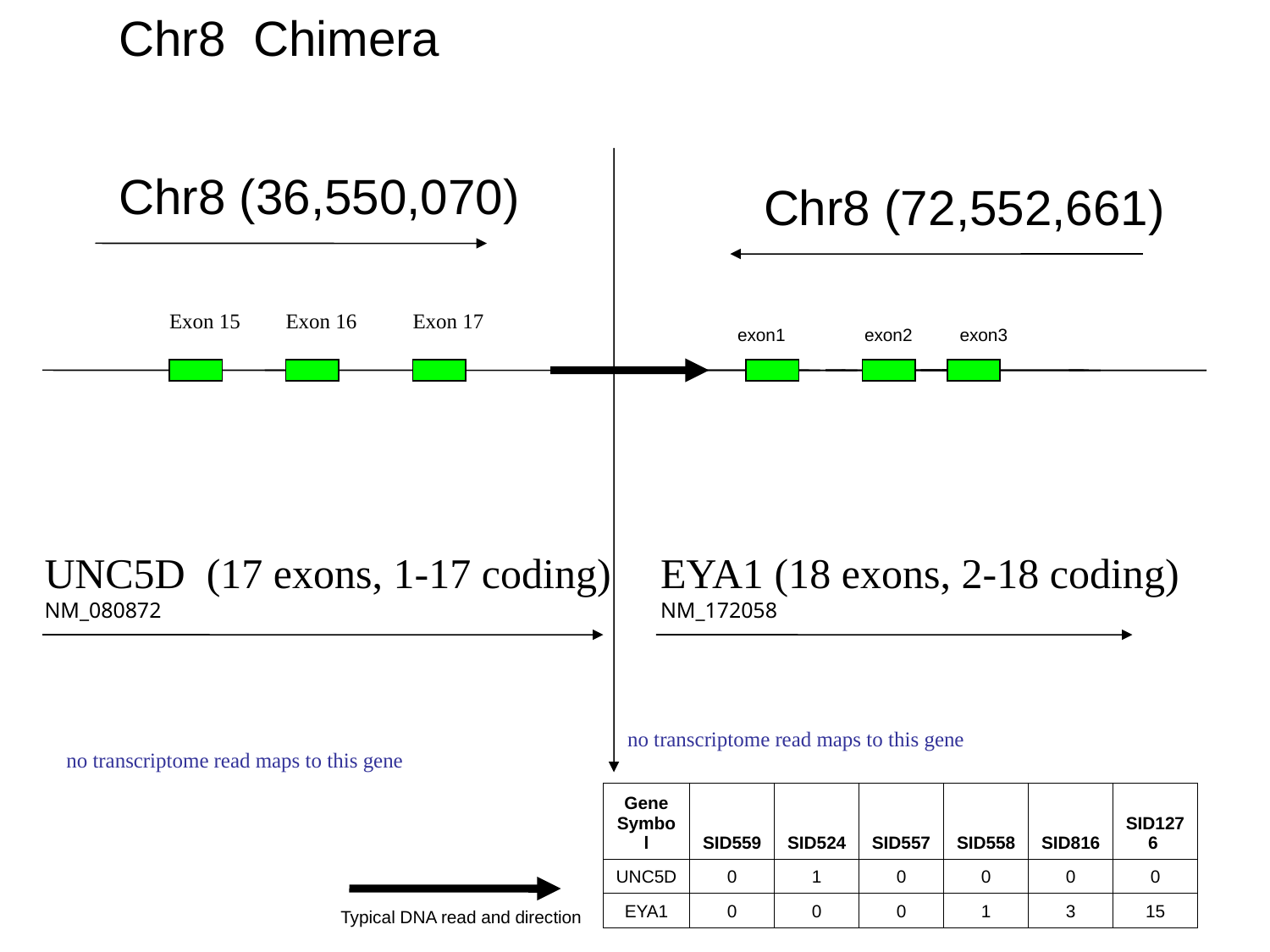

Chr8 Chimera
Chr8 (36,550,070)
Chr8 (72,552,661)
Exon 15
Exon 16
Exon 17
exon1
exon2
exon3
| |
| --- |
UNC5D (17 exons, 1-17 coding)
NM_080872
EYA1 (18 exons, 2-18 coding)
NM_172058
| |
| --- |
no transcriptome read maps to this gene
no transcriptome read maps to this gene
| Gene Symbol | SID559 | SID524 | SID557 | SID558 | SID816 | SID1276 |
| --- | --- | --- | --- | --- | --- | --- |
| UNC5D | 0 | 1 | 0 | 0 | 0 | 0 |
| EYA1 | 0 | 0 | 0 | 1 | 3 | 15 |
Typical DNA read and direction

## Slide 10
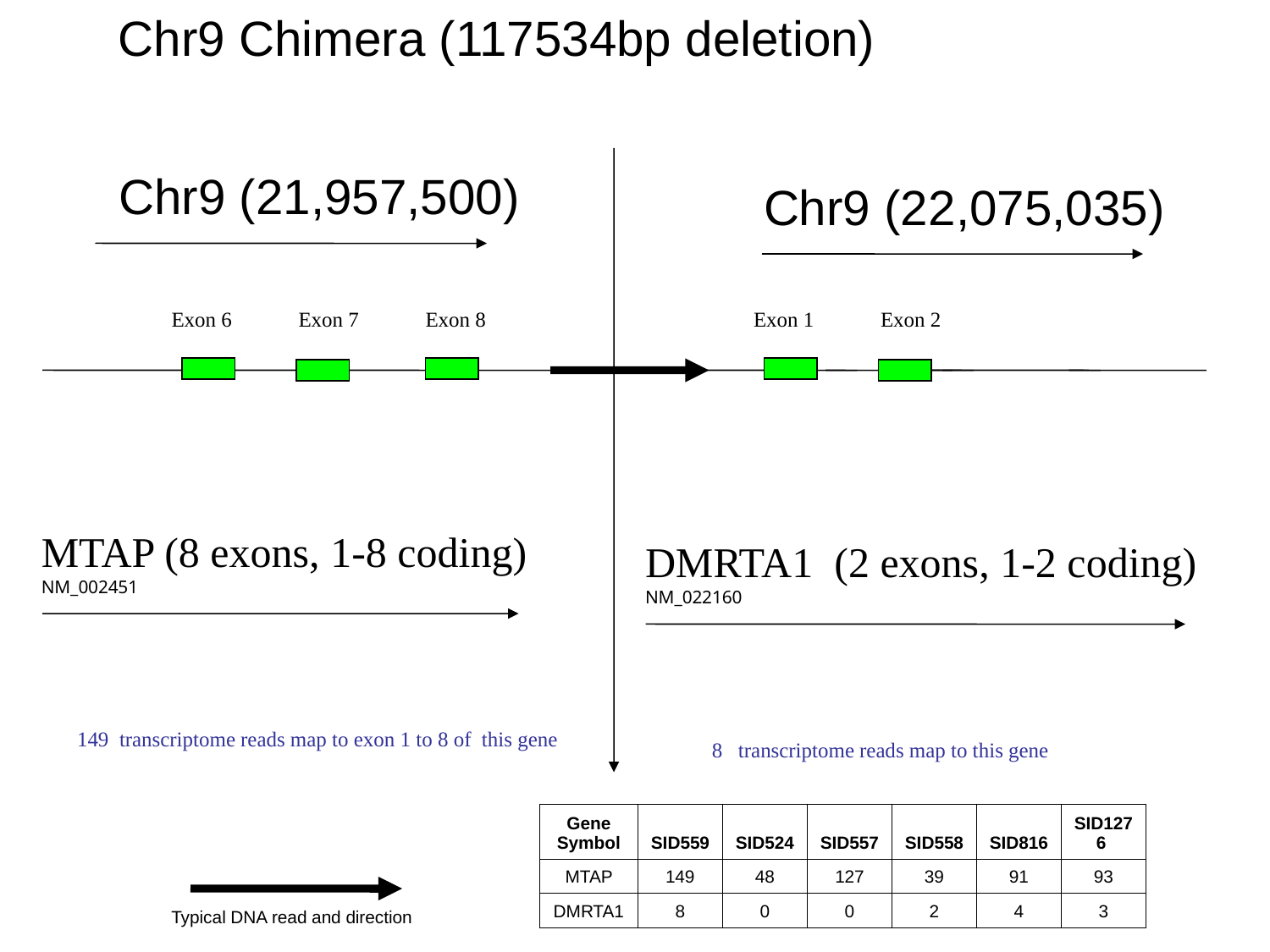

Chr9 Chimera (117534bp deletion)
Chr9 (21,957,500)
Chr9 (22,075,035)
Exon 6
Exon 7
Exon 8
Exon 1
Exon 2
MTAP (8 exons, 1-8 coding)
NM_002451
DMRTA1 (2 exons, 1-2 coding)
NM_022160
| |
| --- |
| |
| --- |
| |
| --- |
149 transcriptome reads map to exon 1 to 8 of this gene
8 transcriptome reads map to this gene
| Gene Symbol | SID559 | SID524 | SID557 | SID558 | SID816 | SID1276 |
| --- | --- | --- | --- | --- | --- | --- |
| MTAP | 149 | 48 | 127 | 39 | 91 | 93 |
| DMRTA1 | 8 | 0 | 0 | 2 | 4 | 3 |
Typical DNA read and direction

## Slide 11
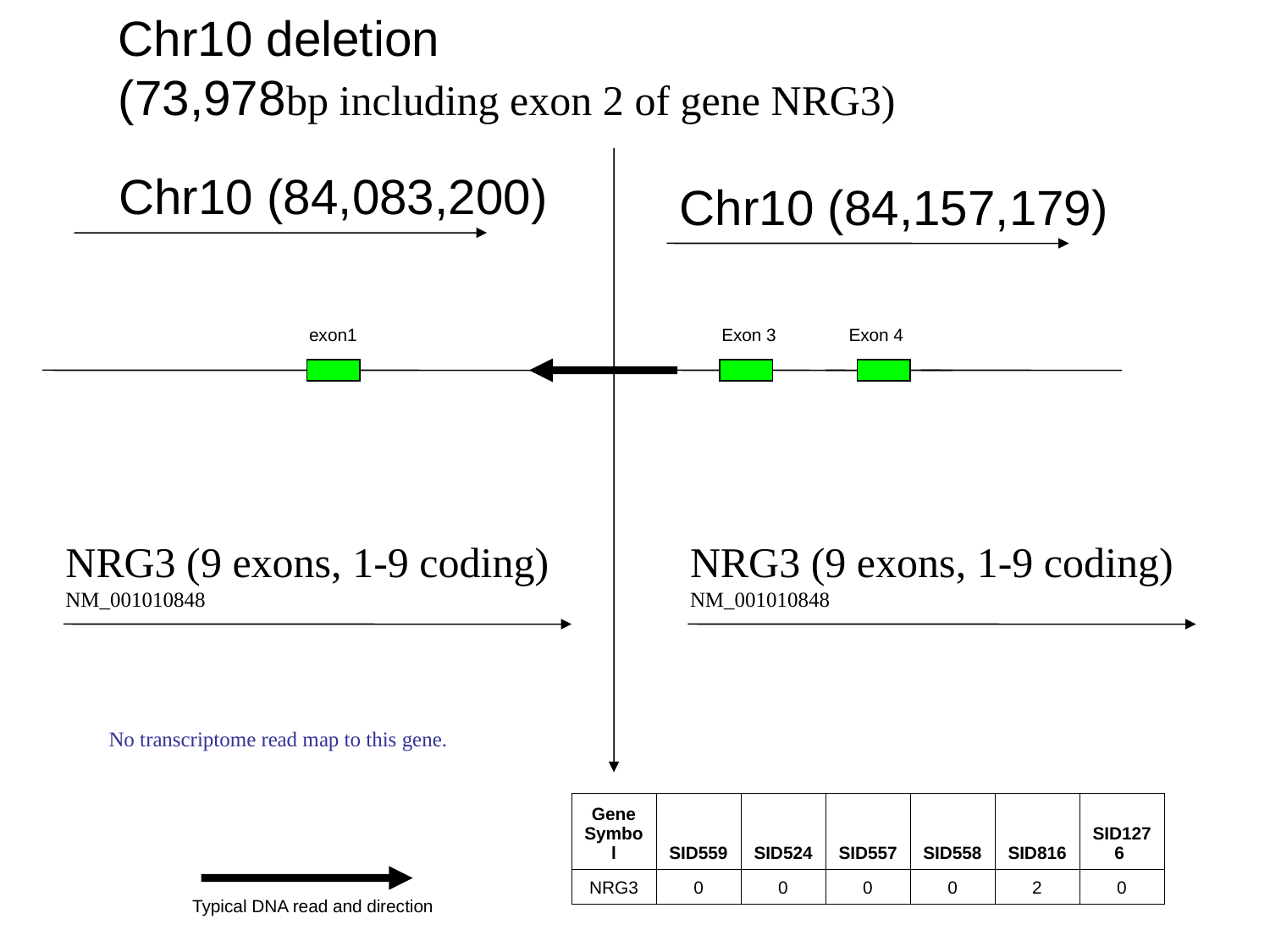

Chr10 deletion
(73,978bp including exon 2 of gene NRG3)
Chr10 (84,083,200)
Chr10 (84,157,179)
exon1
Exon 3
Exon 4
| |
| --- |
NRG3 (9 exons, 1-9 coding)
NM_001010848
NRG3 (9 exons, 1-9 coding)
NM_001010848
No transcriptome read map to this gene.
| Gene Symbol | SID559 | SID524 | SID557 | SID558 | SID816 | SID1276 |
| --- | --- | --- | --- | --- | --- | --- |
| NRG3 | 0 | 0 | 0 | 0 | 2 | 0 |
Typical DNA read and direction

## Slide 12
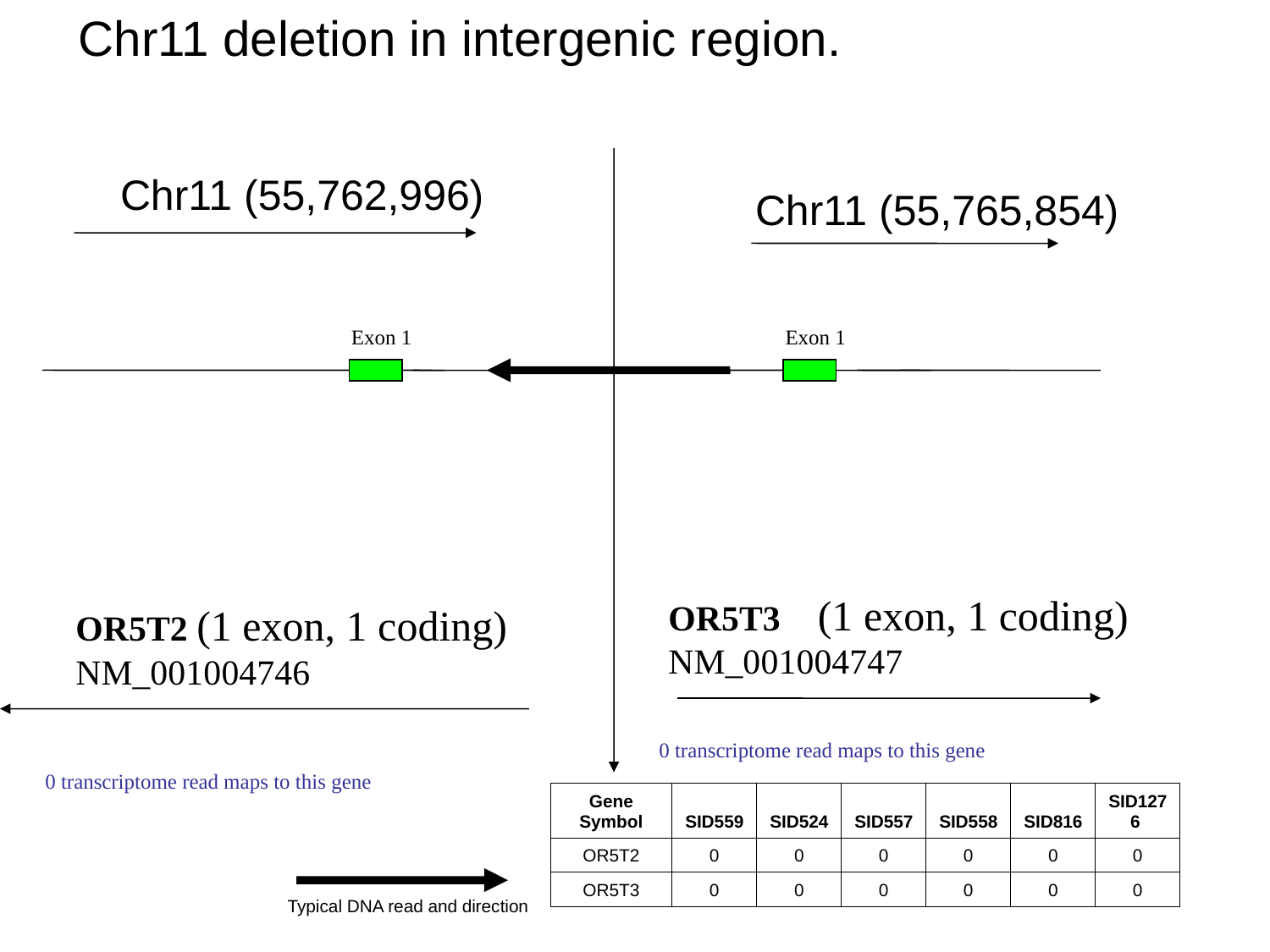

Chr11 deletion in intergenic region.
Chr11 (55,762,996)
Chr11 (55,765,854)
Exon 1
Exon 1
| |
| --- |
OR5T3 (1 exon, 1 coding)
NM_001004747
OR5T2 (1 exon, 1 coding)
NM_001004746
0 transcriptome read maps to this gene
0 transcriptome read maps to this gene
| Gene Symbol | SID559 | SID524 | SID557 | SID558 | SID816 | SID1276 |
| --- | --- | --- | --- | --- | --- | --- |
| OR5T2 | 0 | 0 | 0 | 0 | 0 | 0 |
| OR5T3 | 0 | 0 | 0 | 0 | 0 | 0 |
Typical DNA read and direction

## Slide 13
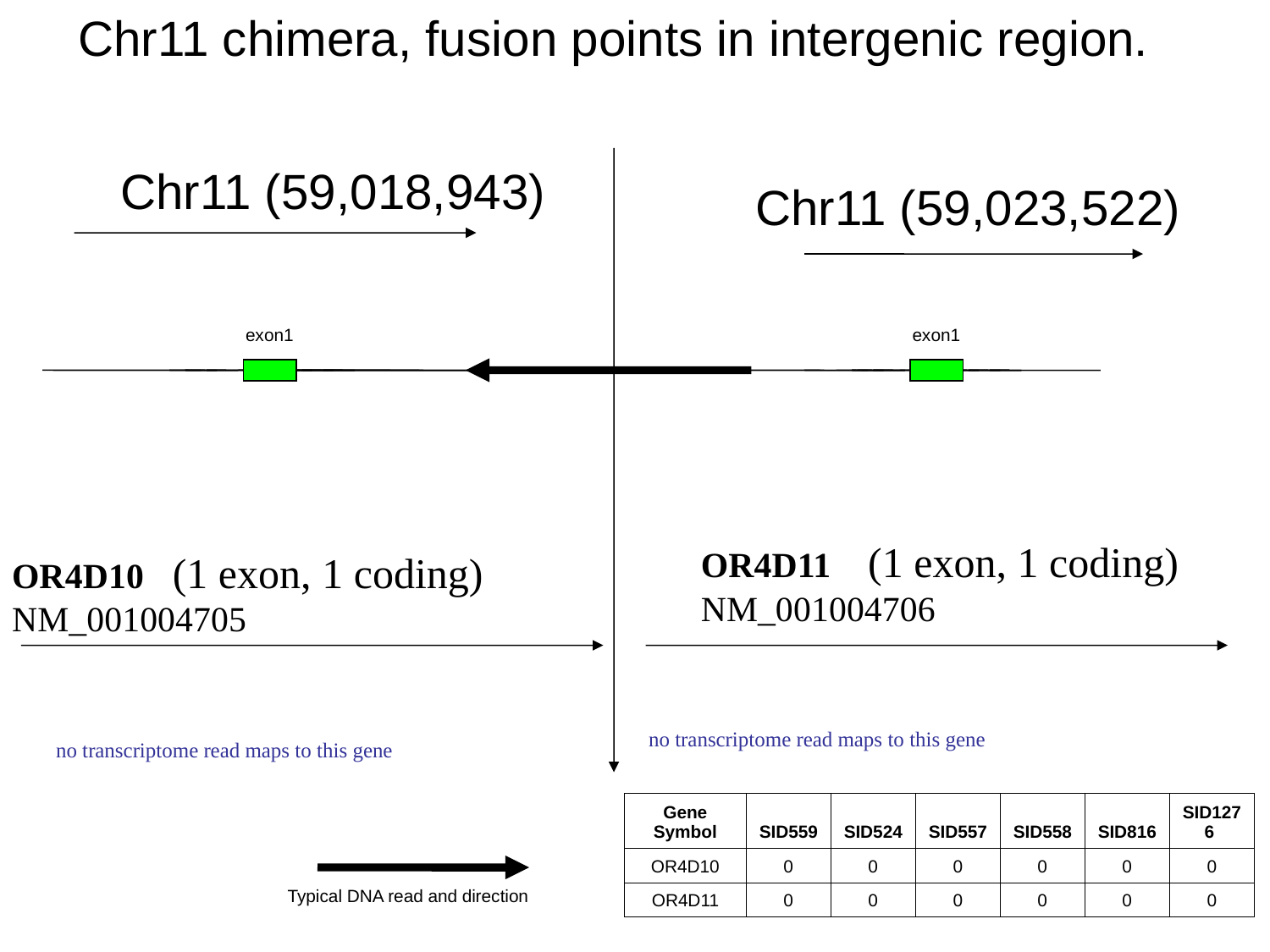

Chr11 chimera, fusion points in intergenic region.
Chr11 (59,018,943)
Chr11 (59,023,522)
exon1
exon1
OR4D11 (1 exon, 1 coding)
NM_001004706
OR4D10 (1 exon, 1 coding)
NM_001004705
| |
| --- |
no transcriptome read maps to this gene
no transcriptome read maps to this gene
| Gene Symbol | SID559 | SID524 | SID557 | SID558 | SID816 | SID1276 |
| --- | --- | --- | --- | --- | --- | --- |
| OR4D10 | 0 | 0 | 0 | 0 | 0 | 0 |
| OR4D11 | 0 | 0 | 0 | 0 | 0 | 0 |
Typical DNA read and direction

## Slide 14
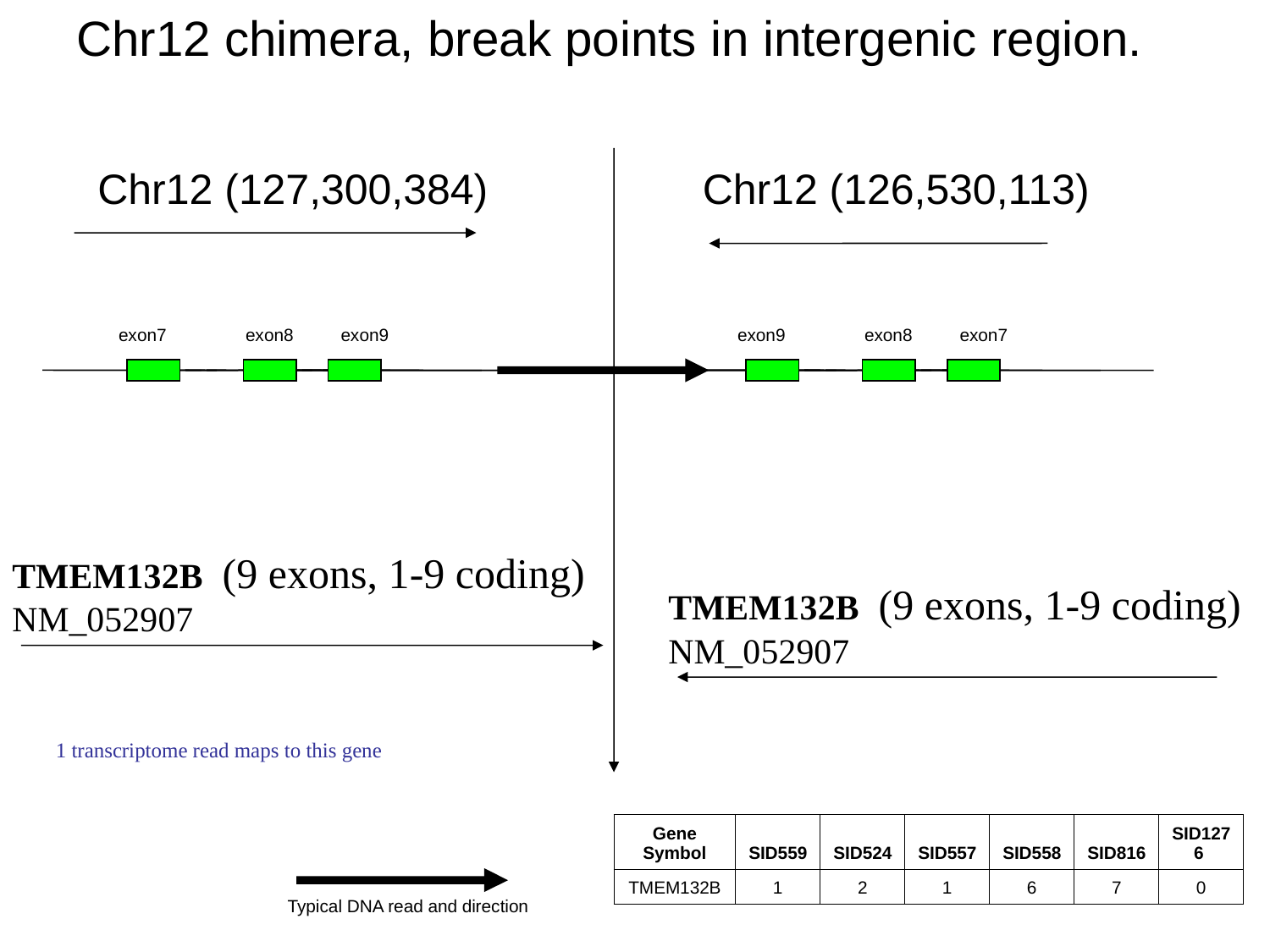

Chr12 chimera, break points in intergenic region.
Chr12 (127,300,384)
Chr12 (126,530,113)
exon7
exon8
exon9
exon9
exon8
exon7
TMEM132B (9 exons, 1-9 coding)
NM_052907
| |
| --- |
TMEM132B (9 exons, 1-9 coding)
NM_052907
1 transcriptome read maps to this gene
| Gene Symbol | SID559 | SID524 | SID557 | SID558 | SID816 | SID1276 |
| --- | --- | --- | --- | --- | --- | --- |
| TMEM132B | 1 | 2 | 1 | 6 | 7 | 0 |
Typical DNA read and direction

## Slide 15
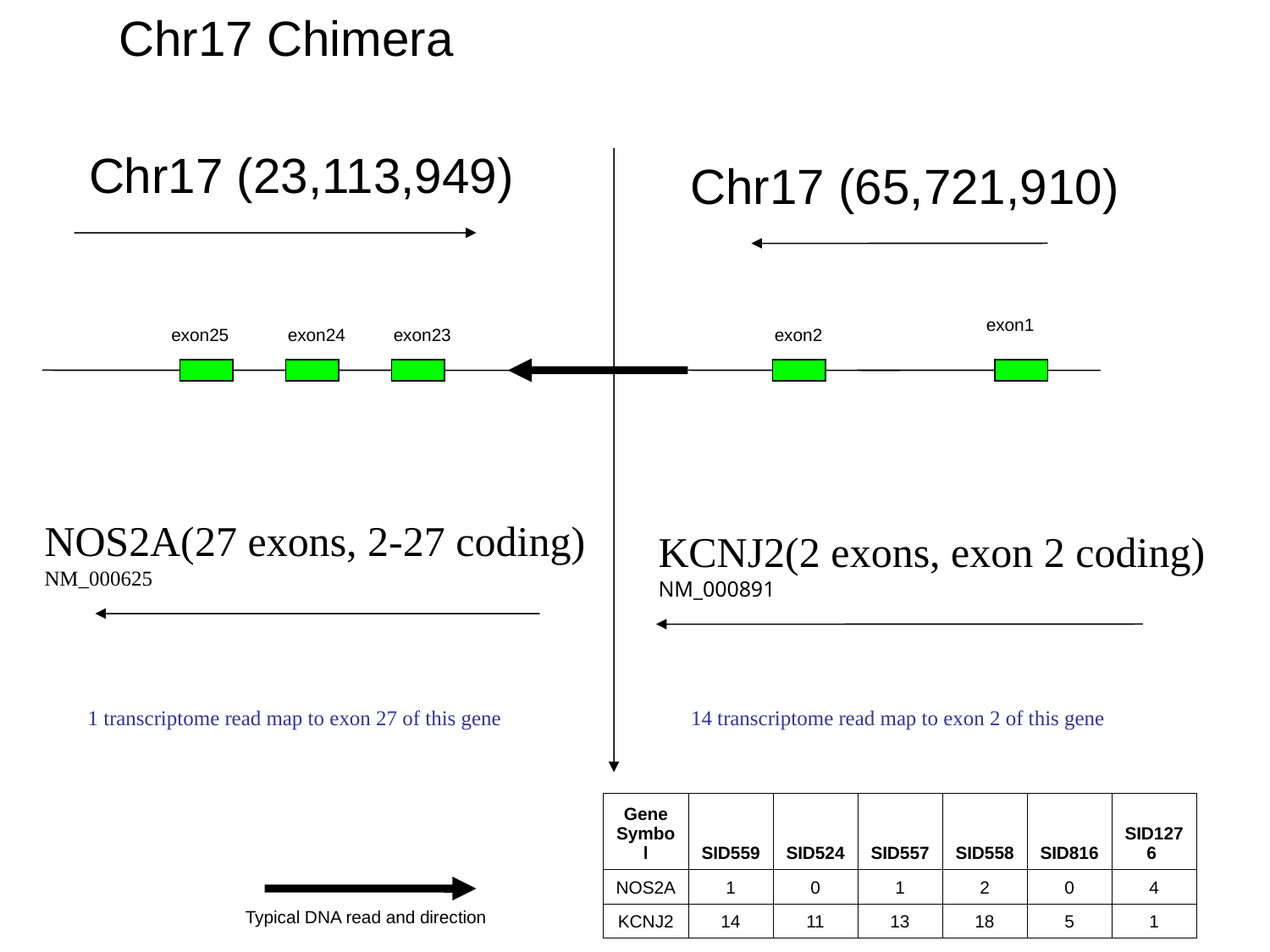

Chr17 Chimera
Chr17 (23,113,949)
Chr17 (65,721,910)
exon1
exon25
exon24
exon23
exon2
NOS2A(27 exons, 2-27 coding)
NM_000625
KCNJ2(2 exons, exon 2 coding)
NM_000891
| |
| --- |
1 transcriptome read map to exon 27 of this gene
14 transcriptome read map to exon 2 of this gene
| Gene Symbol | SID559 | SID524 | SID557 | SID558 | SID816 | SID1276 |
| --- | --- | --- | --- | --- | --- | --- |
| NOS2A | 1 | 0 | 1 | 2 | 0 | 4 |
| KCNJ2 | 14 | 11 | 13 | 18 | 5 | 1 |
Typical DNA read and direction

## Slide 16
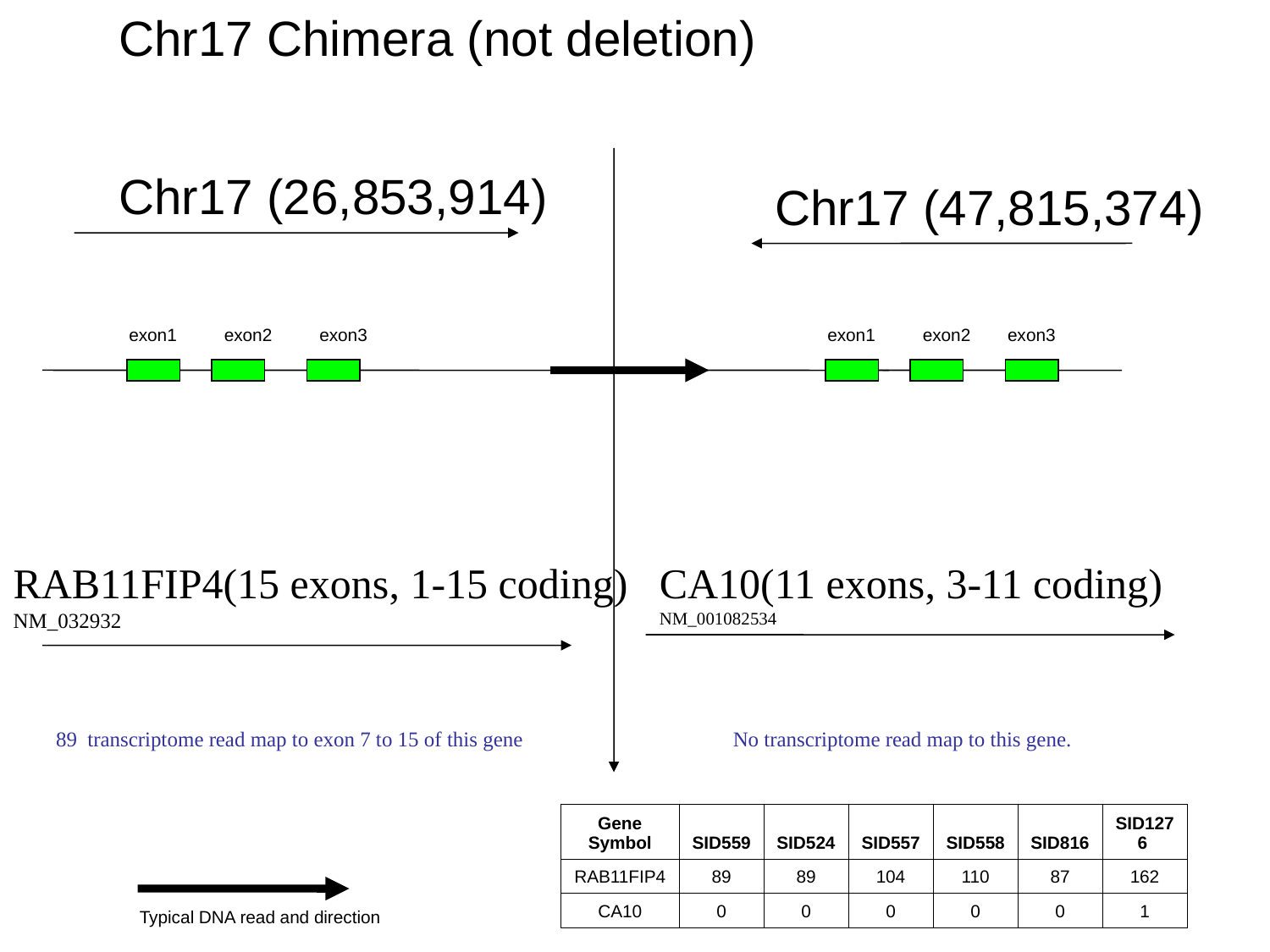

Chr17 Chimera (not deletion)
Chr17 (26,853,914)
Chr17 (47,815,374)
exon1
exon2
exon3
exon1
exon2
exon3
| |
| --- |
| |
| --- |
RAB11FIP4(15 exons, 1-15 coding)
NM_032932
CA10(11 exons, 3-11 coding)
NM_001082534
89 transcriptome read map to exon 7 to 15 of this gene
No transcriptome read map to this gene.
| Gene Symbol | SID559 | SID524 | SID557 | SID558 | SID816 | SID1276 |
| --- | --- | --- | --- | --- | --- | --- |
| RAB11FIP4 | 89 | 89 | 104 | 110 | 87 | 162 |
| CA10 | 0 | 0 | 0 | 0 | 0 | 1 |
Typical DNA read and direction

## Slide 17
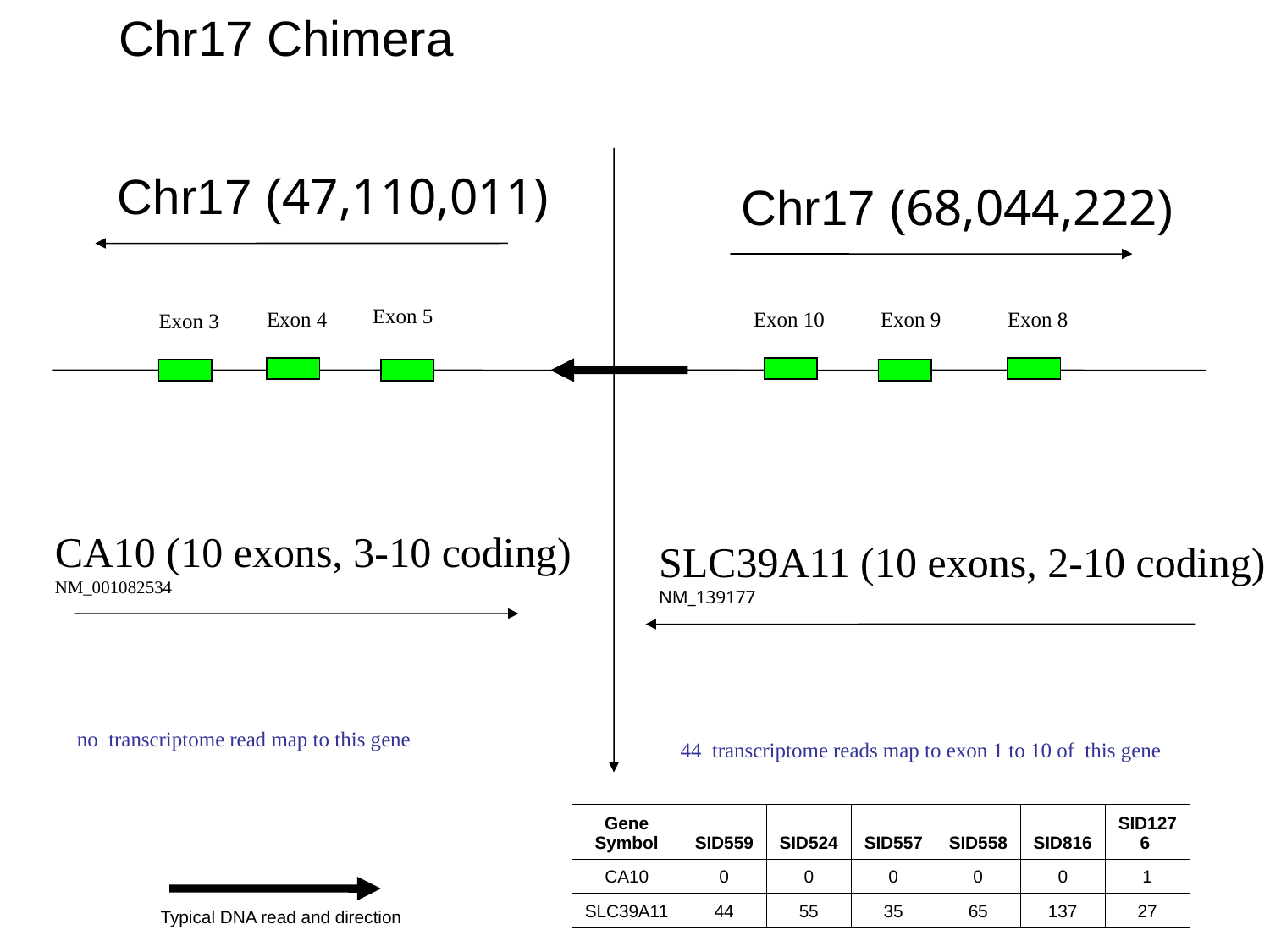

Chr17 Chimera
Chr17 (47,110,011)
Chr17 (68,044,222)
Exon 5
Exon 4
Exon 10
Exon 9
Exon 8
Exon 3
CA10 (10 exons, 3-10 coding)
NM_001082534
SLC39A11 (10 exons, 2-10 coding)
NM_139177
| |
| --- |
| |
| --- |
no transcriptome read map to this gene
44 transcriptome reads map to exon 1 to 10 of this gene
| Gene Symbol | SID559 | SID524 | SID557 | SID558 | SID816 | SID1276 |
| --- | --- | --- | --- | --- | --- | --- |
| CA10 | 0 | 0 | 0 | 0 | 0 | 1 |
| SLC39A11 | 44 | 55 | 35 | 65 | 137 | 27 |
Typical DNA read and direction

## Slide 18
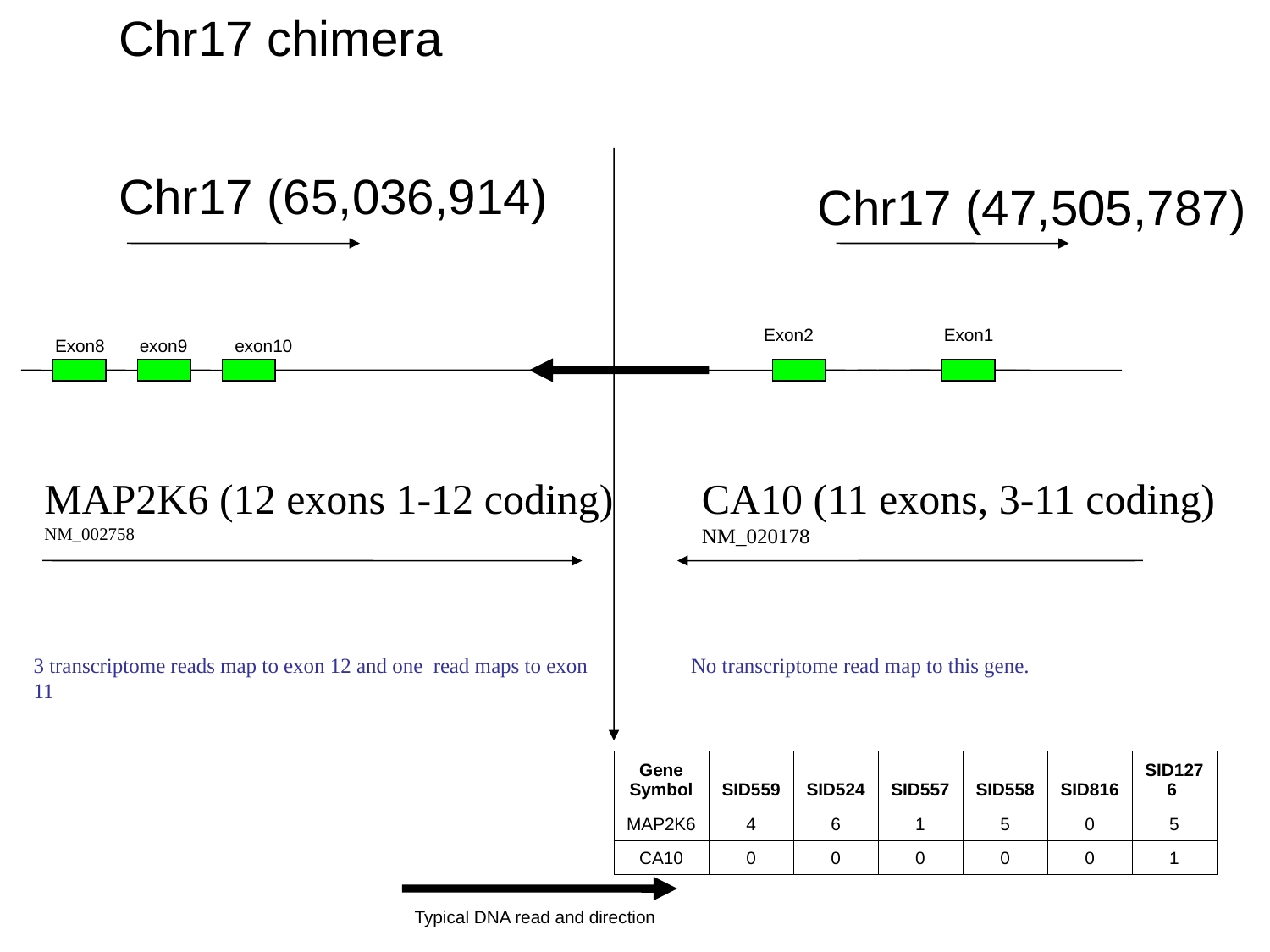

Chr17 chimera
Chr17 (65,036,914)
Chr17 (47,505,787)
Exon2
Exon1
Exon8
exon9
exon10
| |
| --- |
MAP2K6 (12 exons 1-12 coding)
NM_002758
CA10 (11 exons, 3-11 coding)
NM_020178
3 transcriptome reads map to exon 12 and one read maps to exon 11
No transcriptome read map to this gene.
| Gene Symbol | SID559 | SID524 | SID557 | SID558 | SID816 | SID1276 |
| --- | --- | --- | --- | --- | --- | --- |
| MAP2K6 | 4 | 6 | 1 | 5 | 0 | 5 |
| CA10 | 0 | 0 | 0 | 0 | 0 | 1 |
Typical DNA read and direction

## Slide 19
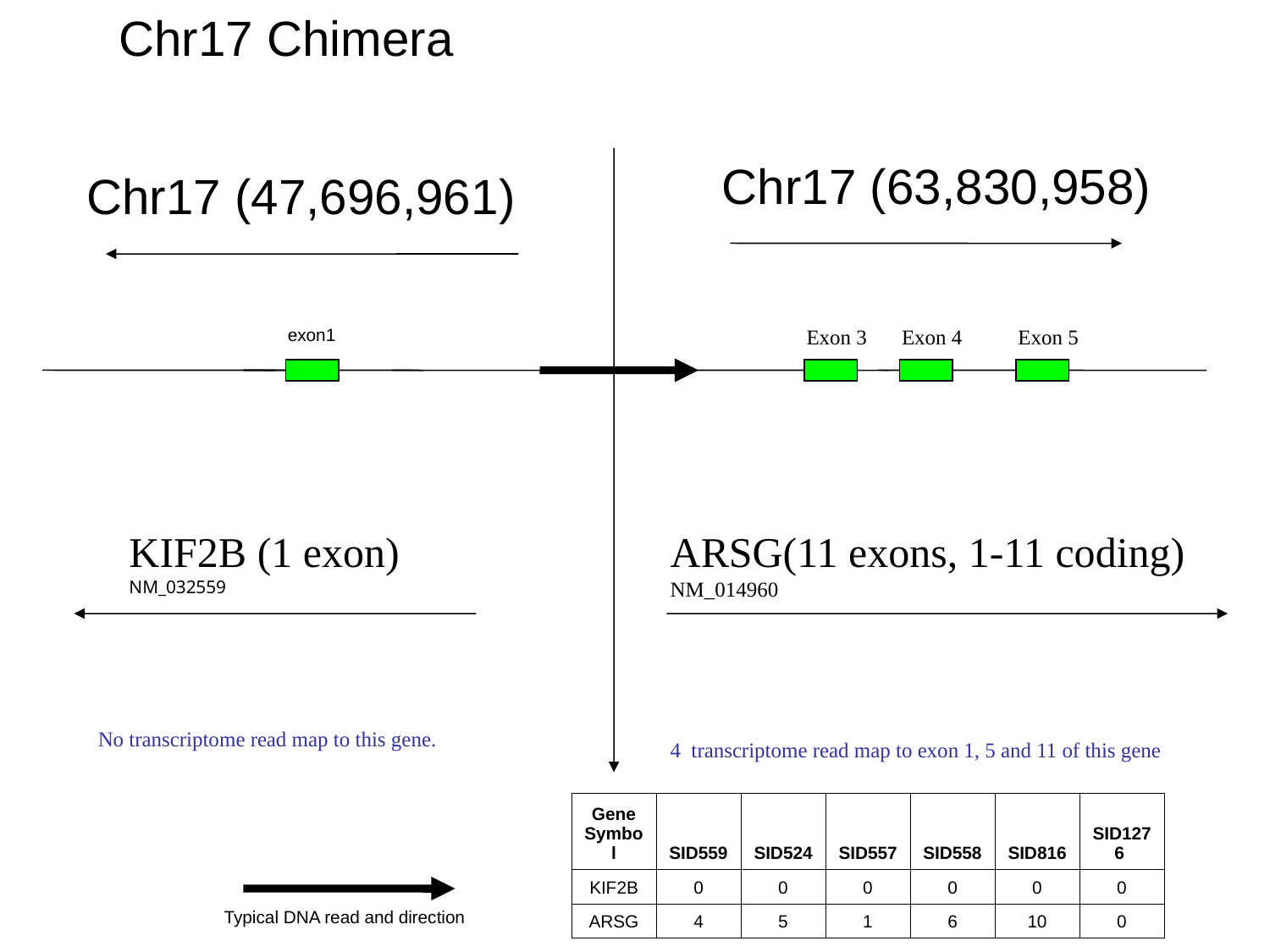

Chr17 Chimera
Chr17 (63,830,958)
Chr17 (47,696,961)
exon1
Exon 3
Exon 4
Exon 5
KIF2B (1 exon)
NM_032559
ARSG(11 exons, 1-11 coding)
NM_014960
| |
| --- |
| |
| --- |
No transcriptome read map to this gene.
4 transcriptome read map to exon 1, 5 and 11 of this gene
| Gene Symbol | SID559 | SID524 | SID557 | SID558 | SID816 | SID1276 |
| --- | --- | --- | --- | --- | --- | --- |
| KIF2B | 0 | 0 | 0 | 0 | 0 | 0 |
| ARSG | 4 | 5 | 1 | 6 | 10 | 0 |
Typical DNA read and direction

## Slide 20
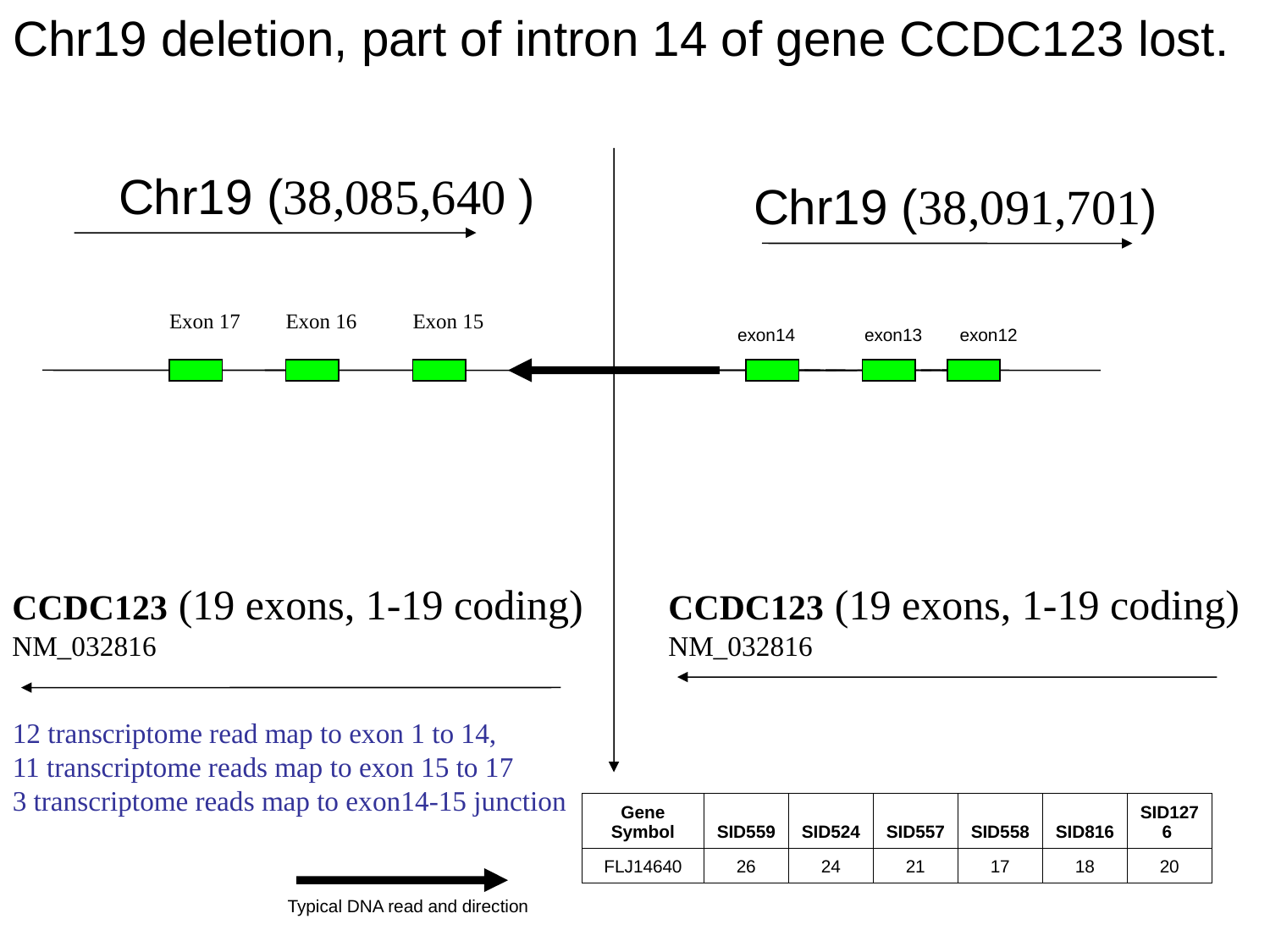

Chr19 deletion, part of intron 14 of gene CCDC123 lost.
Chr19 (38,085,640 )
Chr19 (38,091,701)
Exon 17
Exon 16
Exon 15
exon14
exon13
exon12
| |
| --- |
CCDC123 (19 exons, 1-19 coding)
NM_032816
CCDC123 (19 exons, 1-19 coding)
NM_032816
12 transcriptome read map to exon 1 to 14,
11 transcriptome reads map to exon 15 to 17
3 transcriptome reads map to exon14-15 junction
| Gene Symbol | SID559 | SID524 | SID557 | SID558 | SID816 | SID1276 |
| --- | --- | --- | --- | --- | --- | --- |
| FLJ14640 | 26 | 24 | 21 | 17 | 18 | 20 |
Typical DNA read and direction

## Slide 21
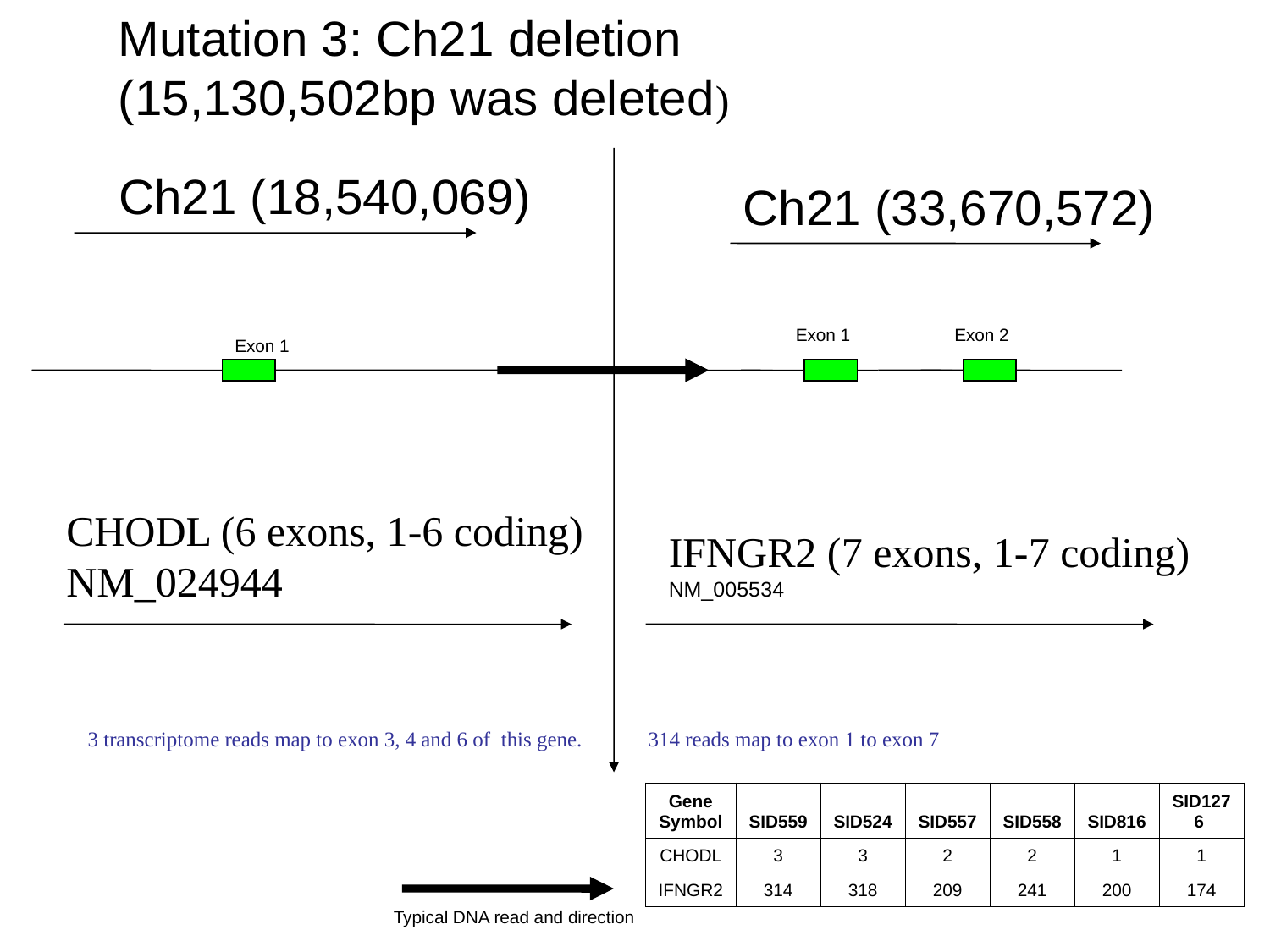

Mutation 3: Ch21 deletion
(15,130,502bp was deleted)
Ch21 (18,540,069)
Ch21 (33,670,572)
Exon 1
Exon 2
Exon 1
CHODL (6 exons, 1-6 coding)
NM_024944
IFNGR2 (7 exons, 1-7 coding) NM_005534
3 transcriptome reads map to exon 3, 4 and 6 of this gene.
314 reads map to exon 1 to exon 7
| Gene Symbol | SID559 | SID524 | SID557 | SID558 | SID816 | SID1276 |
| --- | --- | --- | --- | --- | --- | --- |
| CHODL | 3 | 3 | 2 | 2 | 1 | 1 |
| IFNGR2 | 314 | 318 | 209 | 241 | 200 | 174 |
Typical DNA read and direction

## Slide 22
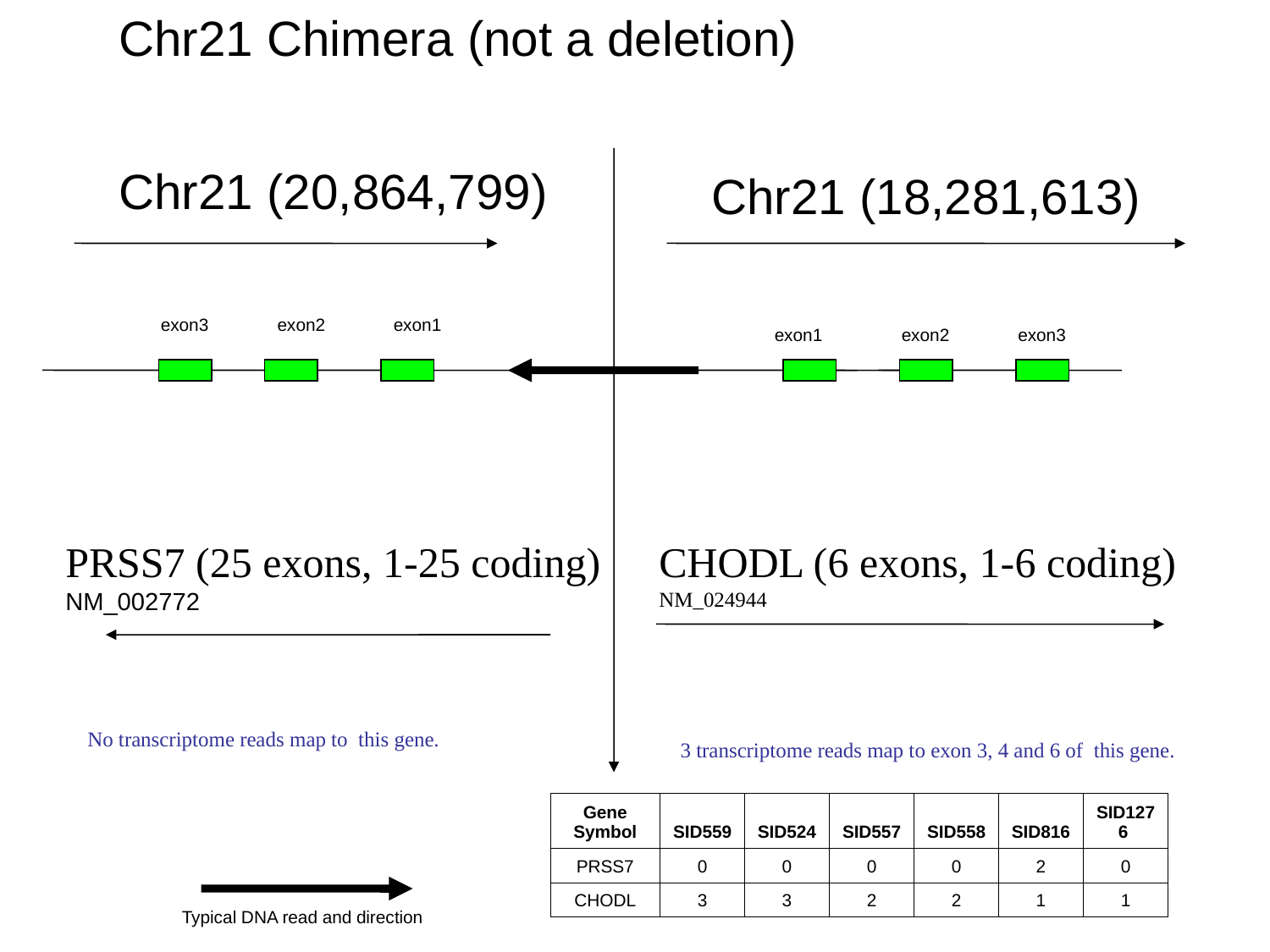

Chr21 Chimera (not a deletion)
Chr21 (20,864,799)
Chr21 (18,281,613)
exon3
exon2
exon1
exon1
exon2
exon3
PRSS7 (25 exons, 1-25 coding)
NM_002772
CHODL (6 exons, 1-6 coding)
NM_024944
| |
| --- |
No transcriptome reads map to this gene.
3 transcriptome reads map to exon 3, 4 and 6 of this gene.
| Gene Symbol | SID559 | SID524 | SID557 | SID558 | SID816 | SID1276 |
| --- | --- | --- | --- | --- | --- | --- |
| PRSS7 | 0 | 0 | 0 | 0 | 2 | 0 |
| CHODL | 3 | 3 | 2 | 2 | 1 | 1 |
Typical DNA read and direction

## Slide 23
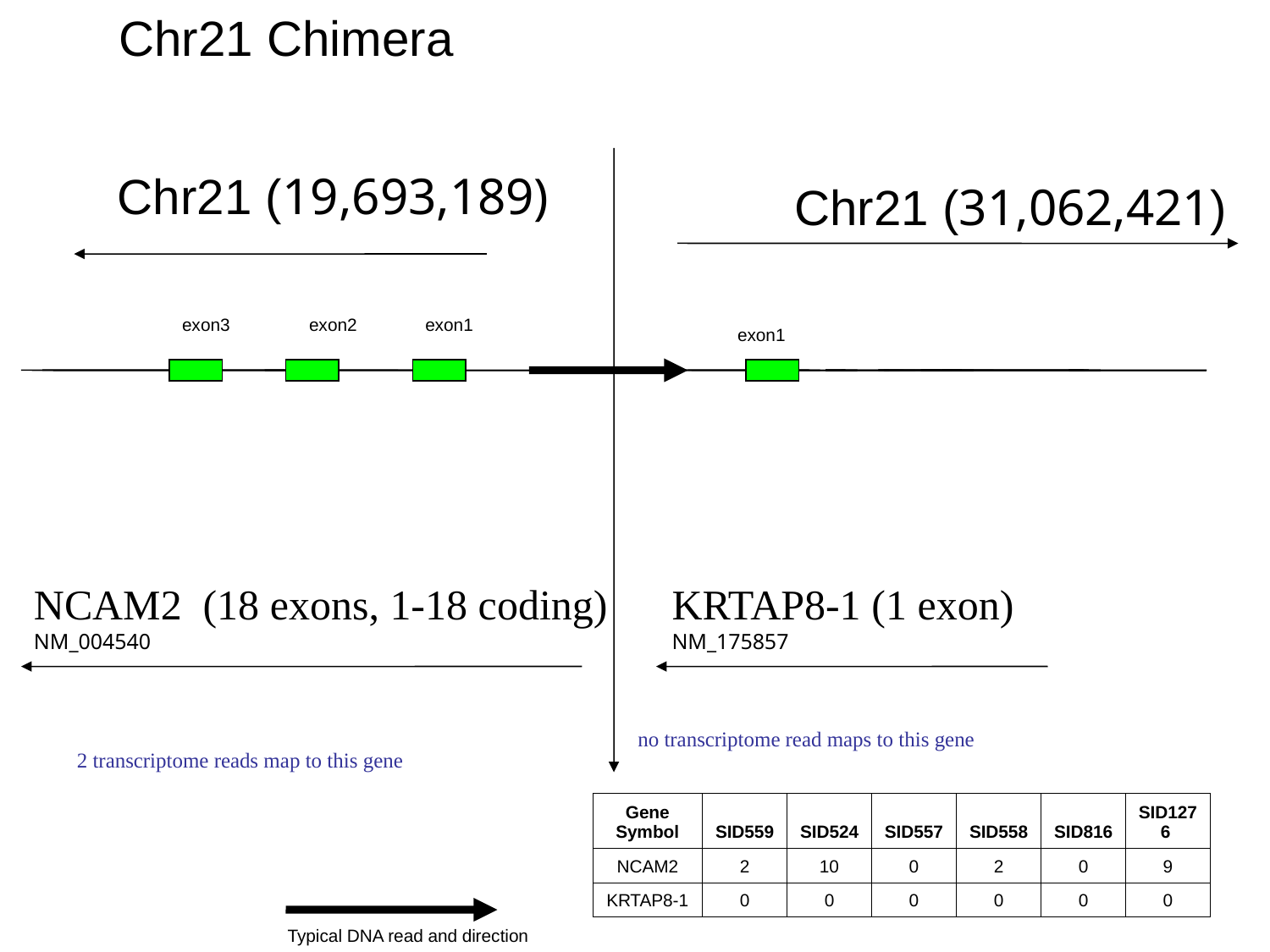

Chr21 Chimera
Chr21 (19,693,189)
Chr21 (31,062,421)
exon3
exon2
exon1
exon1
| |
| --- |
| |
| --- |
NCAM2 (18 exons, 1-18 coding)
NM_004540
KRTAP8-1 (1 exon)
NM_175857
no transcriptome read maps to this gene
2 transcriptome reads map to this gene
| Gene Symbol | SID559 | SID524 | SID557 | SID558 | SID816 | SID1276 |
| --- | --- | --- | --- | --- | --- | --- |
| NCAM2 | 2 | 10 | 0 | 2 | 0 | 9 |
| KRTAP8-1 | 0 | 0 | 0 | 0 | 0 | 0 |
Typical DNA read and direction

## Slide 24
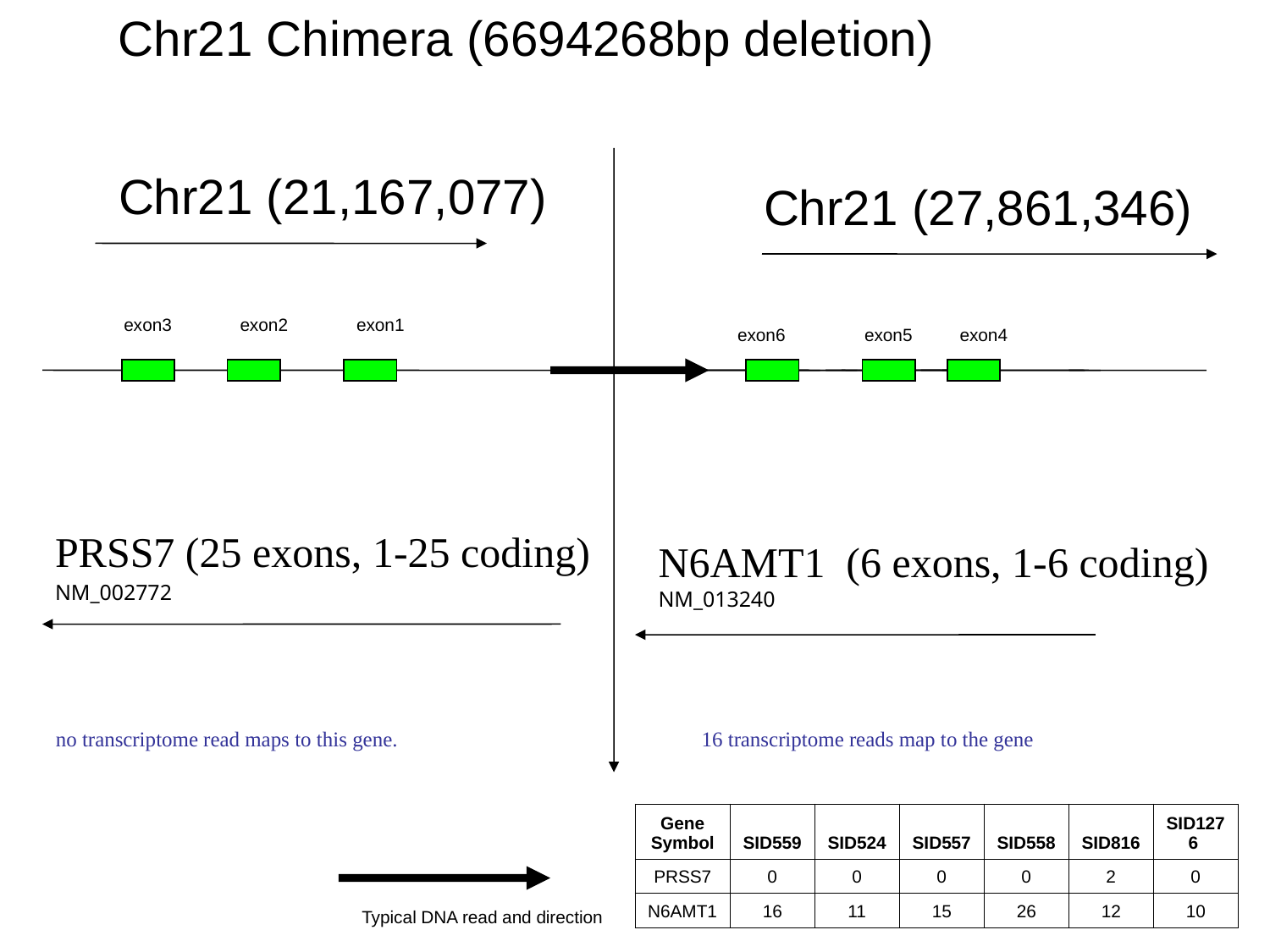

Chr21 Chimera (6694268bp deletion)
Chr21 (21,167,077)
Chr21 (27,861,346)
exon3
exon2
exon1
exon6
exon5
exon4
PRSS7 (25 exons, 1-25 coding)
NM_002772
N6AMT1 (6 exons, 1-6 coding)
NM_013240
| |
| --- |
| |
| --- |
| |
| --- |
no transcriptome read maps to this gene.
16 transcriptome reads map to the gene
| Gene Symbol | SID559 | SID524 | SID557 | SID558 | SID816 | SID1276 |
| --- | --- | --- | --- | --- | --- | --- |
| PRSS7 | 0 | 0 | 0 | 0 | 2 | 0 |
| N6AMT1 | 16 | 11 | 15 | 26 | 12 | 10 |
Typical DNA read and direction

## Slide 25
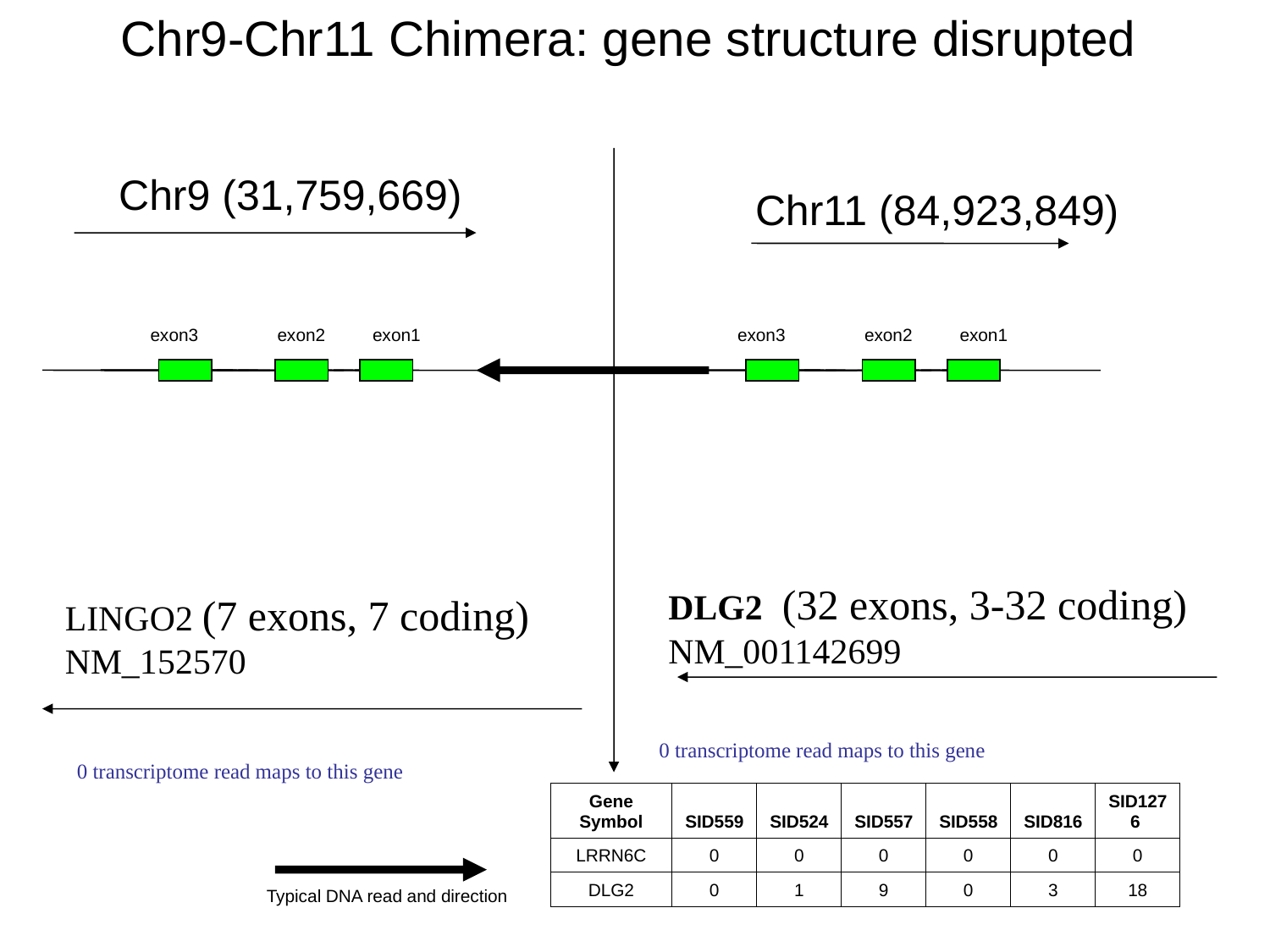

Chr9-Chr11 Chimera: gene structure disrupted
Chr9 (31,759,669)
Chr11 (84,923,849)
exon3
exon2
exon1
exon3
exon2
exon1
| |
| --- |
DLG2 (32 exons, 3-32 coding)
NM_001142699
LINGO2 (7 exons, 7 coding)
NM_152570
0 transcriptome read maps to this gene
0 transcriptome read maps to this gene
| Gene Symbol | SID559 | SID524 | SID557 | SID558 | SID816 | SID1276 |
| --- | --- | --- | --- | --- | --- | --- |
| LRRN6C | 0 | 0 | 0 | 0 | 0 | 0 |
| DLG2 | 0 | 1 | 9 | 0 | 3 | 18 |
Typical DNA read and direction

## Slide 26
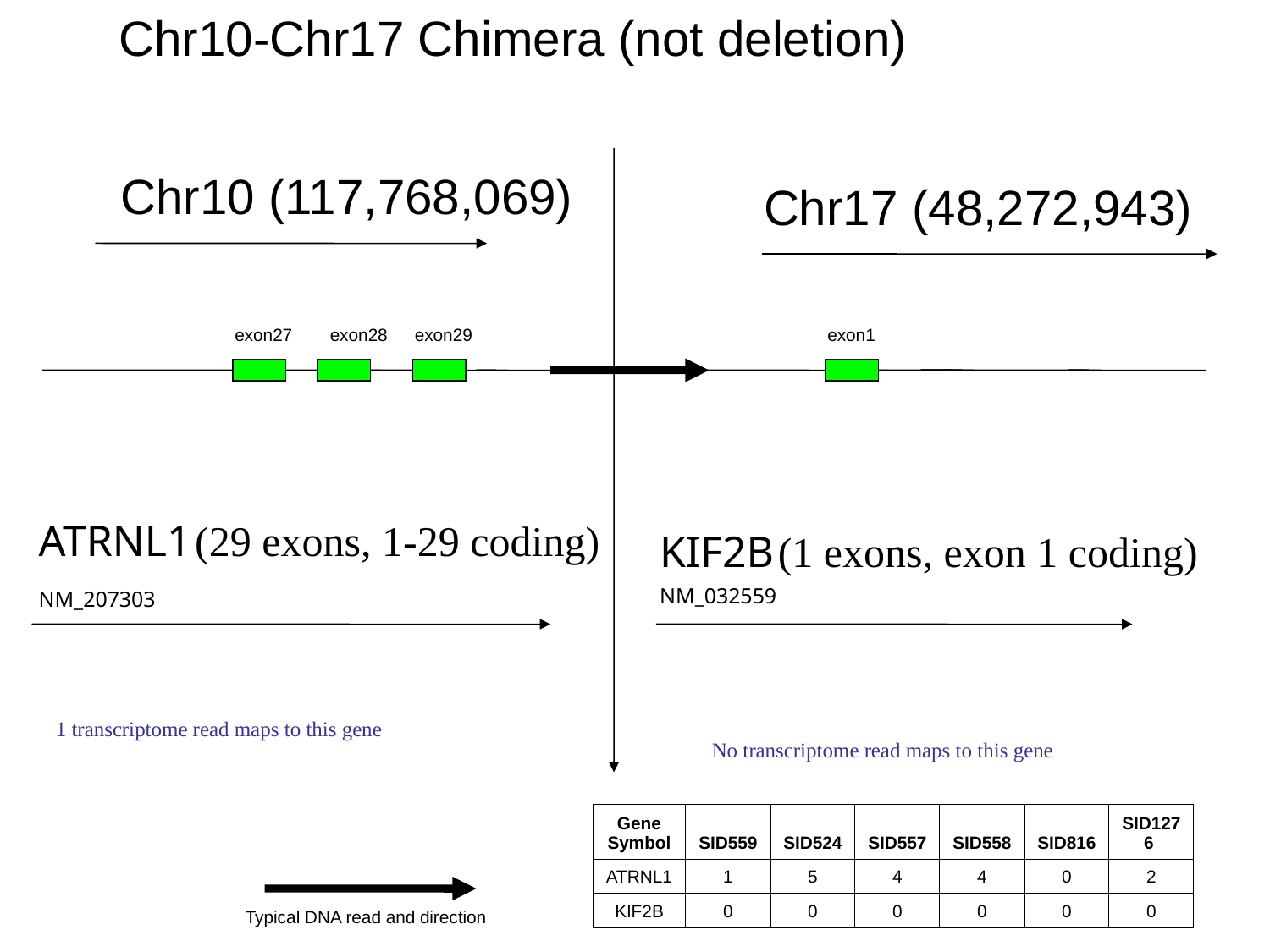

Chr10-Chr17 Chimera (not deletion)
Chr10 (117,768,069)
Chr17 (48,272,943)
exon27
exon28
exon29
exon1
ATRNL1 (29 exons, 1-29 coding)
NM_207303
KIF2B (1 exons, exon 1 coding)
NM_032559
| |
| --- |
| |
| --- |
1 transcriptome read maps to this gene
No transcriptome read maps to this gene
| Gene Symbol | SID559 | SID524 | SID557 | SID558 | SID816 | SID1276 |
| --- | --- | --- | --- | --- | --- | --- |
| ATRNL1 | 1 | 5 | 4 | 4 | 0 | 2 |
| KIF2B | 0 | 0 | 0 | 0 | 0 | 0 |
Typical DNA read and direction

## Slide 27
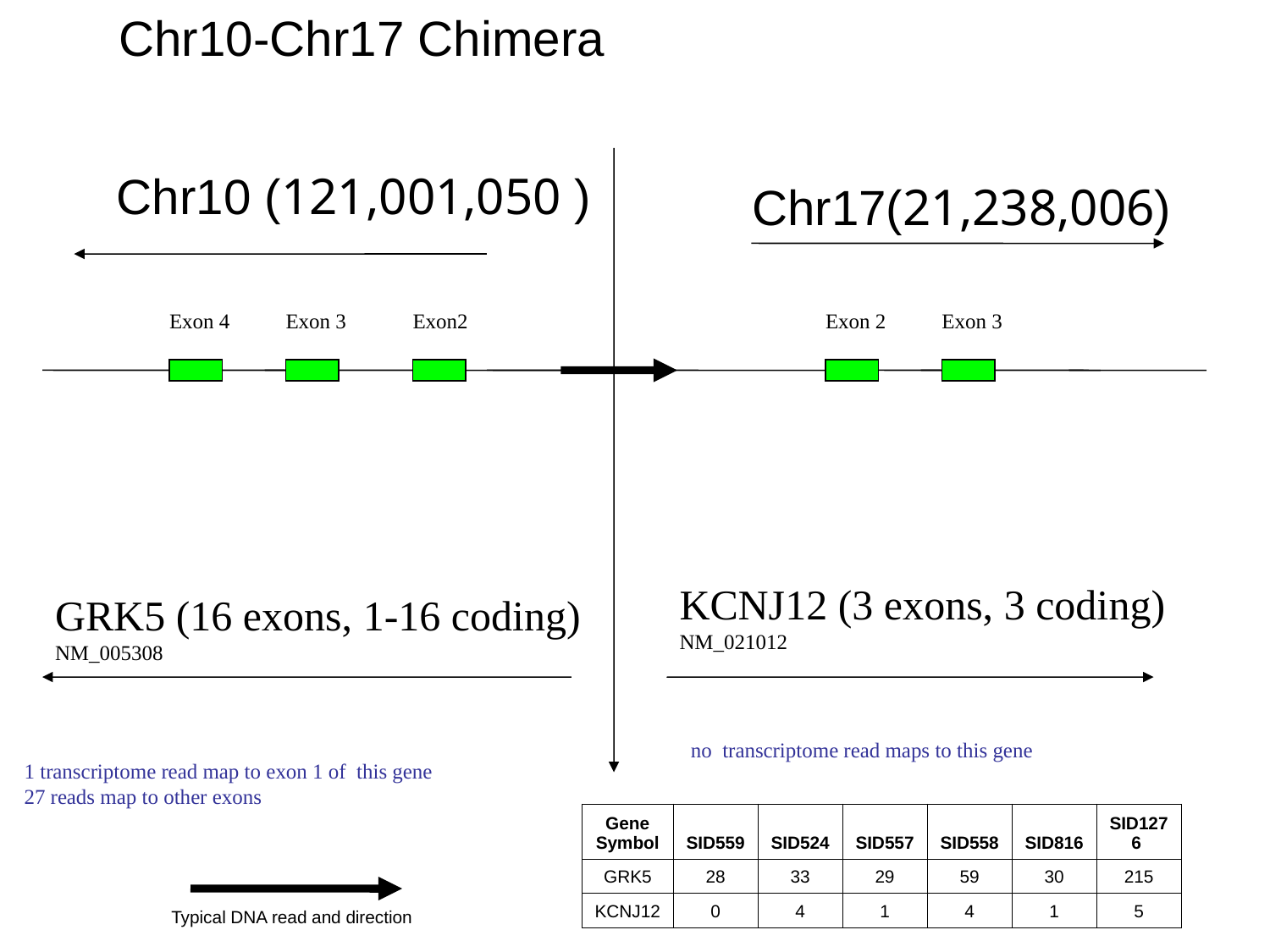

Chr10-Chr17 Chimera
Chr10 (121,001,050 )
Chr17(21,238,006)
Exon 4
Exon 3
Exon2
Exon 2
Exon 3
| |
| --- |
| |
| --- |
KCNJ12 (3 exons, 3 coding)
NM_021012
GRK5 (16 exons, 1-16 coding)
NM_005308
no transcriptome read maps to this gene
1 transcriptome read map to exon 1 of this gene
27 reads map to other exons
| Gene Symbol | SID559 | SID524 | SID557 | SID558 | SID816 | SID1276 |
| --- | --- | --- | --- | --- | --- | --- |
| GRK5 | 28 | 33 | 29 | 59 | 30 | 215 |
| KCNJ12 | 0 | 4 | 1 | 4 | 1 | 5 |
Typical DNA read and direction

## Slide 28
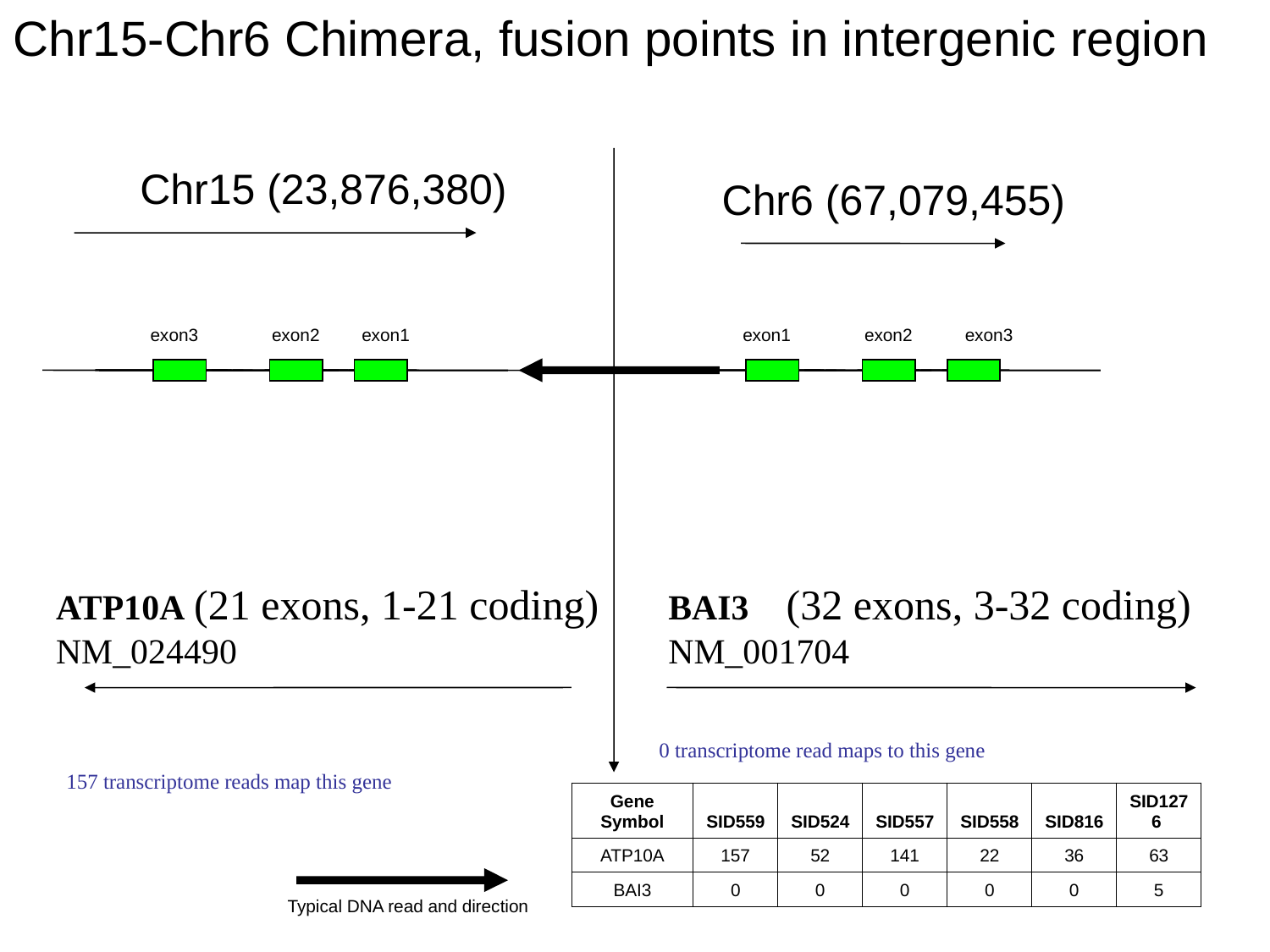

Chr15-Chr6 Chimera, fusion points in intergenic region
Chr15 (23,876,380)
Chr6 (67,079,455)
exon3
exon2
exon1
exon1
exon2
exon3
| |
| --- |
ATP10A (21 exons, 1-21 coding)
NM_024490
BAI3 (32 exons, 3-32 coding)
NM_001704
0 transcriptome read maps to this gene
157 transcriptome reads map this gene
| Gene Symbol | SID559 | SID524 | SID557 | SID558 | SID816 | SID1276 |
| --- | --- | --- | --- | --- | --- | --- |
| ATP10A | 157 | 52 | 141 | 22 | 36 | 63 |
| BAI3 | 0 | 0 | 0 | 0 | 0 | 5 |
Typical DNA read and direction

## Slide 29
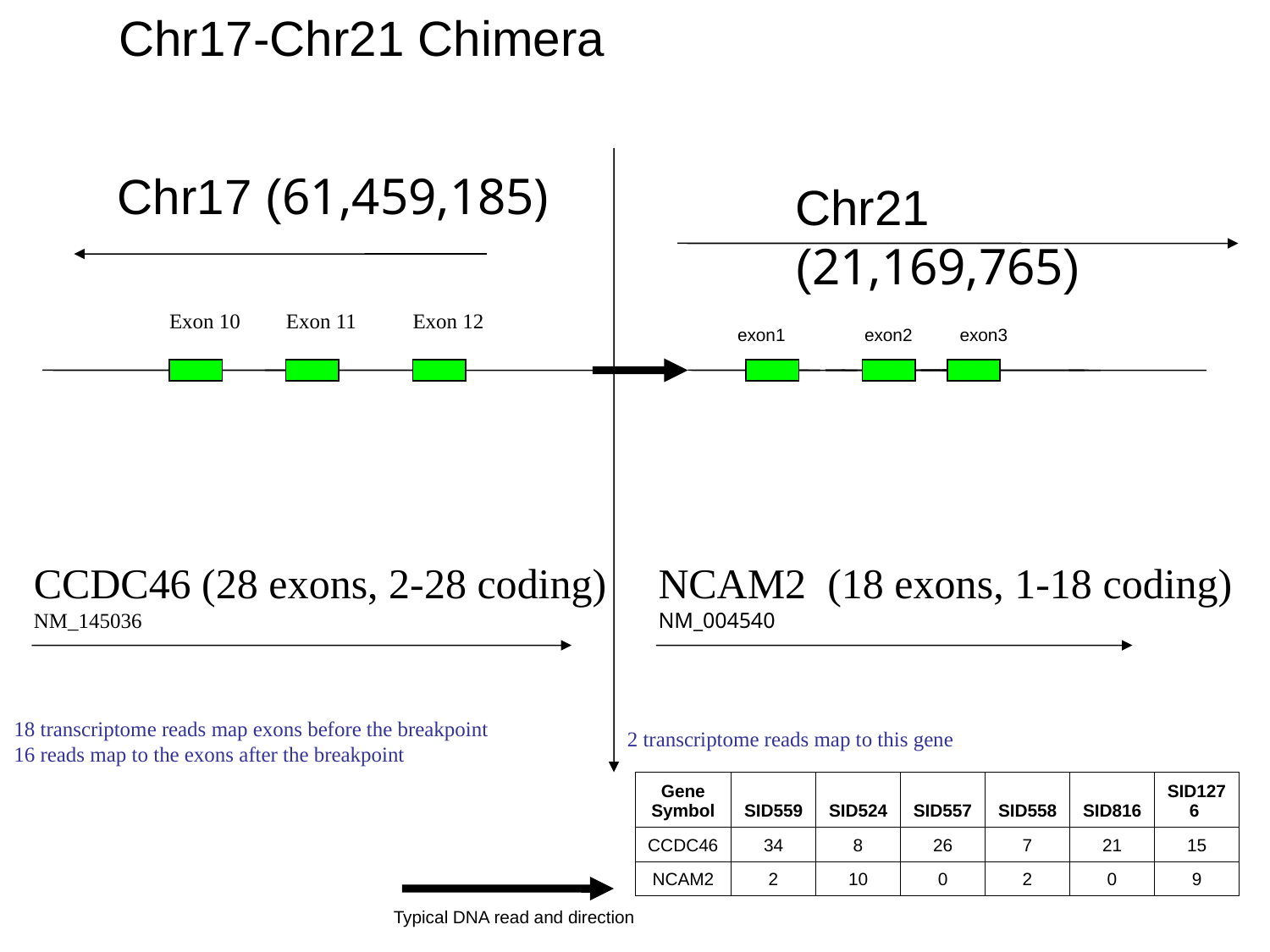

Chr17-Chr21 Chimera
Chr17 (61,459,185)
Chr21 (21,169,765)
Exon 10
Exon 11
Exon 12
exon1
exon2
exon3
| |
| --- |
| |
| --- |
CCDC46 (28 exons, 2-28 coding)
NM_145036
NCAM2 (18 exons, 1-18 coding)
NM_004540
18 transcriptome reads map exons before the breakpoint
16 reads map to the exons after the breakpoint
2 transcriptome reads map to this gene
| Gene Symbol | SID559 | SID524 | SID557 | SID558 | SID816 | SID1276 |
| --- | --- | --- | --- | --- | --- | --- |
| CCDC46 | 34 | 8 | 26 | 7 | 21 | 15 |
| NCAM2 | 2 | 10 | 0 | 2 | 0 | 9 |
Typical DNA read and direction

## Slide 30
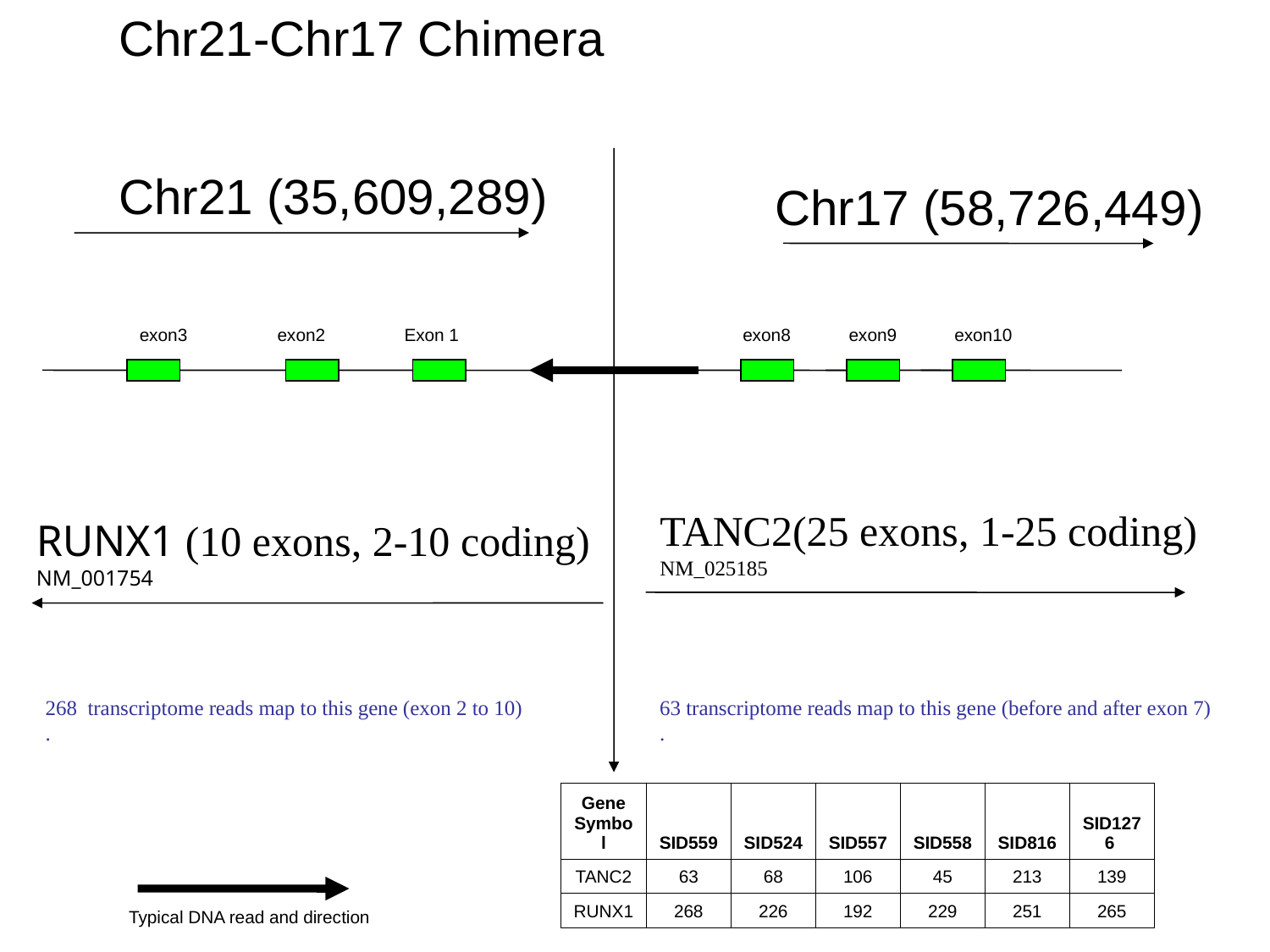

Chr21-Chr17 Chimera
Chr21 (35,609,289)
Chr17 (58,726,449)
exon3
exon2
Exon 1
exon8
exon9
exon10
TANC2(25 exons, 1-25 coding)
NM_025185
RUNX1 (10 exons, 2-10 coding)
NM_001754
268 transcriptome reads map to this gene (exon 2 to 10)
.
63 transcriptome reads map to this gene (before and after exon 7)
.
| Gene Symbol | SID559 | SID524 | SID557 | SID558 | SID816 | SID1276 |
| --- | --- | --- | --- | --- | --- | --- |
| TANC2 | 63 | 68 | 106 | 45 | 213 | 139 |
| RUNX1 | 268 | 226 | 192 | 229 | 251 | 265 |
Typical DNA read and direction
